# Dual-channel optogenetics in yeast for multiplexed light-based control of cellular processes and pathways

**DOI:** 10.1038/s41467-026-73399-0

**Published:** 2026-05-22

**Authors:** Linus Yu Han Tan, Zhangyuan Lin, Jing Wui Yeoh, Jingyun Zhang, Chueh Loo Poh

**Affiliations:** 1 Department of Biomedical Engineering, College of Design and Engineering, National University of Singapore, Singapore, Singapore; 2 NUS Synthetic Biology for Clinical and Technological Innovation (SynCTI), Singapore, Singapore; 3 National Centre for Engineering Biology (NCEB), Singapore, Singapore; 4 Synthetic Biology Translational Research Programme, Yong Loo Lin School of Medicine, National University of Singapore, Singapore, Singapore; 5 Department of Biochemistry, Yong Loo Lin School of Medicine, National University of Singapore, Singapore, Singapore

**Keywords:** Biotechnology, Optogenetics, Gene regulation

## Abstract

Optogenetics which involves the use of light to control cell functions on a genetic level has found utility in studying cell physiology, biomaterials and metabolic engineering. *S. cerevisiae* is an industrially relevant model organism that is used in many applications, but due to the large number of genes required and issues relating to cross-activation between different colours, optogenetics for different wavelengths of light have not been multiplexed in *S. cerevisiae*. In this paper, we develop a compact red light responsive optogenetic system for *S. cerevisiae* that requires only a single gene and no exogenous cofactors. Through engineering modular protein domains, we reduce the cross-activation of our system by blue light. We integrate our red light optogenetic system with EL222 blue light optogenetics to establish dual channel optogenetics in *S. cerevisiae* and demonstrate its utility for engineering biology through the light-based control of flavonoid luteolin synthesis and flocculation for ease of product extraction. We also demonstrate our system’s potential for the development of living materials by producing dual-coloured optogenetic patterns using *S. cerevisiae*. This work expands optogenetic applications in *S. cerevisiae* from single-light to multi-light systems, introducing the potential to multiplex different colours of light for dynamic, orthogonal control of separate cell processes.

## Abstract (web summary)

Optogenetics has found utility in studying cell physiology, biomaterials and metabolic engineering. Here the authors integrate red and blue light optogenetic systems for dual-channel control of S. cerevisiae, demonstrating light-based control of luteolin synthesis and flocculation.

## Introduction

Optogenetics is an emerging field of synthetic biology in which systems are being developed to utilize light to control cell processes^1–4^. Advantages of optogenetics over conventional methods of controlling cellular functions using chemicals include avoiding harmful chemicals^5^, orthogonality to biological systems, dynamic control^6,7^ by introducing and eliminating light as well as spatial resolution^8,9^. As optogenetic systems have been developed for a variety of wavelengths ranging from red^10^, blue^6,11–13^, green^14^ and UV light^15^, a unique added advantage would be the potential to multiplex different colours of light for separate, orthogonal control in cells, placing different functions under the dynamic control of different light ‘channels’^4,8^.

While multiplexed optogenetics have been demonstrated in the model organism *E. coli*^16–18^, as well as in mammalian neurons^19^, due to the lack of compact orthogonal optogenetic systems and issues relating to cross-activation between different colours, only single-channel optogenetics applying one wavelength of light have been applied to *S. cerevisiae* to switch a specific pathway on and off^1,20–22^. *S. cerevisiae* is a powerful model organism, with well-understood genetics and biology, that has been utilized in a variety of synthetic biology applications, such as to produce valuable chemicals^11,23,24^ and medicine^25^, biofuel^26,27^, biomaterials^28,29^, environmental diagnostics^30,31^, alternative food^32–34^ and clinical treatments^35,36^, and would benefit greatly from the additional flexibility and dynamic control afforded by multiple channels of optogenetics^37^. As a eukaryote, it also has distinct advantages over *E. coli*, such as better tolerance to harsh conditions, the capacity for post-translational modifications^38^, and the potential for compartmentalizing metabolic pathways in different organelles^39^.

**Fig. 1:**
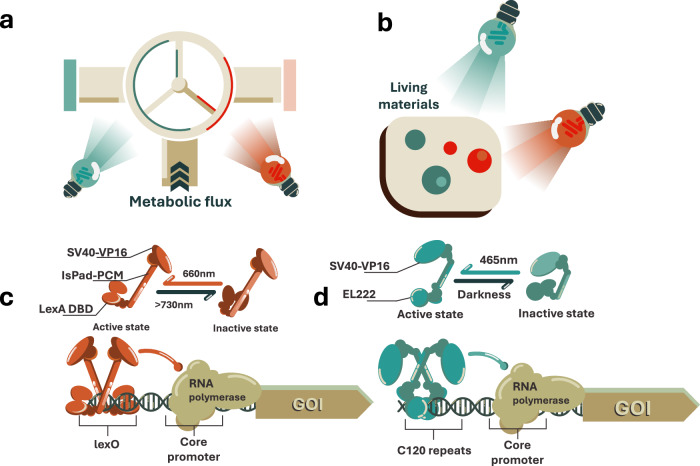
Description of the application and mechanism of two separate optogenetic channels. **a** Dual-channel optogenetics will allow for versatile control over the metabolic flux of organisms being used in bioproduction and can be used for more complicated processes and optimization. **b** Optogenetics has potential use in the engineering of living materials, with two light channels, the localized expression of multiple genes on living materials could be controlled. **c** Depiction of the mechanism of our y-iLight red light channel. **d** Depiction of the mechanism of the EL222 blue light channel.

Here, we choose to utilize a combination of red and blue light-sensitive optogenetics. We successfully engineer a red light optogenetic system with up to 16x induction fold and comparable activity to native yeast promoters. We significantly reduce the cross-activation of the red light optogenetic system by blue light through engineering modular protein domains, and its induction fold improved up to more than 600x. We integrate this system with blue light optogenetics to construct a dual-channel strain and demonstrate several key exemplar applications in the field of bioproduction and biomaterials. This dual-channel optogenetic system enables us to use two wavelengths of light (blue and red) to control the activation of separate genes, either independently or in tandem, providing more dynamic and complex control over cellular functions that would be difficult to achieve using single-channel optogenetics or chemical control. For example, while the temporal resolution of single channel optogenetics is able to redirect metabolic flux towards a single pathway^40^, dual channel optogenetics would be able to distribute metabolic flux over multiple pathways (Fig. 1a). While spatial resolution of single channel optogenetics is able to induce localized gene expression on living materials^4,41^, dual channel optogenetics would allow for more complex patterns of expression involving multiple genes (Fig. 1b).

## Results

### Developing a single-component red light channel in *S.cerevisiae*

Various red light-responsive transcription factors have been developed for *S. cerevisiae*^10,42–44^ as well as for bacteria^45^ and mammalian cells^46^. However, their drawbacks include the need for integration of multiple genes^10,44,45^, addition of exogenous cofactors^42^, or additional genes for the endogenous synthesis of cofactors in vivo^10,44^. Anticipating that the inclusion of too many genes in the dual-channel system may enact metabolic burden on the cell^47,48^, and increase cloning difficulty, we sought a functional single-component red light-sensitive transcription factor for *S. cerevisiae* that could utilize an endogenous cofactor. While no such system had been implemented for *S. cerevisiae* to the best of our knowledge, the iLight system had previously been developed in *E. coli* and mammalian cells^49^. iLight was a single-component, far-red light transcription factor derived by engineering the light-sensitive IsPadC BphP (*Idiomarina* sp. *A28L* phytochrome-activated diguanylyl cyclase) protein^49^. The protein consisted of a DNA-binding domain fused to an evolved photosensory core module (PCM) from IsPadC BphP, which would form homodimers that prevented the dimerisation of the DNA-binding domain. Upon exposure to red light (660 nm), PCM conformation would change to allow the dimerisation of the DNA-binding domains, allowing binding to cognate DNA sequence. Conversely, near-infrared (NIR) light (770 nm) would induce a change from the activated to inactivated state (Fig. 1c). iLight did not require an exogenous cofactor in mammalian cells as it relied on endogenously produced biliverdin IXa. Thus, we reasoned it would function similarly in *S.cerevisiae*, which is known to express a native heme oxygenase Hmx1^50–52^ that catalyses the production of biliverdin.

**Fig. 2:**
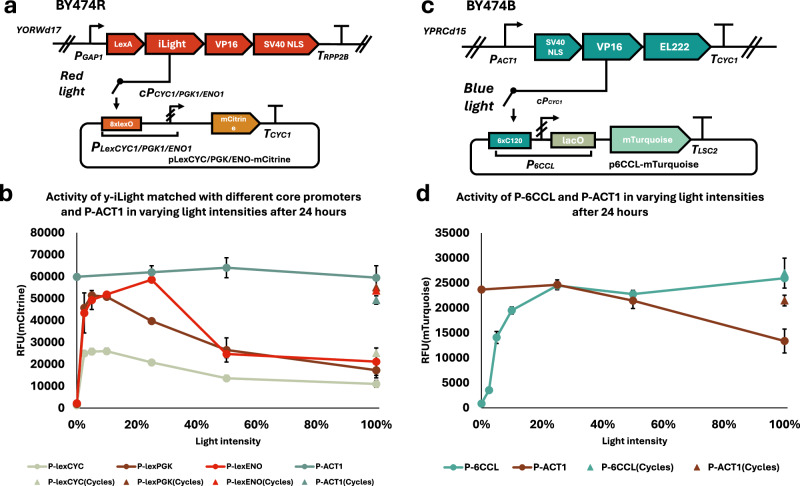
Genetic circuit and testing of red light channels. **a** Genetic circuit of the iLight protein adapted to S.cerevisiae as a red light optogenetic system. **b** Expression of mCitrine fluorescent reporter through y-iLight paired with a lexO array upstream of various core promoters in comparison to constitutive P_ACT1_. BY474R cells were cultured at 30 °C in a shaking incubator while exposed to 0–100% red light (See Supplementary Fig. 19 for operational values of light intensity), as well as cycles of 30 min of 100% red light followed by 30 min of darkness (Represented by the triangle data points). Fluorescence was measured at 24 h and normalized to OD_600_. Error bars indicate standard deviation with a sample size of *n* = 3 centred on the mean. Values are an average of 3 biological replicates. Error bars indicate standard deviation with a sample size of *n* = 3 centred on the mean. **c** Genetic circuit of the EL222 optogenetic system expressed in S.cerevisiae. **d** Expression of mTurquoise fluorescent reporter through EL222 under a P_6CCL_ promoter comparison to constitutive P_ACT1_. BY474B cells were cultured at 30 °C in a shaking incubator while exposed to 0%-100% blue light (See Supplementary Fig. 19 for operational values of light intensity), as well as cycles of 30 min of 100% blue light followed by 30 min of darkness (Represented by the triangle data points). Fluorescence was measured at 24 h and normalized to OD_600_. Values are an average of 3 biological replicates. Error bars indicate standard deviation with a sample size of *n* = 3 centred on the mean. Values are an average of 3 biological replicates. Error bars indicate standard deviation with a sample size of *n* = 3 centred on the mean. Source data for this figure is available in the Source Data file.

The version of iLight demonstrated in bacteria could not be directly adapted into *S. cerevisiae* as the protein lacked the activation domain and nuclear localization sequence required to be functional in eukaryotes. Furthermore, the version demonstrated in mammalian cells would ideally not be used, as it utilized the *S. cerevisiae* Gal4 DNA binding domain, and thus would not be orthogonal to *S. cerevisiae* biology. To engineer an appropriate system for *S. cerevisiae*, we replaced the mammalian variant of iLight’s Gal4 DNA-binding domain with a LexA protein. LexA is commonly used in orthogonal synthetic *S. cerevisiae* transcription factors^53^. Since the bacterial variant utilized a mutated LexA domain, we reasoned that the conformation of the protein would be similarly functional with a wild-type LexA. As the original SV40 NLS had been fused to the Gal4 domain at the N-terminus, the SV40 NLS was re-appended to the C-terminus to produce a synthetic *S. cerevisiae* transcription factor we named y-iLight. Y-iLight was cloned downstream of the constitutive *S. cerevisiae GAP1* promoter (*P*_*GAP1*)_ and integrated into the *S. cerevisiae* genome locus *YORWd17*. A cognate promoter was adapted from Stelling et al^54^, consisting of an array of 8 tandem *lexO* repeats upstream of a *CYC1* core promoter (*cP*_*CYC1*_). To assess the possible impact of varying core promoters on the system^55,56^, two additional plasmids were cloned where *cP*_*CYC1*_ was replaced with either the *PGK1* or *ENO1* core promoter^57^ (*cP*_*PGK1*_ or *cP*_*ENO1*_). These promoters were termed *P*_*lexCYC1*_, *P*_*lexPGK1*_ and *P*_*lexENO1,*_ respectively, and cloned upstream of an mCitrine fluorescent reporter on a low-copy episomal plasmid and transformed into yeast expressing y-iLight (Fig. 2a).

All 3 transformations were cultured on an optogenetics light box (Optobox, see Supplementary Note 15 for device specifications) in 0%, 2.5%, 10%, 25%, 50% and 100% intensities of red light (See Methods for operational units of light intensities). The *ACT1* promoter (*P*_*ACT1*_) is commonly used in both industrial and research applications as a well-characterized promoter for *S.cerevisiae*^58,59^. To gauge whether our engineered promoters could drive sufficient levels of gene expression for practical applications, their activity was compared to *P*_*ACT1*_. Thus, a plasmid with the y-iLight promoter replaced with a constitutive *P*_*ACT1*_ upstream of the mCitrine reporter was used for comparison and cultured in 0%, 25%, 50% and 100% red light intensity as well. When compared to cells kept in darkness, an increase in expression for all three y-iLight responsive plasmids exposed to red light of all light intensities was observed, (Fig. 2b). This indicates that iLight had been successfully refactored to work in *S. cerevisiae* without the need for any additional cofactor.

The different core promoters resulted in different levels of reporter expression, and it was noted that the exposure to red light intensity beyond 10% for *P*_*lexCYC1*_ and *P*_*lexPGK1*_ resulted in a drop in y-iLight activity, while for *P*_*lexENO1*_ red light intensity greater than 50% resulted in a similar drop. OD_600_ for cultures in all conditions did not show any growth defects (Supplementary Fig. 1a), indicating red light had not negatively affected the cells viability. Because this non-linear behaviour is present for all three core promoters, it is likely an artefact of the y-iLight transcription factor itself. One possible explanation for the non-linear behaviour of the red-light promoter is the steric hinderance of active y-iLight, preventing other transcription factors and transcription elements from binding^60^, as binding of transcription activators near the core promoter has been shown to decrease expression due to interfering with the RNA polymerase binding^61^. Another possible mechanism would be ‘facilitated diffusion’, which occurs when, due to crowding of the transcription factor on the DNA strand, and while rate of initial binding increases, residence time for transcription factors decreases, resulting in poor expression from promoter^62,63^. Both proposed mechanisms would manifest as lower expression from a promoter in the presence of high concentrations of active transcription factors.

The three promoters may have differing sensitivities to light as core promoters contain the binding site for RNA polymerase, the transcription start site as well as the region of DNA that the post-initiation complex binds to^64^, and the affinity and activity of these elements are dependent on core promoter sequences. Different core promoters have been shown to vary the overall activity of the promoter^65^, as well as impact the sensitivity of promoters to trans-acting elements^57^. Thus, while not directly affected by light levels, the transcriptional elements that are recruited to a given core promoter may react differently to any given level of y-iLight activation, resulting in differing strengths and behaviour.

When exposed to red light, all promoter variants were able to express mCitrine within the same order of magnitude as *P*_*ACT1*_, demonstrating that their strength was comparable to a native yeast promoter. However, only *P*_*lexENO1*_ at 25% intensity was able to match the expression level of *P*_*ACT1*_ with an induction fold of 26x. To examine if using pulses of red light could alleviate the loss of expression at higher intensities, cells were exposed to cycles of 30 min in 100% red light followed by 30 min of darkness for 24 h (Fig. 2b). These cycles were shown to increase expression for PGK1 and ENO1 core promoters close to that of *P*_*ACT1*_. However, none were able to exceed that of *P*_*lexENO1*_ at 25% light intensity. *P*_*lexENO1*_ was thus chosen for further development, and the strain expressing y-iLight in combination with the *P*_*lexENO1*_ was designated as BY474R, and a constant 25% intensity of red light was used for induction for subsequent experiments. BY474R showed stable expression across exponential phase, stationary and late stationary phase in this condition, comparable to *P*_*ACT1*_, while retaining low expression in darkness (Supplementary Fig. 3).

### Verifying the activity of *EL222* blue light channel

With an appropriate red light-responsive channel established, we next integrate it with a well- established blue light channel. EL222 was an ideal starting point as it has been extensively characterized in *S. cerevisiae* and is simple to clone, requiring only a single transcription factor^40,66–68^. It has also shown other advantageous characteristics, such as fast switching times between activated and deactivated states^66^, linear activity in response to light activity^67^ and scalability in the context of biomanufacturing^40^.

EL222 forms a complex with the metabolite flavin mononucleotide (FMN) in cells. When exposed to blue light (465 nm), the LOV domain cleaves and results in EL222 homodimers that bind to *C120* repeats in DNA sequences. This will bring the VP16 activation domain within sufficient proximity to the core promoter to initiate downstream transcription and subsequent gene expression (Fig. 1d)^69^.

The EL222 system reported by the NUS iGEM team 2021^70^ was used, with EL222 expressed by the constitutive promoter *P*_*ACT1*_, and *P*_*6CCL*_ as the cognate promoter, consisting of six *C120* repeats, a truncated *S. cerevisiae cP*_*CYC1*_ core promoter and a *lacO* sequence inserted downstream of the TATA box. This promoter sequence with *lacO* was inherited from another body of work, which was not used in this particular study, but we felt it was important to highlight this feature for completeness as this would preclude the use of any other lacI-based proteins in conjunction with the system. *P*_*6CCL*_ was cloned upstream of an mTurquoise fluorescent promoter on a low-copy episomal plasmid and transformed into BY4741 with constitutive EL222 expression. The strain was labelled BY474B (Fig. 2c).

BY474B was exposed to blue light on our constructed Optobox with intensities varying from 0%, 2.5%, 5%, 10%, 25%, 50%, and 100%. *S. cerevisiae P*_*ACT1*_ promoter expressing mTurquoise was used as a benchmark and a positive control in 0%, 25%, 50%, and 100% blue light.

BY474B showed increased expression of mTurquoise in blue light, saturating at 25% intensity with an induction fold of 30x (Fig. 2d). Similar to the red light system, we sought to compare its expression level to a commonly used native yeast promoter, to evaluate whether it is strong enough to carry out similar functions. Thus, *P*_*ACT1*_ was also used to express the mTurquoise reporter. Results showed that 100% intensity of blue light had a detrimental but unclear effect on the expression of *P*_*ACT1*_, and further testing suggested this effect extended to many other native promoters. (Data and characterization of the effect of the intense blue light on native promoters can be found in Supplementary Note 16.) To verify that the blue light did not negatively impact the growth of cells, the OD_600_ of cultures was measured in various light conditions used in the study and the results showed that the growth of the cells was unaffected even under 100% blue light (Supplementary Fig. 1). It was also observed that similar to the y-iLight system’s drop in expression, activity could be restored by cycling 30 minutes of 100% light and 30 min of darkness. As 100% blue light affected *P*_*ACT1*_, we decided to limit our comparison of *P*_*6CCL*_ and *P*_*ACT1*_ to the range 0% to 50% blue light intensity, including the dark condition, which is the normal operating condition for *P*_*ACT1*_. Within this range, *P*_*6CCL*_ at its maximum expression was comparable to that of *P*_*ACT1*_, indicating that our blue light system could produce sufficient expression for similar applications.

Thus, it was shown that BY474B was a viable blue light channel in yeast under the blue light intensities evaluated, and blue light intensity would be set at 25% to maximize expression. BY474B showed stable expression across exponential and stationary phase in this condition at levels comparable *P*_*ACT1*_ while maintaining low expression in darkness (Supplementary Fig. 3a). However, expression decreased towards late-stationary phase ( > 48 h), and thus expression from EL222 was not as long lasting as y-iLight.

### Measuring crosstalk between the red and blue optogenetic channels

An ideal multi-colour optogenetic system should have minimal crosstalk, where the wavelengths used to activate one channel do not affect the activated or deactivated state of another^18^. Testing conducted by previous study has indicated that EL222 should not be activated by red light^69^, and iLight should not be activated by blue light^49^.

To verify if crosstalk is absent in our iterations of the BY474B and BY474R systems, they were each cultured in both red and blue light separately, and the fluorescence of their respective reporters was measured and compared to their dark, deactivated state.

**Fig. 3:**
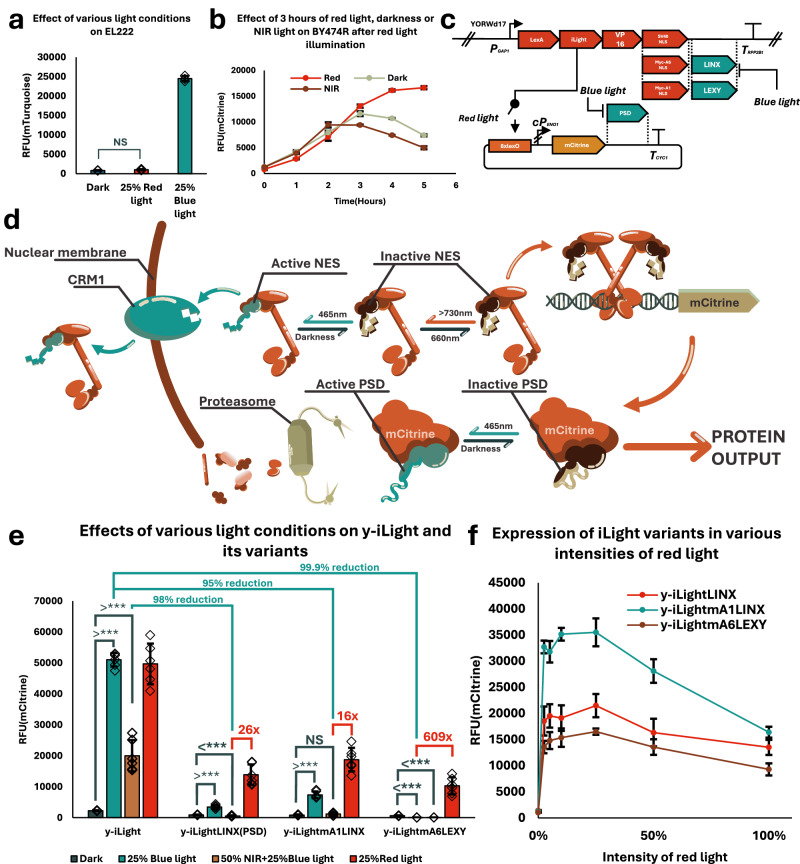
Evaluation of crosstalk between channels and engineering anti-crosstalk measures. **a** Expression of mTurquoise by BY474B in response to red light compared to blue light and darkness. Strain was cultured at 30 °C in a shaking incubator while exposed to 25% red light, 25% blue light or darkness. Fluorescence was measured at 24 h and normalized to OD_600_. Values are an average of 3 biological replicates. Error bars indicate standard deviation with a sample size of *n* = 3 centred on the mean. Results of dark and red conditions were compared pairwise using two-tailed independent *t*-test. **b** Effect of NIR(Near-Infrared) light on y-iLight. BY474R was cultured at 30 °C in a shaking incubator while exposed to 25% red light. After 2 h of this initial exposure, cells were exposed to either continued 25% red light, 50% NIR light or darkness for another 3 hours. Fluorescence was measured every hour and normalized to OD_600_. Values are an average of 3 biological replicates. Error bars indicate standard deviation with a sample size of *n* = 3 centred on the mean. **c** Genetic circuit for the variants of y-iLight tested. **d** Depiction of the mechanism of light-inducible nuclear export sequences and photo-sensitive degrons used to modular engineering. **e** Expression of mCitrine by BY474R in response to blue light and blue plus NIR light, and additional variants of y-iLight engineered for reduced crosstalk. Strains were cultured at 30 °C in a shaking incubator while exposed to 25% red light, 25% blue light or 25% blue light plus 50% NIR or darkness. Fluorescence was measured at 24 hours and normalized to OD_600_. Values are an average of 6 biological replicates. Error bars indicate standard deviation with a sample size of *n* = 6. Results were compared with 2-tailed independent *t*-test, NS = *p* > 0.1, * = *p* < 0.1, ** = *p* < 0.05, *** = *p* < 0.01. Dark blue brackets indicate significance of difference; green brackets indicate reduction in crosstalk, and red brackets indicate fold difference comparing blue light suppressed expression and red light activated expression. *P* values for statistical tests can be found in Table 2. **f** Expression of mCitrine by y-iLightLINX, y-iLightmA1LINX and y-iLightmA6LEXY in various light intensities. Strains were cultured at 30 °C in a shaking incubator while exposed to 0–100% red light. Fluorescence was measured at 24 h and normalized to OD_600_. Values are an average of 3 biological replicates. Error bars indicate standard deviation with a sample size of *n* = 3 centred on the mean. Source data for this figure is available in the Source Data file.

mTurquoise expressed by BY474B in red light was not significantly higher than when in darkness, implying that EL222 was not activated by the conditions of red light that we were using (Fig. 3a). However, BY474R showed 22x higher expression of mCitrine in blue light than in darkness (Fig. 3e), indicating that there was significant crosstalk for the red light channel when exposed to blue light. While lowering the intensity of blue light reduced crosstalk from y-iLight, it also substantially reduced expression from EL222 (Supplementary Fig. 7), and thus was an impractical solution.

It has been earlier demonstrated that conformational changes in the PCM module induced by red light can be forcibly reversed by exposure to NIR (730 nm–850 nm), sufficient to reduce activity even when simultaneously exposed to red light^45,49,71^. BY474R was exposed to 2 h of 25% red light followed by 3 h of 50% NIR light, darkness or continued red light. Hourly measurements showed that NIR light was able to decrease mCitrine expressions from y-iLight further than relying on passive deactivation in dark conditions (Fig. 3b). Thus, we hypothesized that NIR light may similarly be able to curtail the unintended activity in blue light.

To verify the hypothesis, BY474R culture was simultaneously exposed to blue light and NIR light (770 nm) set to 50% intensity. After 24 h, 50% NIR light was able to significantly reduce the activity of the red light channel under blue light exposure (Fig. 3e). However, mCitrine levels remained significantly above the levels recorded under dark conditions. Hence, while NIR light is a viable tool in reducing crosstalk for y-iLight, other methods must be explored to engineer a red channel that is fully blue light independent.

### Modular protein engineering to minimize crosstalk effects of blue light on y-iLight

The next approach that we took was to rationally engineer y-iLight to reduce its activity in blue light by leveraging the modular nature of proteins^72,73^, where multiple domains with differing functions can be fused to produce a protein with synchronous activity. In this case, we focused on domains that could potentially eliminate unwanted activity in blue light. The goal was to decrease crosstalk activation of y-iLight in blue light to statistically similar or significantly less than the levels of activity in dark conditions, without severely compromising the activity in red light. To this end, we made use of photo-sensitive degrons (PSD) developed by Renicke et al^74,75^, consisting of a C-terminal fusion of AtLOV2 domain and an ornithine decarboxylase (ODC) degron. In darkness, folding of the AtLOV2 domain sequesters the ODC degron, but upon blue light exposure, conformational changes in AtLOV2 result in the exposure of the ODC degron and subsequent degradation of the tagged protein by proteosomes^74^ (Fig. 3d). Thus, by fusing the PSD to the downstream product, its expression could be silenced in the presence of blue light.

The second class of modular protein domains that we experimented with were light inducible nuclear export sequences. As a eukaryote, transcription factors in *S. cerevisiae* rely on nuclear localization to be functional^76^, and transport of proteins in and out of the nucleus is mediated by both nuclear localization sequences (NLS) and nuclear export sequences (NES) found on proteins, respectively. An LOV domain fused to the protein of interest sequesters an NES under dark conditions, and upon blue light illumination a conformational change in the LOV exposes the NES, initiating export out of the nucleus^77^. We hypothesized that this could be used to preclude y-iLight activity in blue light, even if activated, as the transcription factor would be unable to access nuclear DNA to initiate transcription (Fig. 3d). Thus, we also tested the reported light-inducible NES domains LINX^78^ and LEXY^79^.

Lastly, we also tested the effect of changing the NLS domains associated with LINX and LEXY. Our initial testing showed LEXY-fused y-iLight to be too weak, while LINX-fused y-iLight still displayed significant crosstalk (Supplementary Fig. 8). It had been shown that the accompanying NLS would affect the overall activity of the light inducible NES^78^, and it was noted that literature had characterized the Myc-A6 NLS used by LINX to have a 5-fold stronger affinity for nuclear import protein as compared to the Myc-A1 NLS used by LEXY^80^. We thus hypothesized that by swapping the NLS sequences for the two nuclear export tags, the change in nuclear import levels could offset each system’s shortcomings, producing light inducible NES modules that were of more moderate activity.

Eleven combinations of protein modules were tested in darkness, red light, blue light and blue plus NIR light (Fig. 3c and Table 1). From the results, three combinations of modules were shortlisted as the best performing, designated y-iLightLINX(PSD), y-iLightmA1LINX and y-iLightmA6LEXY (Table 2) (see Supplementary Note 5 for full engineering process and selection criteria). Comparing expression under blue plus NIR light, y-iLightLINX(PSD) had a 98% reduction in expression compared to the original y-iLight. y-iLightLINX(PSD) had significantly less expression in blue plus NIR light compared to darkness, and increased expression by 26-fold under red light, but required PSD to be fused to downstream protein.

**Table 1:** List of y-iLight variants tested

Name	Fusion to mCitrine protein	NLS	y-iLight fusion
y-iLight	-	SV40	-
y-iLightPSD	PSD	SV40	PSD
y-iLightPSD(PSD)	PSD	SV40	PSD
y-iLightLINX	-	mA6	LINX
y-iLightLINX(PSD)	PSD	mA6	LINX
y-iLightLEXY	-	mA1	LEXY
y-iLightLEXY(PSD)	PSD	mA1	LEXY
y-iLightmA1LINX	-	mA1	LINX
y-iLightmA1LINX(PSD)	PSD	Ma1	LINX
y-iLightmA6LEXY	-	mA6	LEXY
y-iLightmA6LEXY(PSD)	PSD	mA6	LEXY

**Table 2:** Shortlisted variants of y-iLight resulting from modular protein engineering

Name	Expression in red light	Crosstalk eliminating condition (CEC)	Expression in CEC	Reduction in crosstalk under CEC	Fold change in red light vs CEC	p-value in t-test between dark and	
						Blue light	Blue + NIR light
y-iLight	49675(±6578)	-	20001( ± 5058)	-	-	9.80 × 10^−14^	6.36 × 10^−6^
y-iLightLINX(PSD)	13863(±3338)	Blue+NIR	528( ± 229)	98%	26-fold	1.12 × 10^−6^	0.00453
y-iLightmA1LINX	18756(±3805)	Blue+NIR	1178( ± 445)	95%	16-fold	4.60 × 10^−8^	0.114
y-iLightmA6LEXY	10294(±2761)	Blue	16( ± 66)	99.9%	609-fold	3.81 × 10^−7^	2.78 × 10^−7^

Expression in red light indicates the level of mCitrine produced after 24 h of 25% red light exposure. Crosstalk eliminating condition(CEC) indicates the minimal condition where crosstalk from blue light is either not significantly different from or less than baseline expression in darkness. Reduction in crosstalk under CEC indicates the percentage reduction in mCitrine expression under CEC compared to the expression of mCitrine by y-iLight in the equivalent condition.

Comparing expression under blue plus NIR light, y-iLightmA1LINX had a 95% reduction in expression compared to the original y-iLight, and negligible difference between dark and blue plus NIR conditions without the need for PSD fusion. When exposed to red light, expression increased by 16-fold.

Under blue light only, y-iLightmA6LEXY had a 99.9% reduction in expression compared to the original y-iLight. y-iLightmA6LEXY was able to reduce expression of mCitrine to levels significantly less than baseline in blue light, not requiring NIR light nor PSD fusion (Fig. 3e). In comparison to exposure to blue light, y-iLightmA6LEXY increased expression by 609-fold in red light. While crosstalk measures had significantly reduced crosstalk, they had also significantly decreased expression in the red light-only condition, 21–38% of the original (Fig. 3e).

To fully characterize the behaviour of these selected variants, they were exposed to increasing intensities of red light. They produced similarly non-linear response curves, with reporter expression increasing proportionately to light intensity up to 25%, and subsequently decreasing in expression for higher intensities (Fig. 3f). y-iLightmAILINX demonstrated the highest expression, followed by y-iLightLINX(PSD), and y-iLightmA6LEXY produced the lowest expression (Fig. 3f).

### Implementation of the dual-channel optogenetics in a single strain

Four variants of the dual-channel optogenetic strains were constructed by integrating the BY474B system in the same strain as the original y-iLight, designated OY-α (Opto-Yeast), as well as each of the three selected y-iLight variants, designated OY-β.LINX, OY- β.mA1LINX and OY- β.mA6LEXY in accordance with their names in Table 1. mTurquoise and mCitrine remain the reporters for the blue light and red light channels, respectively, from their episomal, low-copy plasmids. EL222 was used to drive the blue channels, while y-iLight and its variants were used to drive the red light channel.

**Fig. 4:**
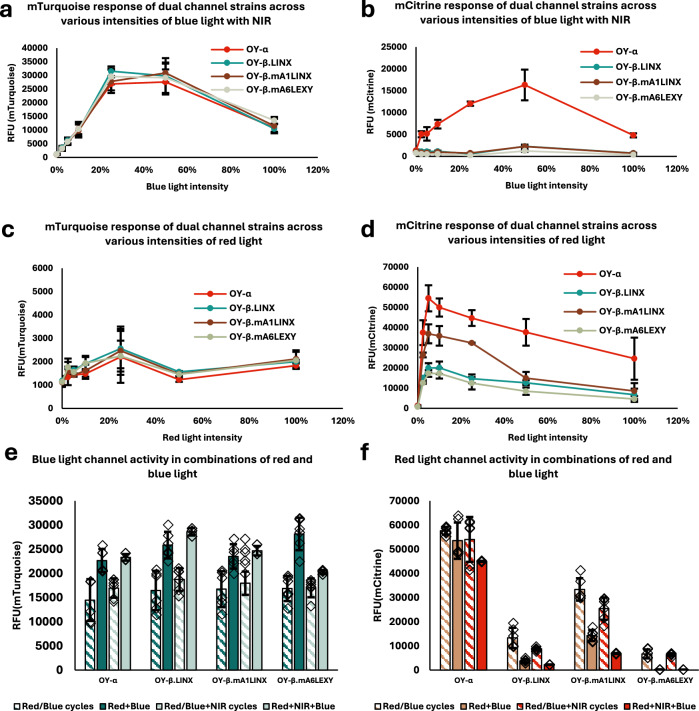
Testing of dual-channel strains. **a** Expression of mTurquoise and (**b**) expression of mCitrine in OY-α, OY-β.LINX, OY- β.mA1LINX and OY- β.mA6LEXY in increasing intensities of blue plus NIR(Near-Infrared) light. Strains were cultured at 30 °C in a shaking incubator and exposed to 50% NIR with 0-100% of blue light. Fluorescence was measured at 24 h and normalized to OD_600_. Values are an average of 3 biological replicates. Error bars indicate standard deviation with a sample size of *n* = 3 centred on the mean. **c** Expression of mTurquoise and **d** expression of mCitrine in OY-α, OY-β.LINX, OY- β.mA1LINX and OY- β.mA6LEXY in increasing intensities of red light. Strains were cultured at 30 °C in a shaking incubator and exposed to 0-100% of red light. Fluorescence was measured at 24 h and normalized to OD_600_. Values are an average of 3 biological replicates. Error bars indicate standard deviation with a sample size of *n* = 3 centred on the mean. **e** Expression of mTurquoise and **f** expression of mCitrine in OY-α, OY-β.LINX, OY- β.mA1LINX and OY- β.mA6LEXY in conditions involving multiple lights. To test the simultaneous activation of both channels, strains were cultured at 30 °C in a shaking incubator and exposed to 60 min cycles of 25% red light followed by 25% blue light, or 25% red light followed by 25% blue plus 50% NIR light, as well as 25% red plus 25% blue light simultaneously and all three lights simultaneously. Fluorescence was measured at 24 h and normalized to OD_600_. Values are an average of 6 biological replicates. Error bars indicate standard deviation with a sample size of *n* = 6. Source data for this figure is available in the Source Data file.

To verify the activity of each channel, the strains were cultured in increasing intensities of both 0-100% blue light supplemented with 50% NIR light, as well as 0-100% red light, and the fluorescent output of each channel measured (Fig. 4a–d).

mTurquoise was successfully induced by blue light in all strains to a similar capacity, and similar to BY474B, demonstrated increasing expression proportionate to blue light up to 25%, where subsequent increase in intensity did not further increase expression (Fig. 4a), demonstrating successful integration of the blue light channel. However, unlike BY474B, expression at 100% blue light had dropped off instead of plateauing, which may be attributed to the increased metabolic burden of maintaining the genes necessary for both channels. The combination of blue and NIR light resulted in expression of mCitrine through the red light channel in OY-α, but for the other three strains, expression remained low through all intensities of blue light (Fig. 4b), demonstrating that the integration of both channels did not affect the crosstalk elimination measures.

As expected, increasing intensities of red light did not cause any increase in expression of mTurquoise (Fig. 4c), and thus there remained no unspecific activation of the blue light channel by red light. Increasing intensities of red light produced non-linear expression curves similar to BY474R and the single channel y-iLight variants seen in Fig. 3f. However, the peak of the curve has shifted left with expression of mCitrine increasing proportionally to red light up to 5% intensity and subsequently decreasing with increased intensities of red light for all strains (Fig. 4d). Consistent with the blue light channel, this decreased expression could be a result of increased metabolic burden of maintaining more genes in the dual-channel strain.

The relative strength of the red light channels matched that of the single channel y-iLight variants seen in Fig. 3f, OY-β.mA1LINX demonstrated the highest expression, followed by OY-β.LINX and OY- β.mA6LEXY produced the lowest expression (Fig. 4d). These trends in the relative expression of the blue and red light channels under blue plus NIR light, red light and in darkness remained consistent across the exponential, stationary and late-stationary phases (Supplementary Figs. 4–6).

To evaluate the ability of dual channel strains to activate both channels simultaneously, an additional four conditions were tested: alternating cycles of 60 min of red followed by blue light (Red/Blue cycles), simultaneous red and blue light (Red plus Blue), alternating cycles of 60 min of red followed by blue plus NIR light (Red/Blue plus NIR cycles), and simultaneous red, NIR and Blue light (Red plus NIR plus Blue).

Results show that the use of 60 min duty cycles reduced the output of the blue light channel compared to simultaneous exposure to red and blue light with or without NIR light (Fig. 4e). This was expected, as we previously demonstrated that the activity of EL222 was proportional to the amount of blue light present. All dual-channel strains presented similar levels of blue channel activity in all four conditions.

The use of duty cycles or simultaneous exposure to red and blue light with or without NIR light did not affect red channel activity of OY-α, with all four conditions producing similar levels of mCitrine, which was expected as both blue and red light would activate the default version of y-iLight. However, the simultaneous use of red and blue with or without NIR light severely reduced the red light channel output of OY-β.LINX, OY-β.mA1LINX and OY-β.mA6LEXY. It was likely that the crosstalk measures used to prevent y-iLight variants from being unintentionally activated in blue light alone also suppressed their red light-induced activity when exposed to both light wavelengths. This was alleviated in the 60 min duty cycles, with all three strains showing increased expression. We hypothesized that using duty cycles provided an opportunity for y-iLight variants to initiate transcription during the 60 min of red light, and while transcription was suppressed during the 60 min of blue and NIR light, mRNA produced continued to be translated, and protein products remained stable across the 60 min. Duty cycles of 30 and 15 minutes were also tested (Supplementary Fig. 9), but 60 min cycles provided the best results. This successfully represents a condition where products of both channels can be present at the same time.

Comparing the dual channel strains, OY- β.mA1LINX demonstrated the ability to successfully express either the red or blue light channel without significant crosstalk with the assistance of NIR light, and without the need for a PSD module to be fused to its downstream gene product. It was able to simultaneously express both channels in tandem using duty cycles with the highest expression from each channel. Thus, we considered that OY- β.mA1LINX was the most suitable for further development as a dual channel optogenetic yeast.

### Applying dual-channel optogenetic yeast for pathway control in metabolic engineering

In metabolic engineering, optimizing metabolic flux through different pathways is achieved through fine-tuning the expression and stable state levels of different enzymes^81,82^. Earlier we had demonstrated the ability of multiplexed optogenetics to express fluorescent reporters. In this section, we aim to develop this further beyond inducing protein expression and demonstrate dynamic control of metabolic pathways. Optogenetics provides the opportunity to express genes in both a step-wise and time-resolved manner^40,83,84^, and dual channel optogenetics confers the added benefit of multiplexed control in the model industrial chassis *S. cerevisiae*, which allows for more complex pathways with multiple nodes to be controlled independently^18^. Thus, it can be used as a tool to generate a variety of induction conditions to gain deeper insights into the behaviour of biosynthesis pathways that would have otherwise been difficult to ascertain using native constitutive or chemically inducible promoters.

As proof of application, we sought to use OY- β.mA1LINX to control the expression of various genes in an industrially relevant pathway (e.g., bioproduction of flavonoids, which offer health benefits), and using the dual light channel system to create a variety of induction conditions.

**Fig. 5:**
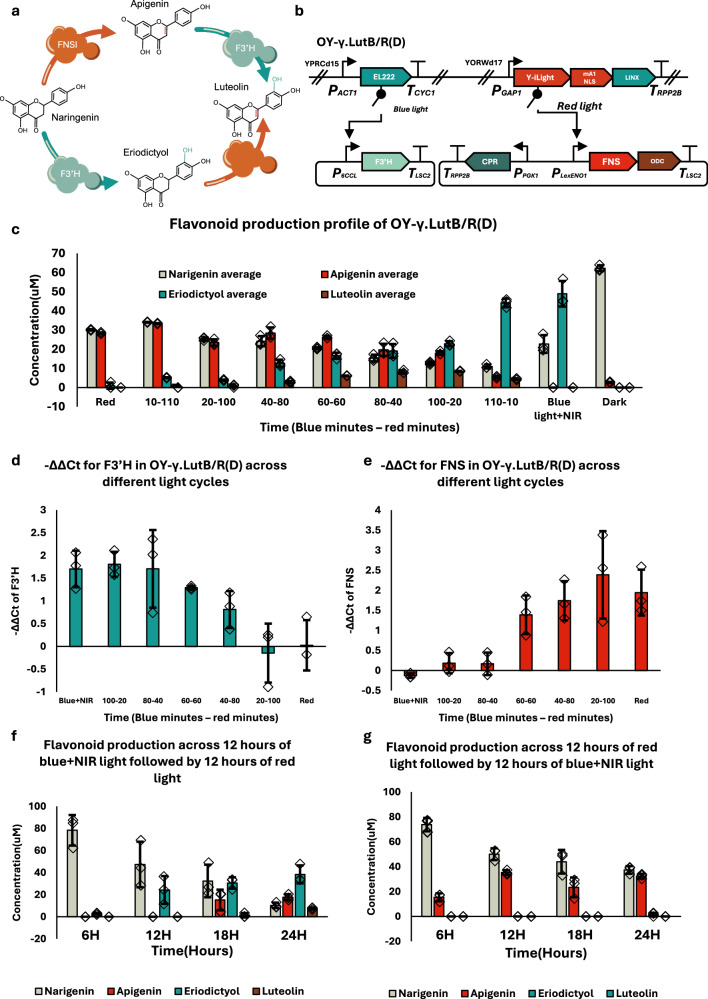
Application of dual channel strain to luteolin production. **a** Enzymatic conversion of naringenin to luteolin through a diamond shaped pathway. **b** Genetic circuit for OY- γ.LutB/R(D). **c** Flavonoid production of OY- γ.LutB/R(D) in varying cycles of red and blue plus NIR(Near Infrard) light. Strain was cultured at 30 °C in a shaking incubator in cycles with a period of 120 min, with the first number indicating the minutes exposed to blue plus NIR light and the second number indicating the minutes exposed to red light. After 24 h, flavonoids were extracted and characterized with HPLC. Values are an average of 3 biological replicates. Error bars indicate standard deviation with a sample size of *n* = 3 centred on the mean. **d**, **e** RT-qPCR results for F3’H expression. OY- γ.LutB/R(D) was cultured at 30 °C in a shaking incubator while exposed cycles with a period of 120 min, with the first number indicating the minutes exposed to blue plus NIR light and the second number indicating the minutes exposed to red light. After 5 h, RNA was extracted, and RT-qPCR was used to qualify F3’H (**d**) and FNSI (**e**) relative to UBC6 housekeeping gene. ΔCt of these light conditions was compared to ΔCt of cells kept in dark, and the difference was plotted on the graph. Values are an average of 3 biological replicates. Error bars indicate standard deviation with a sample size of *n* = 3 centred on the mean. **f** OY- γ.LutB/R(D)was cultured at 30 °C in a shaking incubator and exposed to 12 h of 25% blue plus 50% NIR light for 12 h, followed by 12 h of 25% red light. Every 6 h, flavonoids were extracted and characterized with HPLC. Values are an average of 3 biological replicates. Error bars indicate standard deviation with a sample size of *n* = 3 centred on the mean. **g** OY- γ.LutB/R(D)was cultured at 30 °C in a shaking incubator and exposed to 12 h of 25% red light for 12 h, followed by 12 h of 25% blue plus 50% NIR. Every 6 h, flavonoids were extracted and characterized with HPLC. Values are an average of 3 biological replicates. Error bars indicate standard deviation with a sample size of *n* = 3 centred on the mean. Source data for this figure is available in the Source Data file.

To this end, we utilized the catalytic conversion of naringenin into luteolin in our demonstration with a focus on better understanding the characteristics of this pathway. The conversion of naringenin to luteolin is a unique pathway, as it can be catalysed through two enzymes, flavone synthase I (FNSI) and flavanone 3’ hydroxylase (F3’H)^85,86^, but the reaction is commutative and forms a diamond shaped pathway. Naringenin can either be catalysed by FNSI to apigenin, where apigenin is further catalysed to luteolin by F3’H, or it can be first catalysed by F3’H to eriodictyol, where it is further catalysed by FNSI to luteolin (Fig. 5a). While luteolin had previously been produced in *S. cerevisiae*, all enzymes in the pathway were constitutively expressed^87^. Consequently, no data could be drawn on how the differential flux between the two possible pathways may affect the yield of luteolin.

As previously determined, OY- β.mA1LINX was used as the basis for constructing the luteolin producing strain. We designed F3’H to be expressed through the blue channel, while FNSI was to be expressed through the red channel, producing the strain OY- γ.LutB/R(D) (Fig. 5b). Constitutively destabilizing ODC was appended to FNSI to reduce leaky activity and cytochrome P450 reductase was constitutively expressed (See Supplementary Note 7 for optimization and construction details).

To demonstrate dose-dependent and step-wise induction of a metabolic pathway, we examined the effect of different proportions of light exposure on the flux of the luteolin synthesis pathway. OY- γ.LutB/R(D) was cultured for 24 h with 75 uM of naringenin as a substrate, and tested in an array of conditions: (i) darkness, (ii) blue plus NIR light, (iii) red light, and (iv) differing duty cycles with a period of 120 min with a range of ratios of blue plus NIR to red light (Fig. 5c).

When OY- γ.LutB/R(D) was exposed to varying duty cycles (Fig. 5c), and the amount of apigenin produced increased with the proportion of red light in the duty cycle (5.4–33.4 μM), corresponding to a likely increase in FNSI expression. The amount of eriodictyol produced conversely increased with the proportion of blue light in the duty cycle (5.1–49μM). Luteolin was undetectable for either red light only or blue plus NIR light only, which corroborates the need for both enzymes to complete the pathway to produce luteolin. The maximum luteolin yield achieved was 8.5 μM, achieved on slightly blue-skewed duty cycles of 100 min blue plus NIR light followed by 20 min of red light. To verify that the increase in proportion of blue or red light resulted in a proportionate increase in enzyme expression, RT-qPCR was used to quantify the amount of F3’H and FNSI transcripts during culturing in different light cycles compared to dark conditions, with UBC6 as a reference gene. As expected, an increase in blue light exposure resulted in increased F3’H transcript (Fig. 5d) and an increase in red light exposure resulted in increased FNSI transcript (Fig. 5e). This demonstrates the successful use of dual-channel optogenetics in yeast to direct the flux of a substrate into two distinct metabolic pathways in adjustable proportions.

To study the effect of time-resolved expression of enzymes on the luteolin pathway, the effects of expressing the enzymes during separate 12 h phases were also examined. OY- γ.LutB/R(D) was cultured in either 12 h of blue plus NIR light followed by 12 h of red light, or 12 h of red light followed by 12 h of blue plus NIR light, and flavonoid concentrations were measured at 6 h intervals. Interestingly, the order of expression of each pathway yielded vastly different results.

Strains exposed to blue plus NIR light first followed by red light, produced eriodictyol in the first 12 h, and only produced apigenin in the last 12 h after red light exposure, as expected (Fig. 5f). After 24 h, strains exposed to blue plus NIR light first produced less amount of apigenin (17.7 μM) but more eriodictyol (38.4 μM) and luteolin (7.5 μM). However, strains that were exposed to a phase of red light first produced no detectable luteolin, while 58 μM of apigenin was produced with minimal amount of eriodictyol (1.4 μM) produced even in the last 12 h (Fig. 5g).

Through this phase-based testing, we can identify a key bottleneck in production of luteolin. Contrasting Fig. 5f, g, it is implied that the F3’H enzyme did not catalyse any reaction with naringenin or apigenin the last 12 h of the 24-h cycle (See Supplementary Fig. 12 for the full range of time blocks tested), and thus its functionality is limited to the early stages of the cell culture.

This would have not been possible to discern with constitutive promoters, and implementing the same pattern of expressing genes in blocks of time with chemically inducible promoters would require constant changes in media. In contrast, we are able to activate and deactivate the expression of either pathway within a single experiment and study the branches of this pathway in both a time-resolved and continuous manner.

**Fig. 6:**
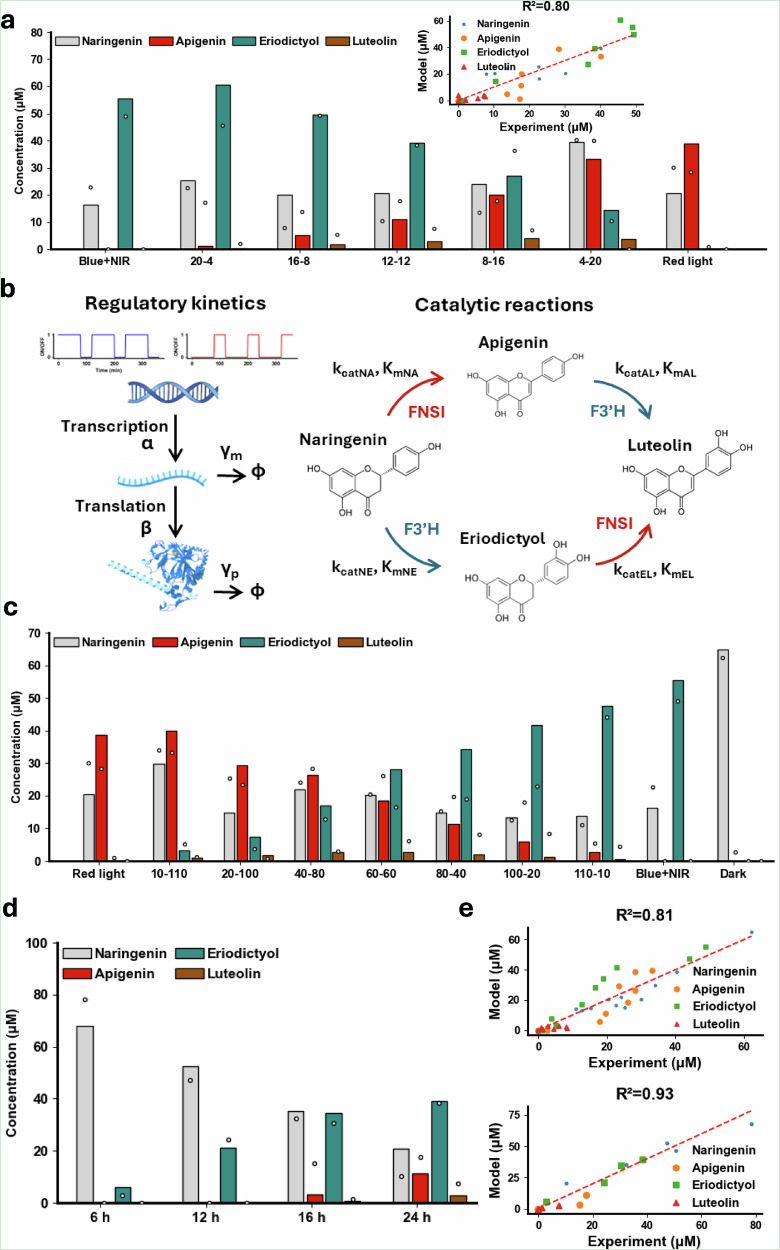
In silico model simulations to derive reaction kinetics of flavonoid bioconversion. **a** Simulated flavonoid concentrations when exposed to one cycle varying proportions of blue plus NIR(Near-Infrared) light followed by red light illumination (*n* = 1). **b** A schematic diagram of the model elucidating the regulatory mechanisms and reaction kinetics of the two pathways for producing luteolin from naringenin. **c** Predicted flavonoid levels under different duty cycles of blue plus NIR light followed by red light at a period of 120 min for 24 h (*n* = 1). **d** Predicted time-response profiles under a [12] h illumination cycle (*n* = 1). **e** Computed *R*^2^ (Coefficient of determination) values upon comparing model predictions from (**c**, **d**) with experimental data. *The dots on the bar charts denote the experimental data for comparison. Source data for this figure is available in the Source Data file.

To further capitalize on the data generated through optogenetic experiments and to quantitatively dissect the influence of light duty cycles in regulating the differential flux of the two pathways, in parallel, we developed an in silico mathematical model to capture the interplay between light-regulated enzyme expression and flavonoid bioconversion dynamics. Leveraging data from a single illumination cycle exposed to varying proportions of blue plus NIR light followed by red light, our model successfully recovered the general patterns of the distinct flavonoid accumulation profiles observed at 24 h (Fig. 6a), achieving a high coefficient of determination (*R*^2^ = 0.80), demonstrating a good agreement with the experimental results.

To validate the model’s predictive performance and generalizability, we deployed the developed model to predict the four flavonoid profiles subjected to an independent data of differing duty cycles with a period of 120 minutes over 24 hours (Fig. 5c). Notably, the resultant model estimates closely mirrored the experimental measurements, attaining high *R*^2^ of 0.81. This high degree of concordance underscores the model capacity to reliably capture and explain over 80% of the observed data variance, highlighting its potential as a powerful tool for guiding light-based metabolic control strategies.

We further assessed the model performance by investigating the model capability in predicting the time-response dynamics. Remarkably, the model recapitulates the temporal profiles for the four different flavonoids with a high *R*^2^ of 0.93. These exceptional levels of agreement reinforce the predictability and generalizability of the developed model in explaining the complex light patterns from hours to minutes, and even the temporal dynamics.

Built on the validated model, we then analyzed the differential reaction kinetics between the two pathways derived from the estimated model parameters. The model reveals that the catalytic rate (*k*_cat_) of FNSI enzyme converting naringenin to apigenin is higher ( ~ 1.4x) than F3’H enzyme to eriodictyol. Additionally, the FNSI exhibits higher binding affinity ( > 1.6x), indicated by lower *K*_m_, to naringenin relative to F3’H. Meanwhile, the model estimates that the catalytic activity of F3’H for catalysing apigenin to luteolin is two orders of magnitude lower than that of FNSI for catalysing eriodictyol to luteolin, albeit F3’H displays a marginal better binding affinity to apigenin than FNSI to eriodictyol.

To better account for the potential compensation effect between catalytic activity and binding affinity, we then analyze the catalytic efficiency, quantified by the ratio *k*_cat_/*K*_m_, a robust metric for comparing enzyme activity across different substrates or enzymes. Our model reveals the order of the enzyme efficiency for the four reactions in the rank: (Naringenin → Apigenin) > (Naringenin → Eridictyol) > (Eriodictyol → Luteolin) > (Apigenin → Luteolin). Interestingly, this quantitative discovery aligns with experimental observations, which suggest that F3’H enzyme might not function properly with diminished transcriptional activity at the later phase of the culture.

These results highlight the potential of dual channel optogenetics as a powerful tool for dissecting the reaction kinetics of pathways in bioproduction, which is often challenging when expressing using constitutive promoter or chemical inducers for temporal control. More importantly, when coupled with rigorous model fitting and prediction, the dual channel optogenetics system enables reliable extraction of kinetic and regulatory parameters, providing valuable mechanistic insights into pathway control.

### Applying dual-channel optogenetics for time-separated phases in biomanufacturing

An advantage of optogenetics is temporal resolution, where light can be administered and removed easily to demarcate different phases of gene expression, a concept that we earlier demonstrated in the phase-based expression of the luteolin pathway. Another common use case involves utilising single-channel optogenetics to separate growth and protein production phases^21^. With dual-channel optogenetics, more complex control can be applied with more distinct phases. Thus, as a second proof of application, we sought to use our system to divide the bioproduction into three phases: a growth phase, a production phase and a further purification/extraction stage where products can be extracted from the culture media, separated from the cells after flocculation.

**Fig. 7:**
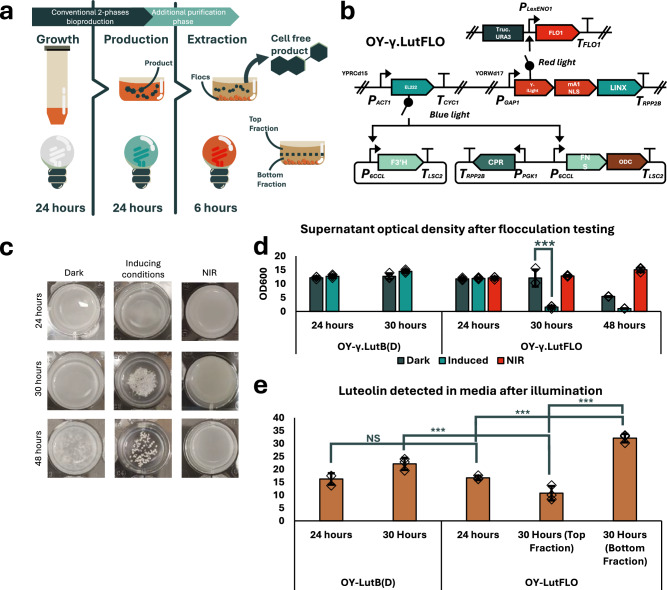
Light-controlled biomanufacturing and flocculation. **a** Workflow of growth-production-extraction three-phase biomanufacturing. **b** Genetic circuit of OY- γ.LutFlo. **c** Photographs of flocculation of OY- γ.LutFlo in various light conditions. Cultures were kept in either darkness for 48 h, 50% NIR(Near-Infrared) light for 48 h, or 24 h of 25% blue plus 50% NIR light, and 24 hours of 25% red light at 30 °C in a shaking incubator. **d** Optical density of supernatant during various phases of culturing. OY- γ.LutFlo and OY- γ.LutB(D) were cultured in either 30 h of darkness or 24 h of 25% blue plus 50% NIR light followed by 6 hours of 25% red light for OY- γ.LutFlo and 6 h of 25% blue plus 50% NIR light for OY- γ.LutB(D) at 30 °C in a shaking incubator. OY- γ.LutFlo was additionally cultured in 48 h of darkness, 48 h of 50% NIR light or 24 h of 25% blue plus 50% NIR light, followed by 24 h of 25% red light. OD_600_ measurements of the supernatant were taken at each of these timepoints. Values are an average of 3 biological replicates. Error bars indicate standard deviation with a sample size of *n* = 3 centred on the mean, results compared with 2-tailed independent *t*-test, NS = *p* > 0.1, * = *p* < 0.1, ** = *p* < 0.05, *** = *p* < 0.01. **e** Characterization of luteolin production during various phases of culturing. OY- γ.LutB(D) was cultured in 25% blue plus 50% NIR light for 30 hours at 30 °C in a shaking incubator, and flavonoid concentrations were measured at the 24 and 30 h mark. OY- γ.LutFlo was cultured in 25% blue plus 50% NIR light for 24 h followed by 6 hours in 25% red light at 30 °C in a shaking incubator. Flavonoids were extracted from the cell culture at 24 h, while at 30 h flavonoids were extracted from the supernatant (Top Fraction) or the remaining culture containing the flocs (Bottom Fraction). Values are an average of 3 biological replicates. Error bars indicate standard deviation with a sample size of *n* = 3 centred on the mean, results compared with 2-tailed independent *t*-test, NS = *p* > 0.1, * = *p* < 0.1, ** = *p* < 0.05, *** = *p* < 0.01. p-values for the labelled comparisons are as follows: (i)−0.00421, (ii)− 0.777, (iii)−0.00562, (iv)−0.00378,(v)−0.000331. Source data for this figure is available in the Source Data file.

Flocculation refers to the phenomenon where wildtype *S. cerevisiae* single cells aggregate in solution in response to stress conditions^88^, forming dense ‘flocs’ which sink in media. This can be applied to biomanufacturing as a method to separate out cells in solution, leaving the media as a supernatant and simplifying the purification and extraction process^89^ (Fig. 7a). Here, we chose to investigate the gene *FLO1*, whose overexpression can instigate flocculation in *S. cerevisiae*^90^. While optogenetic control of *FLO1* has been previously demonstrated^13^, it has yet to be paired with red light control or used in conjunction with the biomanufacturing of any product.

To facilitate a proof-of-concept involving the utility of red light-controlled flocculation, we decided to build on the optogenetic production of luteolin previously demonstrated. To assign the red light channel solely to flocculation, the production pathway for luteolin was confined to the blue light channel. A strain of OY- β.mA1LINX was reconstructed with F3’H and FNSI both as outputs of the blue light channel, but additionally had a *P*_*lexENO1*_ promoter inserted upstream of genomic *FLO1*, conferring the ability to flocculate in red light and was termed OY- γ.LutFlo (Luteolin-Flocculation) (Fig. 7b and Supplementary Fig. 13). A strain without the *FLO1* promoter replacement, OY- γ.LutB(D) was used as a negative control.

OY- γ.LutFlo and OY- γ.LutB(D) were cultured either in darkness or in 24 h of blue plus NIR light for luteolin production, followed by 24 h of red light for flocculation, and the OD_600_ of their suspension culture, as well as flavonoid profiles, were measured.

After 24 h of blue plus NIR light, OY- γ.LutB(D) and OY- γ.LutFlo had produced similar amount of luteolin (16.3 μM and 16.7 μM respectively) (Fig. 7e) and there was no statistical difference between OD_600_ for with cultures kept in the dark kept in the dark (Fig. 7d, e). After an additional 6 h in red light, OY- γ.LutFlo had clearly formed flocs that sank to the bottom of the cultures (Fig. 6b), while OY- γ.LutB(D) and OY- γ.LutFlo cultures that were grown in darkness remained in suspension.

At 30 hours, while the amount of luteolin produced by OY- γ.LutB(D) was slightly higher than what was measured at 24 h (22.1 μM), the supernatant of the OY- γ.LutFlo culture (Top Fraction) yielded less luteolin (10.7 μM) than was measured for cultures induced for 24 h (Fig. 6d). It was observed that the apigenin and eriodictyol in solution were also lower than expected (Supplementary Fig. 14). We hypothesized that the luteolin did not readily diffuse across the cell membrane and was still trapped in the flocs. Thus, 1 mL of media (volume kept at 1 mL to allow for comparison) containing the flocs underwent extraction and analysis (Bottom Fraction). The bottom fraction of OY- γ.LutFlo was observed to have significantly higher concentrations of luteolin (32.1 μM) than the top fraction (Fig. 7e), and also had a significantly higher concentration of luteolin than the control strain at 30 h (Fig. 7e), indicating that luteolin was not evenly distributed throughout the flocculated culture and was concentrated into the flocs.

It was also noted that leaving OY- γ.LutFlo cultures that had been growing 48 hours had significant flocculation and loss of suspension OD_600_ without any induction (Fig. 7c, d). However, it was found that by culturing cells in NIR light only, this unwanted flocculation could be avoided (Fig. 7c, d), indicating that it was due to spontaneous activation of the y-iLight transcription factor in darkness. Overall, it was shown that it was possible to use dual channel optogenetics to not only control gene pathways in a linear and quantitative manner, but also to qualitatively separate phases with different functions across time.

### Applying dual-channel optogenetics to biomaterial patterning

The final application of dual-channel optogenetics we covered in this study was leveraging the spatial resolution of optogenetics. The ability to use light patterns to specify the activation of genes in cells at specific locations has been of great use to biomaterials^4,91^, neuroscience^92^ and the study of communal interactions in microbes^41^. A common method of demonstrating the spatial properties of optogenetics is to project an image onto a lawn of cells bearing coloured or fluorescent gene products under light sensitive promoters, thus ‘printing’ the image onto the cells^12,74,93^. While images of multiple colours have been achieved in *E. coli*^18^, only mono-coloured images have been achieved with single channel optogenetics in *S. cerevisiae*^74^.

**Fig. 8:**
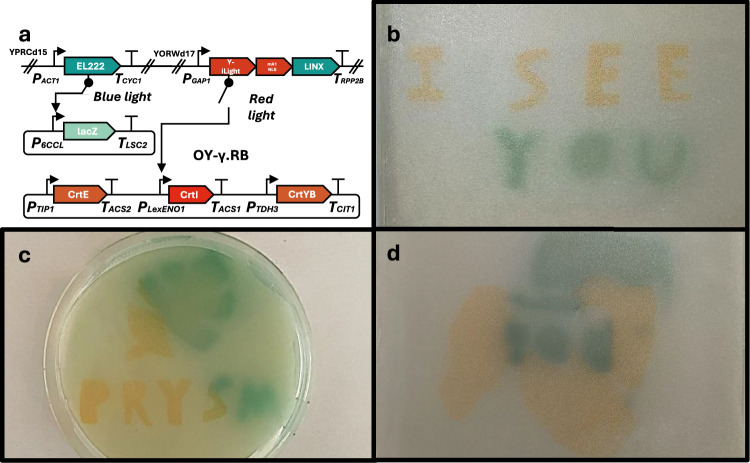
Dual coloured image printing on biofilms using optogenetics. Cells were deposited on YNB agar by evaporating a layer of cells suspended in water overnight. Plates were exposed to 5% red light for 24 h through a mask, followed by 100% blue light for 24 hours through a mask. **a** Genetic circuit for OY- γ.RB (Red-Blue). **b** Imprinting letters spelling ‘I SEE YOU’. **c** Imprint of the NUS iGEM 2021 logo, PRYSM. **d** Imprint of a still of the character Augie Steenback, from the film ‘Asteroid city’(Directed by Wes Anderson, 2023).

To achieve multi-coloured images, the strain OY- γ.RB (Red-Blue) was constructed from OY- β.mA1LINX. The lacZ enzyme was designated as the output of the blue light channel, and CrtI was designated as the output of the red light channel. In the presence of blue light, X-Gal would be catalysed to a blue dye(5,5’-dibromo-4,4’-dichloro-indigo), and in the presence of red light, CrtI would complete the biosynthesis of orange coloured beta-carotene (Fig. 8a and Supplementary Fig. 15).

OY- γ.RB was grown as a thin layer of cells on agar, and different images were projected onto the surface using our Optoboxes (Supplementary Fig. 16). Patterns were cut into black paper and used as a filter to illuminate select areas with red light for 24 hours, and blue light for 24 hours. It was found that in this case, NIR light was not necessary to remove crosstalk (Supplementary Fig. 17). Using this method, we were able to imprint several multi-colour patterns ranging from letters to logos and detailed images, demonstrating the high spatial resolution and potential of our system to develop complex living materials.

## Discussion

### Development of a single component red light optogenetic system for *S. cerevisiae*

We have refactored an optogenetic system that responded to red light stimulation for *S. cerevisiae*, requiring only a single transcription factor and independent of any exogenously added cofactors. This addressed the lack of compact single component red light optogenetic for *S. cerevisiae*, enabling the multiplexing applications of optogenetics in *S. cerevisiae*. In comparison, the most recently developed red light responsive transcription factor for *S. cerevisiae*, PhiReX, required expression of four different genes^10^ including genes for the biosynthesis of a cofactor not endogenous to *S. cerevisiae*. Using their PhiReX 1.1 were able to achieve up to 41-fold induction with levels similar to the native *S. cerevisiae* promoter *P*_*ADH1*_, and with PhiReX 1.0 they were able to achieve up to about 2x the strength of *P*_*ADH1*_, albeit with only 11-fold induction. While y-iLight has an induction fold of 26-fold, it has comparable activity to another native *S.cerevisiae* promoter *P*_*ACT1*_. Thus, the y-iLight system has a dynamic range in-between that of PhiRex 1.0 and 1.1, while similarly being on par with a commonly used native promoter.

An unexpected interaction that hindered the activity of dual channel optogenetics was the presence of crosstalk between blue light and the red light channel. Biliverdin, the light-sensitive cofactor of y-iLight, absorbs light in the red range of the visible spectrum in its ground state, but also has a Soret band in its absorbance spectrum that absorbs in the 400–450 nm range^94^. While the peak is not as high as the peak in the red light range, it is possible that it absorbs enough blue light to induce conformational change. The iLight system that was developed in mammalian cells did not exhibit any crosstalk under the conditions tested in that paper, but this may stem from their use of shorter, second-long light pulses as well as testing with a blue-green light of 505 nm wavelength instead of a pure blue 465 nm wavelength^49^. In addition, the amount of biliverdin present differs greatly from mammalian to *S.cerevisiae* cells, as mammalian cells break down biliverdin through biliverdin reductase^52^ while *S.cerevisiae* lacks this enzyme^51^. This may cause an increased build-up in biliverdin in *S.cerevisiae*, increasing its sensitivity to light.

To address this issue, we used a modular protein engineering approach different from the more commonly used directed evolution^95^ or rational protein design^96–98^. Our approach relied on the additive properties of protein subunits akin to parts, combining domains to achieve our desired effect. This represents a faster and less resource intensive method of leveraging previously established domains to generate different protein functions. Using this modular engineering method, we studied a number of combinatorial variants and we successfully created what amounts to single protein logic gates, as y-iLight variants produced gene expression outputs in response to the inputs ‘red light’ AND NOT ‘blue light’. This contrasts with the typical genetic circuits implemented as a network of transcriptional units^99,100^. In this study, we showed that it is possible to streamline the various components into one singular unit while keeping their functions. According to previous literature^101–103^, streamlining the different components of circuits to singular transcriptional units reduces the number of genes the cell needs to express and the number of interactions to be processed, which would allow for more complex logic to be implemented with less metabolic burden on the cell and less turnaround time from input to output. To our knowledge, this is also the first time crosstalk issues with light conditions relevant to other *S.cerevisiae* transcriptional optogenetic tools have been studied, and remedied with such modular protein engineering.

### Successful implementation of dual channel optogenetics

Using the variants of y-iLight, four strains of *S. cerevisiae* with dual-channel optogenetics were constructed. While OY- β.mA1LINX was eventually chosen for further studies, each of the dual-channel strains could prove useful under different circumstances. OY-α, exhibiting the highest expression, could be utilized in applications where high protein expression is required, and crosstalk is not an issue. OY- β.LINX requires the PSD to be cloned to the downstream gene of interest in the red light channel, but this could be an advantage when the downstream proteins need to be rapidly depleted, as the PSD is a translational level switch and is faster than relying on the natural half-life of proteins. OY- β.mA6LEXY is the only dual-channel strain that does not need additional supplementation by NIR light to eliminate crosstalk. It thus may be viable where tight expression is a priority or in scenarios where the use of two separate blue and NIR light sources is infeasible.

### Applications of dual-channel optogenetics

Several applications related to metabolic engineering, bioproduction and living materials were demonstrated. We were able to demonstrate that optogenetics can be used as a tunable and dynamic metabolic switch, to divert substrates between two different pathways with independent control, as well as to generate stepwise and time-resolved data that would be difficult to achieve with constitutive promoters or chemically inducible promoters. This system could be applied to better understand or model other complicated pathways with multiple points of control.

It was observed that luteolin produced by OY- γ.LutFLO did not completely reside in the media, with a disproportionate amount sequestered in the resultant flocs. This could be because flavonoids with multiple hydroxyl groups may have difficulty penetrating the hydrophobic cell membrane core^104^. Thus, this mode of biomanufacturing and purification may be better served with recombinant proteins that can be actively exported out of the cell using a secretion tag^105,106^, or chemical products that can be engineered for active export out of the cell^107^.

Alternatively, instead of purifying products from supernatant, it may be possible to use flocculation as a concentrating process. Products that do not actively cross the cell membrane, such as proteins without secretion tags, terpenes^107,108^ and vitamins^109^ could be manufactured in cells, and when flocculation is induced, the products are sequestered in the flocs. Because the volume of the dense flocs is far less than the total volume of the reaction, the waste media can be removed or even recycled, and flocs can be resuspended in a minimal volume to undergo lysis and purification. This would result in a far more concentrated product and may assist in purification and eliminate the need to concentrate chemicals artificially to relevant levels. This is supported by our data which demonstrated a higher concentration of luteolin was derived from flocs.

In conclusion, we have successfully implemented both red light and blue light sensitive optogenetics into a single strain of the industrially relevant yeast *S. cerevisiae*. A red light-sensitive transcription factor was optimized for use in *S.cerevisiae*. Using a modular protein engineering approach and optimized lighting condition (additional NIR light), we successfully eliminated unwanted blue light activation of the red light system. Nonetheless, additional optimization is warranted to further increase the activity of the system.

We demonstrated the utility of the dual channel system in controlling flux across metabolic pathways, using the luteolin production pathway as a test case, and showed shifting the proportion of each intermediate linearly with the proportion of light exposure. The potential to control distinct phases in cell culture was also demonstrated, using blue light to control luteolin production and red light to induce flocculation of cells.

We also demonstrated the ability to create patterns using X-gal and beta-carotene as coloured downstream products of each channel, which may prove useful in creating living biomaterials using *S. cerevisiae*.

Ultimately, this work serves as the basis for more complicated optogenetic circuits to be explored in *S.cerevisiae*, as the downstream genes of our dual channel system can be easily swapped out for other genes that may confer different functions. Additionally, we envision the methods used to optimize optogenetic circuits using modular engineering and creating single protein logic gates may prove useful in the design and implementation of increasingly complex functions in synthetic biology.

## Methods

### Optobox design and construction

To allow for the illumination of multiple wavelengths of light per sample, a custom-built programmable Optobox device was designed and 3D printed in PLA (design illustrated in Supplementary materials Supplementary Note 15, 3D STL files of Optobox provided in Supplementary Materials). The Optobox, when coupled with a standard 12-well plate, allows for the illumination of each sample with 4 independent wavelengths – namely Red (655 nm), Green (520 nm), Blue (468 nm) and NIR (770 nm) using 48 5 mm LEDs (refer to Supplementary Table 5). The entire Optobox is powered through a single 12 V DC jack and is controlled by an Arduino Uno R4 Minima microcontroller. To control the LEDs, a TLC5947-based Adafruit 24-Channel 12-bit PWM LED Driver breakout board (Adafruit, 1429) was used for modularity and to enable easy replacement of components. Each output channel is connected to 3 LEDs of the same colour in series for each column of the 12-well plate format, with the entire chain of LEDs equally driven at 20 mA. JST SM connectors were used between the LED Driver breakout board and LEDs to allow for detachability while still maintaining robustness. The LEDs are friction-fitted and secured using a non-frosting Cyanoacrylate glue (Bob Smith Industries, SUPER-GOLD + ) onto the LED Holder, which forms the lid of the Optobox. The LED Holder also helps to set the spacing between each LED and the 12-well plate, while also diffusing and isolating the light from each individual well.

The entire Optobox is cooled with a single 4040 sized 12 V DC fan (SUNON, MF40101VX-1000U-A99) blowing longitudinally across the Optobox. The circuit diagram is illustrated in Supplementary Fig. 19. The Arduino codes are provided in Supplementary Materials.

Light intensities (Supplementary Fig. 20) were measured using a Coherent LaserCheck power meter (Edmund Optics, 1098293). For a detailed description of the Optobox design considerations and design features, refer to Supplementary Note 15.

### Strains and growth conditions

All yeast strains were derived from *S. cerevisiae* BY4741 grown in either YPD (1% yeast extract, 2% peptone, 2% dextrose) or YNB minimal media with appropriate drop in amino acids and nutrients to maintain auxotrophic selection pressures. For hygromycin selection, 300 μg/ml of hygromycin B was used for agar selection, while 100 μg/ml was used for liquid media cultures. Cultures were kept at 30 °C, 190 rpm shaking in thermoregulated incubators. Lithium acetate method was used for yeast transformations, and transformants were selected for on appropriate 2% agar medium. For integrative plasmids, isolated plasmids were digested with their respective restriction enzymes for 2 hours at 37 °C to linearize the integrative cassette, and the resultant reaction transformed into *S. cerevisiae*.

### Yeast transformation

Yeast cells were grown to stationary phase overnight, and 200 μL of cells were added to 5 mL of selective media and grown at 30 °C, 190 rpm shaking for 3 h. Cells were spun down, and the pellet was washed twice in 1 mL of water. Pellet was resuspended in 36 μL of 1 M lithium acetate, 10 μL of boiled 10 mg/mL sheared salmon sperm, 100 ng of DNA and topped up to 120 μL in volume with DI water. 240 μL of 50% PEG3350 was added and mixed by pipetting. Mixture was incubated at 42 °C for 30 min, spun down and resuspended in 1 mL of water. 100 μL of water was spread on selective agar.

### Genes and plasmid cloning

All plasmids (Supplementary Table 1) were constructed using NEB HiFi Gibson Assembly. NEB 10beta *E. coli* were used in cloning, and were cultured at 37 °C, 190 rpm shaking in thermoregulated incubators. Full sequences for all genes used in this study can be found in Supplementary Data 1, and all plasmids with annotated sequences can be found in the source data file.

BY474B: Expression cassette for *P*_*ADH1*_ promoter-EL222-*T*_*ADH1*_ was synthesized by IDT, and cloned into the pGAH-YPRCd15 integration vector^110^, which was linearized with BsaI. *P*_*6CCL*_ was synthesized with IDT (Integrated DNA technologies, USA) and cloned upstream of mTurquoise-*T*_*LSC2*_ in a pESC-Leu episomal vector.

BY474R: iLight IsPad PCM was obtained from pCMVd1-NLS-GAL4-iLight-VP16-T2A-mTagBFP2, which was a gift from Vladislav Verkhusha (Addgene plasmid # 170273; http://n2t.net/addgene:170273; RRID:Addgene_170273)^49^. Wildtype LexA DNA binding domain was obtained through PCR from NEB 10beta *E.coli*. LexA and iLight were cloned into pGAL-YORWd17^110^, and SV40 NLS was re-appended using PCR to produce the y-iLight ORF. Leu2 marker was replaced with Hph hygromycin B resistance marker, and BsaI restriction sites were replaced with NruI sites to avoid clashing with internal restriction sites. Plasmid was linearized with NruI. Note that the number of transformants is greatly decreased for hygromycin B selection. Eight *lexO* repeats upstream of *cP*_*CYC1*_ core promoter, mCitrine ORF and *T*_*CYC1*_ were cloned from FRP795_insul-(lexA-box)8-PminCYC1-Citrine-TCYC1, which was a gift from Joerg Stelling (Addgene plasmid # 58435; http://n2t.net/addgene:58435; RRID:Addgene_58435)^54^ into pESC_URA vector. Core promoters *cP*_*ENO1*_ and *cP*_*PGK1*_ were amplified from *S.cerevisiae* isolated genome.

iLight variants: PSD, LINXa3 and LEXY were synthesized by IDT, and NLS were added with PCR primers. These were cloned into the y-iLight ORF at the desired junctions.

Dual channel strains: For all dual channel strains, y-iLight and EL222 integrative plasmids were transformed, followed by their respective episomal reporter plasmids.

Luteolin Producing strains: Protein sequence for *F3’H* from *Petunia x* hybrida (Accession number Q9SBQ9.1) and sequence for *FNSI* from *Petroselinum crispum* (Accession number Q7XZQ8.1) were optimized for *S. cerevisiae* and synthesized by IDT. Ornithine Decarboxylase degron was PCR amplified from PSD module and cloned downstream of *FNSI*. *F3’H* was cloned downstream of *P*_*6CCL*_ in pESC_Leu. *FNSI* was either cloned downstream of *P*_*lexENO1*_ or *P*_*6CCL*_ in pESC_Ura. These were cloned into BY4741 integrated with both EL222 expression and y-iLightmA1LINX expression to construct OY- γ.LutB/R(D) and γ.LutB(D).

Flocculating strains: URA marker and homologous region for counter-selection was PCR amplified from pESC_URA and fused upstream of *P*_*lexENO1*_ through overlap PCR. The homologous ends for *FLO1* promoter replacement were obtained from Salinas et al., (2018)^13^. Counterselection was carried out using 100ug/mL 5’FOA, in YNB agar with 50% uracil.

Optogenetic imaging strain: LacZ was PCR amplified from the BL21 (DE3) genome and cloned downstream of *P*_*6CCL*_ in pESC_Leu. pLM494 was a gift from Bernd Müller-Röber (Addgene plasmid # 100539; http://n2t.net/addgene:100539; RRID:Addgene_100539)^44^, and promoter for *CRTI* gene was replaced with *P*_*lexENO1*_. Both these plasmids were cloned into BY4741 integrated with both EL222 expression and y-iLightmA1LINX expression to construct OY- γ.R/B.

### Optogenetic experiments

For cultivation on optoboxes, all strains to be tested were inoculated from glycerol stocks over two nights to reach late stationary phase. OD_600_ of cultures were measured and diluted in appropriate selective media to OD_600_ = 1.5. Biological replicates were separate instances of strains inoculated from glycerol stocks. Three technical replicates of 2 mL of diluted culture were aliquoted into 12-well plates per biological replicate, and the 12-well plates were sealed with parafilm to prevent excessive evaporation. LEDs in optoboxes were set to the desired colour and intensity, and secured in a tabletop incubator set to 30 °C, 190 rpm. For this paper, we set 100% of blue light to be 16.94 mW/cm^2^ (PMW = 3072), 100% of red light to be 10.31 mW/cm^2^ (PMW = 4095) and 100% of NIR light to be 9.25 mW/cm^2^ (PMW = 4095). The 12-well plates were secured to optoboxes and were covered with a lid folded from black paper to prevent interference from external light sources.

Fluorescent and OD_600_ readings of 12-well plates were taken using a BioTek H1 synergy plate reader (Agilent, USA), mCitrine was measured at an excitation wavelength of 480 nm and an emission wavelength of 530 nm^111^, and mTurquoise was measured at an excitation wavelength of 434 nm and an emission wavelength of 474 nm^112^. Readings were blanked against media, and fluorescent readings were normalized to OD_600_ to obtain the relative fluorescent units (RFU).

### Flavonoid analysis

To analyze the profile of flavonoids produced from naringenin by strains, bulk YNB media was prepared in a 50 mL falcon tube and 75 μM naringenin was mixed inside by inverting the tube several times. This media was used for 12-well 2 mL scale cultures, induced by the same optogenetic culturing conditions as stated above for 24 h. Technical triplicates of 2 mL cultures were used per biological replicate, biological replicates were separate instances of strains inoculated from glycerol stocks. Cell cultures were mixed with vigorous pipetting, and 1 mL drawn and mixed with 1 mL of pure ethanol to lyse cells and solubilize flavonoids. This mixture was then filtered using a Acrodisc 25 mm, 0.22 um pore size sterile filters (PALL, USA). Filtrate was analyzed with a Chromemaster High Performance Liquid Chromatography (HPLC) (Hitachi, Japan) equipped with a Hitachi C18 column(4.5 mmI.D × 150 mmL, 5 μM) with an injection volume of 10 μL, column temperature of 40 °C, and a flow rate of 1.5 mL/minute for 35 min. Mobile phase of HPLC consists of 40% methanol and 6% acetic acid solution dissolved in water, with signals for all compounds detected at 300 nm. Elution times for chemicals were ~8 minutes for eriodictyol, ~14 min for naringenin, ~18 min for luteolin and ~29 minutes for apigenin. Standard curves for all compounds were prepared by diluting compound in 2 mL of YNB and undergoing the same ethanol dilution and filtering as cell cultures (Supplementary Fig. 10).

### RT-qPCR

NEB Luna Universal One Step RT-PCR kit was used to carry out RT-qPCR of both F3’H and FNSI genes in OY- γ.LutB/R(D). OY- γ.LutB/R(D) was inoculated from glycerol stocks over two nights to reach late stationary phase. OD_600_ of cultures were measured and diluted in appropriate selective media to OD_600_ = 1.5. Biological replicates were separate instances of strains inoculated from glycerol stocks. OY- γ.LutB/R(D) was cultured in selective media for 5 hours in respective light cycling conditions at 30 °C, shaking, and RNA was extracted with Zymo research RNA isolation kit. Primers for F3’H and FNSI amplification were designed using the IDT PrimerQuest primer design tool (Supplementary Note 7 and Supplementary Table 2). Fluorescence was captured after PCR cycling with two technical replicates per isolated RNA. UBC6 was used as a reference gene, with primers obtained from Teste et al. ^113^ ΔCt between FNI/F3’H and UBC6 was compared to OY- γ.LutB/R(D) was kept in darkness to derive the ΔΔCt.

### Computational kinetics profiling

Leveraging experimental data collected upon exposed to varying light duty cycles, we developed an in silico mathematical model to quantitatively dissect the differential flux between the two pathways. The model explicitly accounts for the transcriptional and translational dynamics of the two enzymes regulated by either blue light or red light, and the reaction kinetics governing the flavonoid conversion. These processes were described using a system of ordinary differential equations (ODEs) to encode both regulatory and catalytic processes. Implementation was performed in Python 3, with numerical solution computed by solve_ivp function from the SciPy library. The initial naringenin concentrations were defined by the sum of total flavonoid concentration measured at the individual illumination conditions. Model parameters were estimated using Nelder-Mead optimization upon fitting to the experimental data. Comprehensive details of the model formulations and the estimated kinetic parameters are provided in Supplementary Tables 3-4.

### Flocculation characterization

For flocculating cultures, images were taken using an OPPO Reno7 Pro 5 G cellphone. To measure the OD_600_ of the suspension, cells were removed from the shaking incubator and left to stand for 2 minutes, and 100 μL of solution from the surface of the culture was drawn and diluted by 10x before OD_600_ was measured. For measurements indicating a top and bottom fraction, the top fraction was drawn after 2 min of standing without mixing, to prevent the flocs from being disturbed. This was then diluted into 1 mL of ethanol as per the flavonoid characterization protocol. The bottom fraction was obtained by dispensing 1 mL of ethanol directly into the remaining culture with the flocs present, and mixing by pipetting vigorously to lyse cells. Both fractions were filtered with Acrodisc 25 mm, 0.22 μm pore size sterile filters (PALL, USA) before HPLC analysis. Per biological replicate, three 2 mL technical replicates were cultured, biological replicates were separate instances of strains inoculated from glycerol stocks.

### Image printing with Optobox

To utilize optogenetics to imprint images onto a layer of cells, OY- γ.RB was first cultured from glycerol stocks over two nights to reach late stationary phase. 40 mL of YNB agar without amino acids with 200 μg/mL of X-Gal added was filled into a Nunc square assay dish, and left to dry at 30 °C. 200 μL of culture was mixed with 3 mL of sterilized DI water, and spread evenly across the agar after it had dried, and was left at the same spot at 30 °C overnight. By the next day, the diluted culture would have dried up, leaving a layer of cells across the agar surface. Leaving cell culture to dry in the same spot as the agar hardened ensures that the diluted culture is level with the agar and thus would form an even layer. The plate is then placed in the appropriate light source with the appropriate mask, with the agar facing the light source, for 24 h at 30 °C. Plates are exposed to red light for 24 h first, and then blue light for 24 h. See Supplementary Note 9 for measurements for setup of image printing.

### Statistics and reproducibility

No statistical method was used to predetermine sample size. No data were excluded from the analyses. The experiments were not randomized. The investigators were not blinded to allocation during experiments and outcome assessment. A two-tailed t-test with equal variance was used to determine statistical significance for all results compared.

### Reporting summary

Further information on research design is available in the Nature Portfolio Reporting Summary linked to this article.

## Supplementary information

Supplementary Information

Description of Additional Supplementary Files

Supplementary Data 1

Reporting Summary

Transparent Peer Review file

## Source data

Source Data

## Data Availability

Source data are available with this manuscript, plasmid sequences and ORFs used in this study are provided in the Source Data files. Accession codes used in this study: Q9SBQ9.1, [https://www.ncbi.nlm.nih.gov/protein/Q9SBQ9], Protein sequence for F3’H from *Petunia x* hybrida Q7XZQ8.1, [https://www.ncbi.nlm.nih.gov/protein/Q7XZQ8.1], Protein sequence for FNSI from *Petroselinum crispum* Source data are provided with this paper.

## Code availability

The code files are provided in the Supplementary materials and are accessible on Zenodo (10.5281/zenodo.19547358)^114^ and Github (https://github.com/EngBioNUS/Dual_channel_optogenetics_kinetic_models).
