# Supplementary material for: Dual-channel optogenetics in yeast for multiplexed light-based control of cellular processes and pathways
Source: Nat Commun. 2026 May 22;17:6742. doi: 10.1038/s41467-026-73399-0 (PMC13385801; doi:10.1038/s41467-026-73399-0)
Supplement: Supplementary file 1 — Supplementary Information [file 41467_2026_73399_MOESM1_ESM.pdf]

Title: Dual-channel optogenetics in yeast for multiplexed light-based control of cellular processes and pathways

Supplementary Note 1: Gene parts and sequences

<u>Supplementary Table 1. List of plasmids used in this study</u>

Name	Description	Source
pGAL_YORWd17	Vector for genome integration into locus YORWd17	Wong, G. et al[1]
pCMVd1-NLS-GAL4-iLight-VP16-T2A-mTagBFP2	Plasmid expressing iLight optimized for mammalian cells	Kabernuik, A.A. et al[2]
py-iLight_Hyg_YORWd17	Genome integrated plasmid expressing y-iLight	This study
pESC_Ura	Uracil auxotrophy selection episomal plasmid	Agilent Technologies, USA
FRP795_insul-(lexA-box)8-PminCYC1-Citrine-TCYC1	Plasmid with array of *lexO* repeats	Ottoz, D. S. M. et al[3]
pLexCYC_mCitrine_Ura	Plasmid with $P_{lexCYC1}$ expressing mCitrine	This study
pLexENO_mCitrine_Ura	Plasmid with $P_{lexENO1}$ expressing mCitrine	This study
pLexPGK_mCitrine_Ura	Plasmid with $P_{lexPGK1}$expressing mCitrine	This study
pGAH_YPRCd15	Vector for genome integration into locus YPRCd15	Wong, G. et al[1]
pEL222_His_YPRCd15	Genome integrated plasmid expressing EL222	This study
pESC_Leu	Leucine auxotrophy selection episomal plasmid	Agilent Technologies, USA
p6CCL-mTurq_Leu	Plasmid with P_{6CCL} expressing mTurquoise	This study
pLexENO_mCitrinePSD_Ura	Plasmid with $P_{lexENO1}$ expressing mCitrine fused to PSD	This study
py-iLight(PSD)_Hyg_YORWd17	Genome integrated plasmid expressing y-iLight fused to PSD	This study
py-iLightLINX_Hyg_YORWd17	Genome integrated plasmid expressing y-iLight fused to LINX	This study
py-iLightLEXY_Hyg_YORWd17	Genome integrated plasmid expressing y-iLight fused to LEXY	This study
py-iLightmA1LINX_Hyg_YORWd17	Genome integrated plasmid expressing y-iLight with mA1 NLS fused to LINX	This study
py-iLightmA6LEXY_Hyg_YORWd17	Genome integrated plasmid expressing y-iLight with mA6 NLS fused to LEXY	This study
p6CCL-F3'H_Leu	Plasmid with P_{6CCL} expressing F3'H	This study
pLexENO-FNSI(ODC)_PGK_CPR_Ura	Plasmid with $P_{lexENO1}$ expressing FNSI fused to ODC and constitutive expression of scCPR	This study
p6CCL-FNSI(ODC)_PGK_CPR__Ura	Plasmid with P_{6CCL} expressing FNSI fused to ODC and constitutive expression of scCPR	This study
LexENO_knockin	Fragment for knock in of $P_{lexENO1}$ to replace *FLO1* promoter	This study
pLM494	Plasmid with constitutive expression of beta-carotene biosynthesis pathway	Hochrein, L. et al[4]
pLM494_LexENO-CrtI_Ura	Plasmid with constitutive expression of beta-carotene biosynthesis pathway, except *CrtI* expressed by $P_{lexENO1}$	This study
p6CCL-lacZ_Leu	Plasmid with P_{6CCL} expressing LacZ	This study

Supplementary Note 2: OD_{600} values of single channel induction experiment

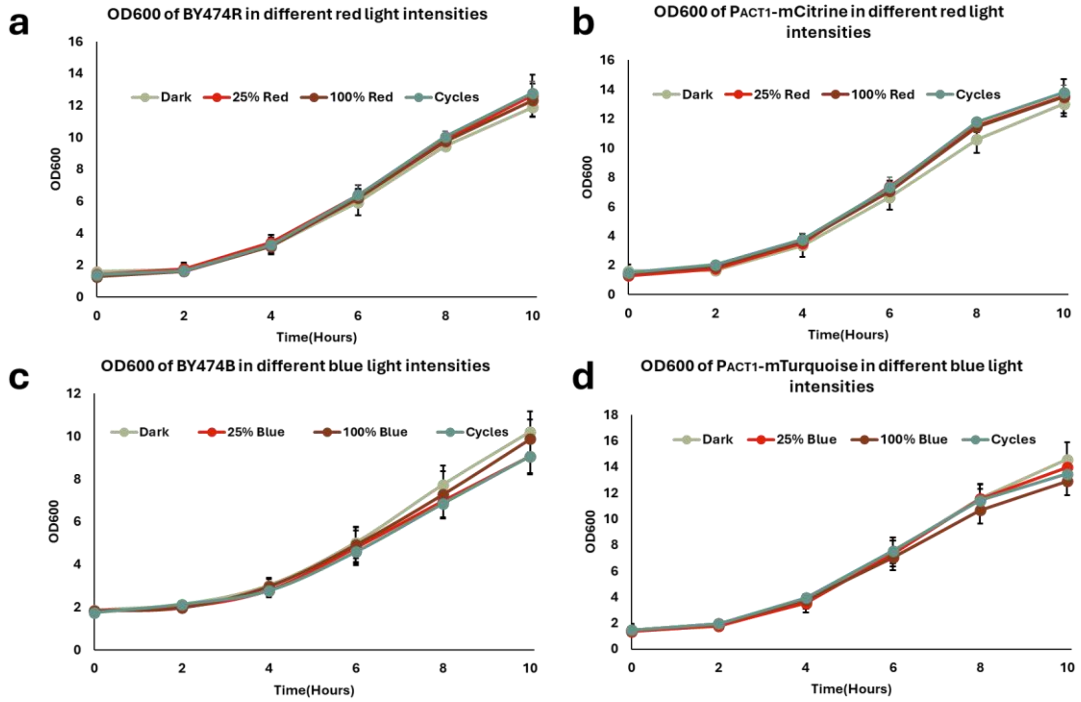

Supplementary Fig. 1: **a** OD_{600} values of strain BY474R across 10 hours after culturing in darkness, 25% red light, 100% red light and cycles of 30 minutes of 100% red light followed by 30 minutes of darkness. Values are an average of 3 biological replicates. Error bars indicate standard deviation with a sample size of n=3. **b** OD_{600} values of BY4741 expressing mCitrine reporter from native promoter P_{ACT1} across 10 hours after culturing in darkness, 25% red light, 100% red light and cycles of 30 minutes of 100% red light followed by 30 minutes of darkness. Values are an average of 3 biological replicates. Error bars indicate standard deviation with a sample size of n=3. **c** OD_{600} values of strain BY474B across 10 hours after culturing in darkness, 25% blue light, 100% blue light and cycles of 30 minutes of 100% blue light followed by 30 minutes of darkness. Values are an average of 3 biological replicates. Error bars indicate standard deviation with a sample size of n=3. **d** OD_{600} values of BY4741 expressing mTurquoise reporter from native promoter P_{ACT1} across 10 hours after culturing in darkness, 25% blue light, 100% blue light and cycles of 30 minutes of 100% blue light followed by 30 minutes of darkness. Values are an average of 3 biological replicates. Error bars indicate standard deviation with a sample size of n=3.

Experiments demonstrated that the expression of mCitrine reporter from BY474R decreased in high intensities of red light, and expression of mTurquoise reporter from native constitutive promoters decreased in high intensities of blue light (See Supplementary Note 16). In both cases, the decrease in expression could be alleviated by using 30-minute duty cycles of high intensity light. To examine if high intensity light caused growth defects in *S. cerevisiae* that resulted in the lowered expression, strains expressing either reporter from their optogenetic system or the native P_{ACT1} promoter were grown in darkness, 25% light, 100% light or 30-minute cycles of 100% light and darkness. OD_{600} was periodically measured, and if reduction in reporter protein was due to a phototoxic effect on growth, slower growth would be expected in higher intensities of light. We would also expect that slow growth

would be alleviated in cycles of high intensity light to correspond with the recovered expression of reporter protein. However, for all strains in all conditions, no reduction in growth was observed in lit conditions compared to darkness (Fig. S1a-d).

Supplementary Note 3: Exponential phase, stationary phase and late stationary phase data for optogenetic experiments

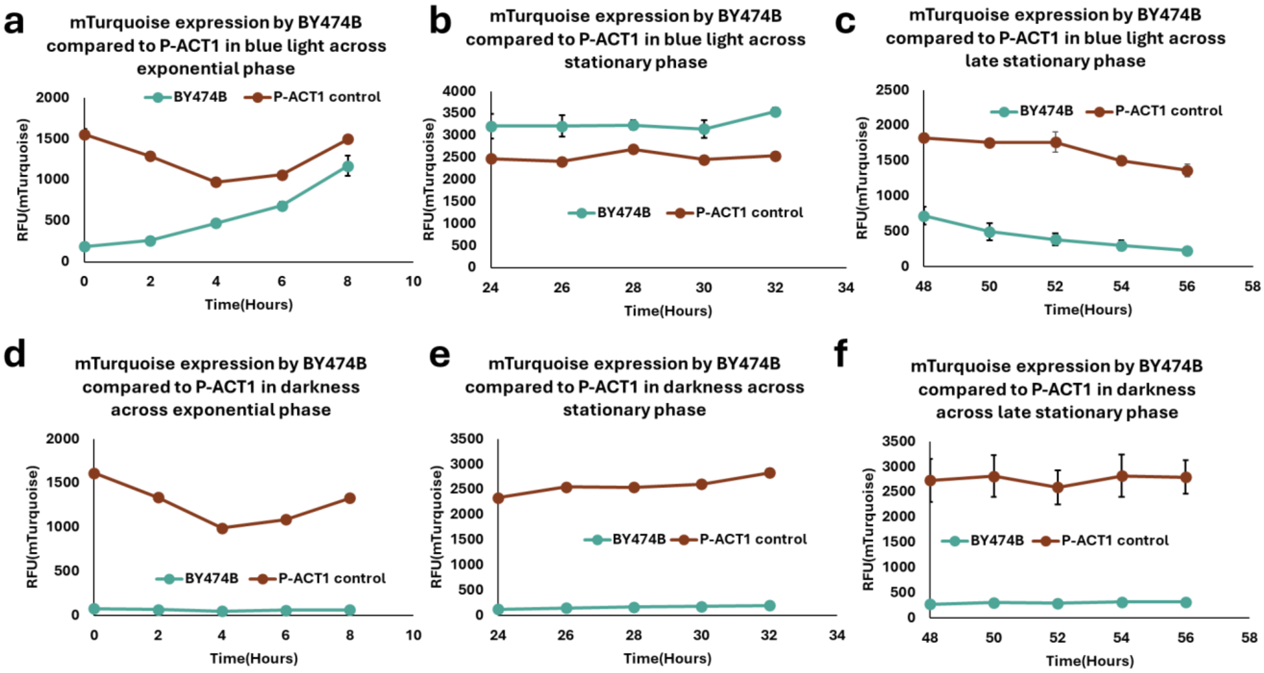

Supplementary Fig. 2: Activity of blue channel strain over exponential, stationary and late stationary phase. BY474B as well as strain expressing mTurquoise from P_{ACT1} were exposed to 25% blue light and cultured at 30°C in a shaking incubator for 56 hours. **a** To measure expression in the exponential phase, fluorescence measurements were taken every 2 hours and normalized to OD_{600} from 0 to 8 hours. **b** To measure expression in the stationary phase, fluorescence measurements were taken every 2 hours and normalized to OD_{600} from 24 to 32 hours. **c** To measure expression in the late stationary phase, fluorescence measurements were taken every 2 hours and normalized to OD_{600} from 48 to 56 hours. BY474B as well as strain expressing mTurquoise from P_{ACT1} were kept in darkness and cultured at 30°C in a shaking incubator for 56 hours. **d** To measure expression in the exponential phase, fluorescence measurements were taken every 2 hours and normalized to OD_{600} from 0 to 8 hours. **e** To measure expression in the stationary phase, fluorescence measurements were taken every 2 hours and normalized to OD_{600} from 24 to 32 hours. **f** To measure expression in the late stationary phase, fluorescence measurements were taken every 2 hours and normalized to OD_{600} from 48 to 56 hours. Values are an average of 3 biological replicates. Error bars indicate standard deviation with a sample size of n=3. Values are an average of 3 biological replicates. Error bars indicate standard deviation with a sample size of n=3. Note that to account for higher fluorescent levels, gain for fluorescent readings was lowered to 75% that of 24 hour experiments.

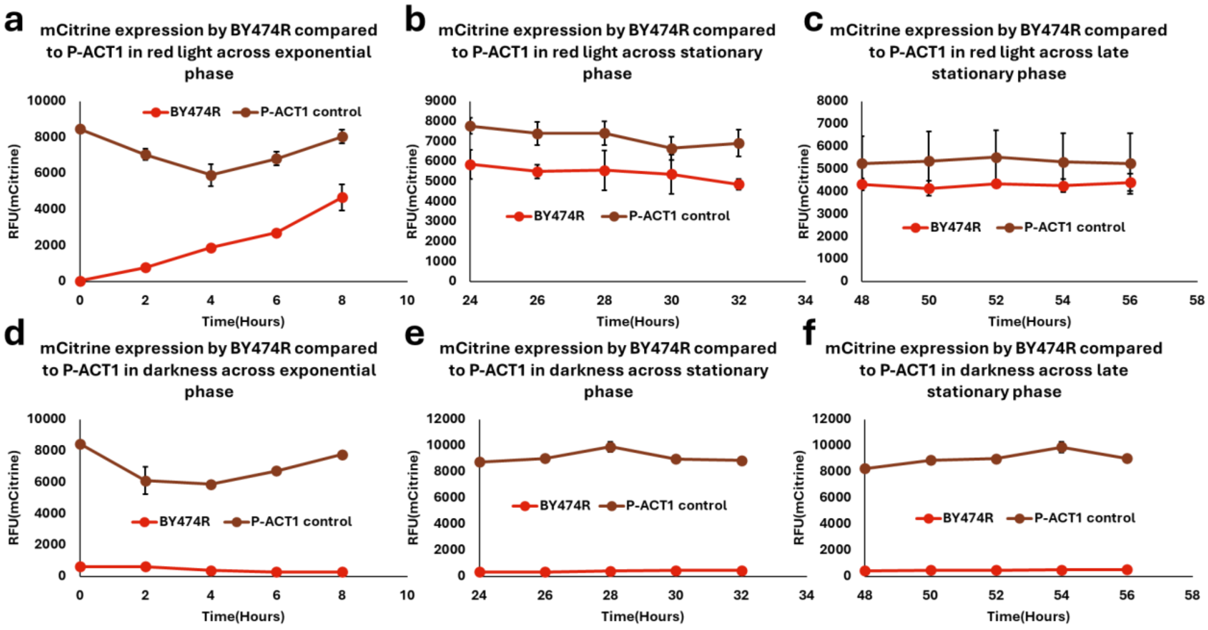

Supplementary Fig. 3: Activity of red channel strain over exponential, stationary and late stationary phase. BY474R as well as strain expressing mCitrine from P_{ACT1} were exposed to 25% red light and cultured at 30°C in a shaking incubator for 56 hours. **a** To measure expression in the exponential phase, fluorescence measurements were taken every 2 hours and normalized to OD_{600} from 0 to 8 hours. **b** To measure expression in the stationary phase, fluorescence measurements were taken every 2 hours and normalized to OD_{600} from 24 to 32 hours. **c** To measure expression in the late stationary phase, fluorescence measurements were taken every 2 hours and normalized to OD_{600} from 48 to 56 hours. BY474R as well as strain expressing mCitrine from P_{ACT1} were kept in darkness and cultured at 30°C in a shaking incubator for 56 hours. **d** To measure expression in the exponential phase, fluorescence measurements were taken every 2 hours and normalized to OD_{600} from 0 to 8 hours. **e** To measure expression in the stationary phase, fluorescence measurements were taken every 2 hours and normalized to OD_{600} from 24 to 32 hours. **f** To measure expression in the late stationary phase, fluorescence measurements were taken every 2 hours and normalized to OD_{600} from 48 to 56 hours. Values are an average of 3 biological replicates. Error bars indicate standard deviation with a sample size of n=3. Values are an average of 3 biological replicates. Error bars indicate standard deviation with a sample size of n=3. Note that to account for higher fluorescent levels, gain for fluorescent readings was lowered to 75% that of 24 hour experiments.

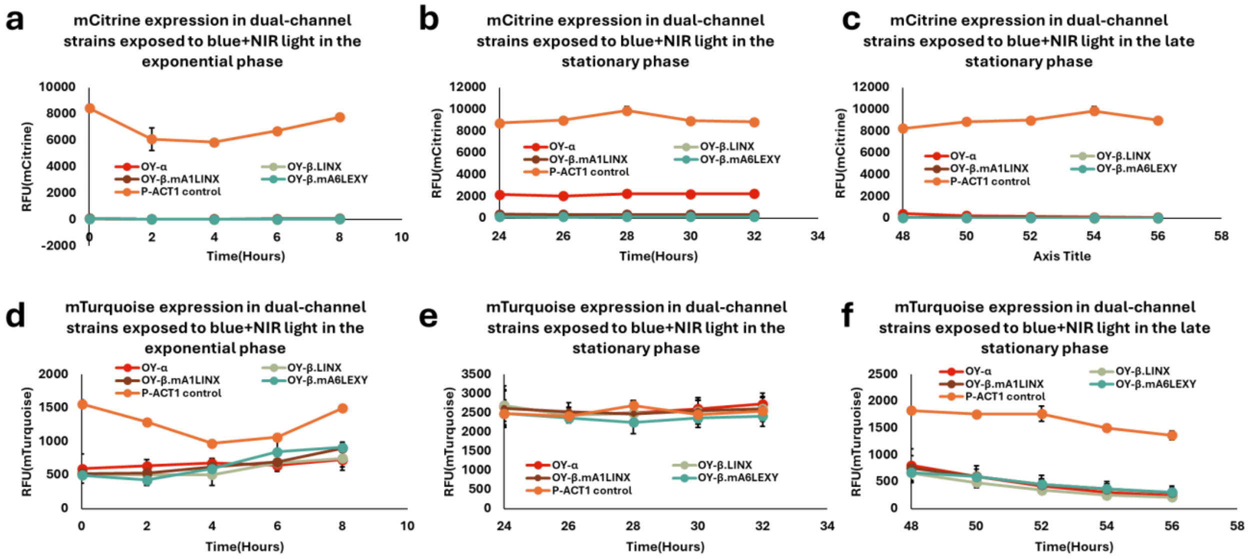

Supplementary Fig. 4: Activity of dual channel strains in blue plus NIR light over exponential, stationary and late stationary phase. OY-α, OY-β.LINX, OY- β.mA1LINX and OY- β.mA6LEXY were exposed to 25% blue plus 50% NIR light and cultured at 30ºC in a shaking incubator for 56 hours. To examine the activity of the red light channel, mCitrine fluorescence was measured and normalized to OD_{600} every 2 hours from 0 to 8 hours to measure expression in the exponential phase (**a**), 24 to 32 hours to measure expression in the stationary phase (**b**) and 48 to 56 hours to measure expression in the late stationary phase (**c**). These were compared to data of the P_{ACT1} promoter expressing mCitrine kept in darkness. To examine the activity of the blue light channel, mTurquoise fluorescence was measured and normalized to OD_{600} every 2 hours from 0 to 8 hours to measure expression in the exponential phase (**d**), 24 to 32 hours to measure expression in the stationary phase (**e**) and 48 to 56 hours to measure expression in the late stationary phase (**f**). These were compared to data of the P_{ACT1} promoter expressing mTurquoise in 25% blue light. Values are an average of 3 biological replicates. Error bars indicate standard deviation with a sample size of n=3. Values are an average of 3 biological replicates. Error bars indicate standard deviation with a sample size of n=3. Note that to account for higher fluorescent levels, gain for fluorescent readings was lowered to 75% that of 24 hour experiments.

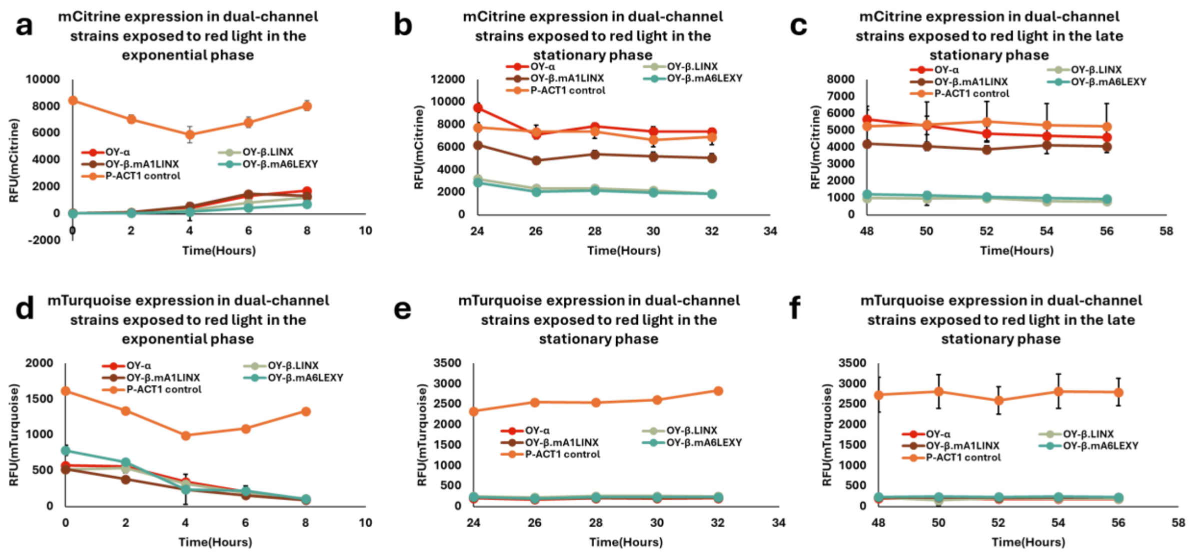

Supplementary Fig. 5: Activity of dual channel strains in red light over exponential, stationary and late stationary phase. OY-α, OY-β.LINX, OY- β.mA1LINX and OY-β.mA6LEXY were exposed to 25% red light and cultured at 30ºC in a shaking incubator for 56 hours. To examine the activity of the red light channel, mCitrine fluorescence was measured and normalized to OD_{600} every 2 hours from 0 to 8 hours to measure expression in the exponential phase (**a**), 24 to 32 hours to measure expression in the stationary phase (**b**) and 48 to 56 hours to measure expression in the late stationary phase (**c**). These were compared to data of the P_{ACT1} promoter expressing mCitrine in 25% red light. To examine the activity of the blue light channel, mTurquoise fluorescence was measured and normalized to OD_{600} every 2 hours from 0 to 8 hours to measure expression in the exponential phase (**d**), 24 to 32 hours to measure expression in the stationary phase (**e**) and 48 to 56 hours to measure expression in the late stationary phase (**f**). These were compared to data of the P_{ACT1} promoter expressing mTurquoise kept in darkness. Values are an average of 3 biological replicates. Error bars indicate standard deviation with a sample size of n=3. Values are an average of 3 biological replicates. Error bars indicate standard deviation with a sample size of n=3. Note that to account for higher fluorescent levels, gain for fluorescent readings was lowered to 75% that of 24 hour experiments.

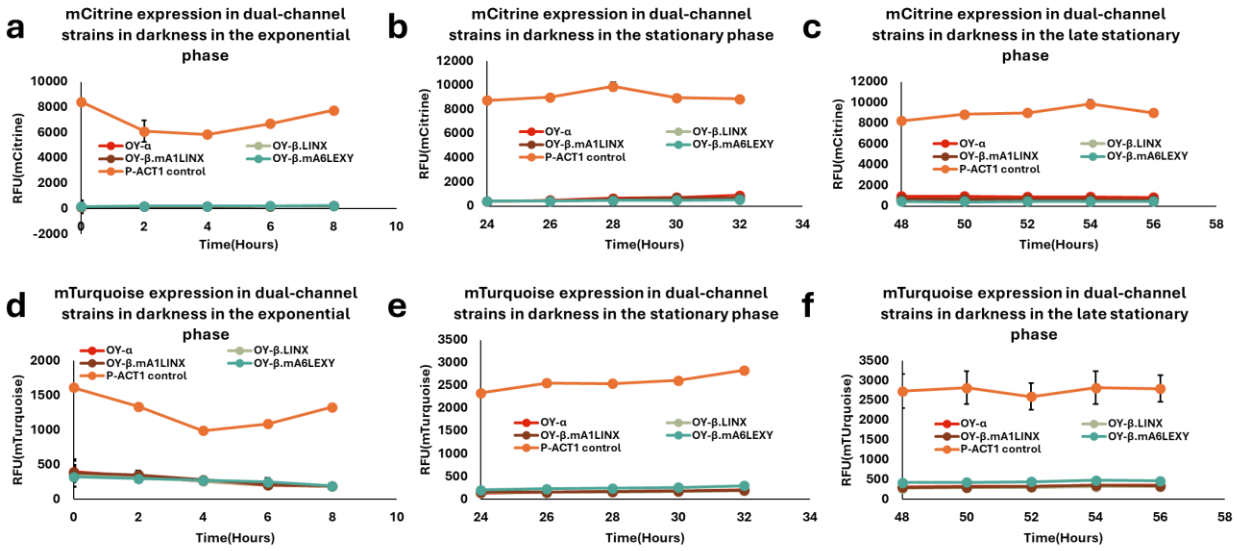

Supplementary Fig. 6: Activity of dual channel strains in darkness over exponential, stationary and late stationary phase. OY-α, OY-β.LINX, OY- β.mA1LINX and OY-β.mA6LEXY were kept in darkness and cultured at 30ºC in a shaking incubator for 56 hours. To examine the activity of the red light channel, mCitrine fluorescence was measured and normalized to OD_{600} every 2 hours from 0 to 8 hours to measure expression in the exponential phase (**a**), 24 to 32 hours to measure expression in the stationary phase (**b**) and 48 to 56 hours to measure expression in the late stationary phase (**c**). These were compared to data of the P_{ACT1} promoter expressing mCitrine kept in darkness. To examine the activity of the blue light channel, mTurquoise fluorescence was measured and normalized to OD_{600} every 2 hours from 0 to 8 hours to measure expression in the exponential phase (**d**), 24 to 32 hours to measure expression in the stationary phase (**e**) and 48 to 56 hours to measure expression in the late stationary phase (**f**). These were compared to data of the P_{ACT1} promoter expressing mTurquoise kept in darkness. Values are an average of 3 biological replicates. Error bars indicate standard deviation with a sample size of n=3. Values are an average of 3 biological replicates. Error bars indicate standard deviation with a sample size of n=3. Note that to account for higher fluorescent levels, gain for fluorescent readings was lowered to 75% that of 24 hour experiments.

Supplementary Note 4: Effect of reducing blue light intensity on y-iLight crosstalk

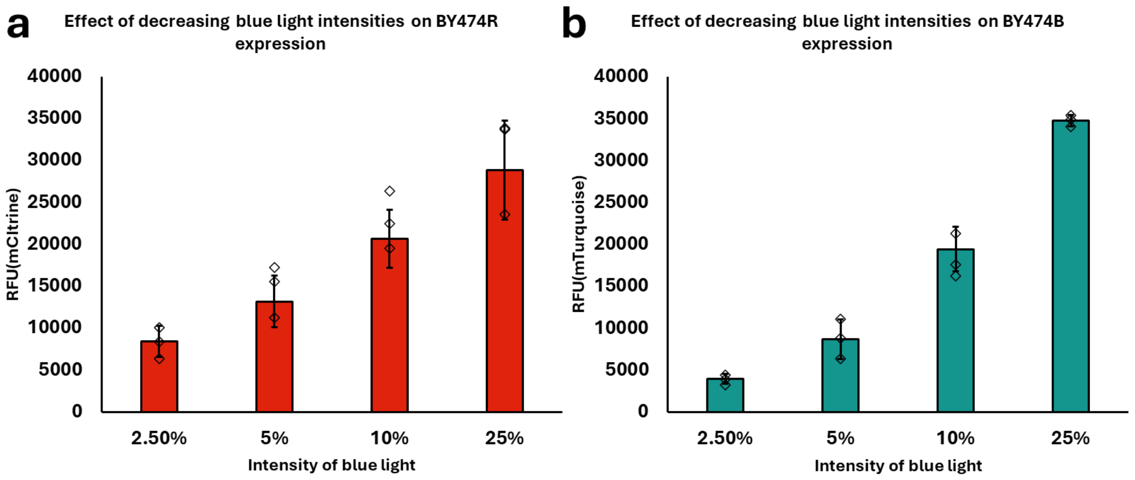

Supplementary Fig. 7: Effect of decreasing blue light intensities on BY474R and BY474B. a BY474R was cultured in 2.5%, 5%, 10% or 25% of blue light at 30ºC in a shaking incubator. At 24 hours, mCitrine fluorescence was measured and normalized to OD_{600}. **b** BY474B was cultured in 2.5%, 5%, 10% or 25% of blue light at 30ºC in a shaking incubator. At 24 hours, mCitrine fluorescence was measured and normalized to OD_{600}. Values are an average of 3 biological replicates. Error bars indicate standard deviation with a sample size of n=3. Values are an average of 3 biological replicates. Error bars indicate standard deviation with a sample size of n=3.

As an initial effort to reduce crosstalk, we used lower intensities of blue light on the y-iLight system. While crosstalk was reduced with decreasing intensity of blue light, at 2.5% blue light mCitrine expression was still observed (Supplementary Fig. 7a). At this point, mTurquoise expression of BY474B had also been sizably reduced (Supplementary Fig. 7b) and thus lowering blue light intensity was not an ideal solution, as the levels of blue light that would preclude cross-activation would also severely hamper expression from the blue light channel.

Supplementary Note 5: Engineering of crosstalk elimination

To address the issues of crosstalk, we tested various fusions of the PSD and light inducible NES to y-iLight, varied the NLS of y-iLight, and experimented with adding PSD to the downstream reporter mCitrine, with a total of 11 constructs were implemented (See Supplementary Table 1 for naming, Supplementary Fig. 8).

All variants were tested by culturing on the Optobox in dark conditions, 25% red light, 25% blue light and 25% blue light supplemented with 50% NIR light. mCitrine fluorescent levels were measured at 8 hours to examine crosstalk activity at and 24 hours and compared to the original BY474R (Supplementary Fig. 8).

To better understand the effects of protein engineering on crosstalk, five metrics were used. 1) Relative strength was calculated as a ratio of mCitrine expression in 25% red light of the specific strain to the mCitrine expression of XTR-1 (the unmodified y-iLight) in 25% red light. 2) Induction fold refers to the ratio mCitrine fluorescence in 25% red light against the same strain in darkness, to indicate the dynamic range of expression. 3) A blue light crosstalk score was calculated through the following formula:

$$\text{Equation 1: } \sqrt{\frac{Expression\ in\ 25\%\ red\ light}{Expression\ in\ 25\%\ blue\ light} * Induction\ fold}$$

The geometric average of the induction fold as well as the ratio of expression in red light to blue light was used to measure the change in baseline due to crosstalk without compromising on activation strength, where high scores would reflect high expression in red light with proportionately low expression in both dark and blue light conditions on a linear scale, and can be easily compared with the original strain. 4) A blue plus NIR light crosstalk was calculated in the same manner, replacing 'Expression in blue light' with the expression level in blue light supplemented with 50% NIR light. 5) An unpaired two tailed t-test was used to determine whether each strain's crosstalk in blue or blue plus NIR light was significantly higher or lower than the strain's baseline expression in darkness.

Based on a comparison of all 12 strains constructed across the two timepoints and multiple illumination conditions, 3 variants of the y-iLight were designated as successfully engineered for lack of crosstalk due to their high crosstalk scores, retention of induction strength and lack of significantly higher expression in blue light than their baseline leakiness in darkness. These strains were also able to maintain these metrics in both the exponential and stationary phases.

y-iLightLINX(PSD) and y-iLightmA1LINX were selected as they demonstrated moderate relative strength and induction folds while reducing crosstalk in blue plus NIR conditions to insignificant levels (Supplementary Fig. 8a-vi, ix, b-vi, ix), with y-iLightLINX(PSD) requiring the fusion of PSD to the downstream gene product while y-iLightmA1LINX did not. y-iLightmA6LEXY was also selected because even though relative strength and induction fold was lower, it demonstrated the highest expression for a strain that could significantly eliminate crosstalk without the presence of NIR light nor fusion of PSD modules (Supplementary Fig. 8a-xi, b-xi).

a

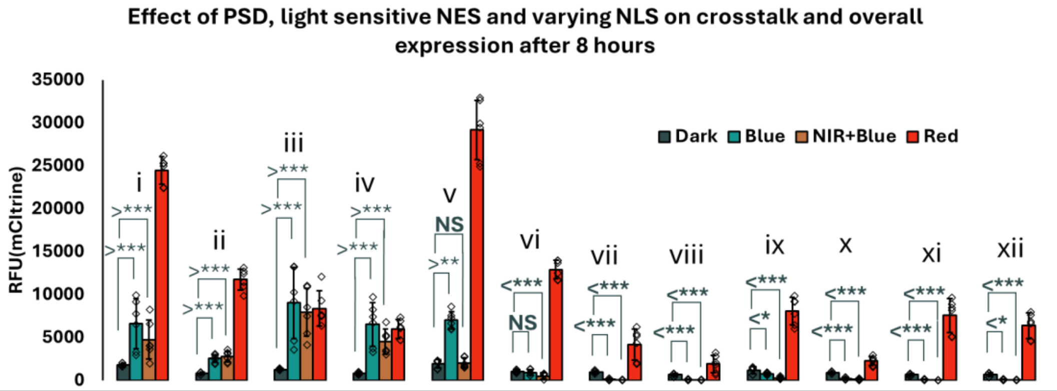

m-Citrine C-Terminal	-	PSD	-	PSD	-	PSD	-	PSD	-	PSD	-	PSD
NLS	SV40	SV40	SV40	SV40	M-A6	M-A6	M-A1	M-A1	M-A1	M-A1	M-A6	M-A6
y-iLight C-Terminal	-	-	PSD	PSD	LINX	LINX	LEXY	LEXY	LINX	LINX	LEXY	LEXY
Relative strength	1	0.48	0.34	0.25	1.19	0.53	0.17	0.08	0.33	0.09	0.31	0.26
Induction fold	13.46	14.06	6.62	7.29	15.05	12.37	4.32	2.95	6.98	2.62	11.33	9.27
Crosstalk Score(Blue)	7.05	8.06	2.48	2.59	7.92	13.11	13.75	10.92	8.47	5.5	49.85	37.76
Crosstalk Score(NIR)	8.32	7.72	2.65	3.11	14.71	17.79	22.2	17.19	13.69	6.93	143.49	69.01
p-value (Blue)	0.00253	1.964E-06	6.13E-05	2.03E-05	1.40E-07	0.0980	3.805E-08	1.92E-06	0.072	1.096E-06	2.95E-07	8.64E-06
p-value (NIR)	0.00111	5.38E-06	8.16E-06	3.62E-05	0.620	0.00262	1.24E-08	1.22E-06	0.000715	1.002E-07	1.052E-07	6.06E-06

b

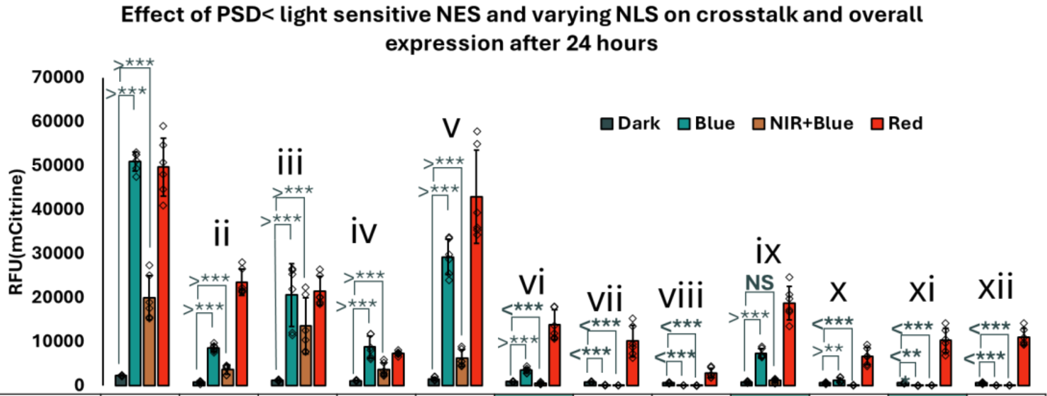

m-Citrine C-Terminal	-	PSD	-	PSD	-	PSD	-	PSD	-	PSD	-	PSD
NLS	SV40	SV40	SV40	SV40	M-A6	M-A6	M-A1	M-A1	M-A1	M-A1	M-A6	M-A6
y-iLight C-Terminal	-	-	PSD	PSD	LINX	LINX	LEXY	LEXY	LINX	LINX	LEXY	LEXY
Relative strength	1	0.47	0.43	0.15	0.86	0.28	0.2	0.06	0.38	0.14	0.21	0.22
Induction fold	21.78	28.09	17.98	6.34	28.74	15.29	11.44	4.51	23.97	10.56	16.45	17.4
Crosstalk Score(Blue)	4.61	8.78	4.34	2.3	6.5	7.81	29.19	20.3	7.81	7.67	100.1	51.57
Crosstalk Score(NIR)	7.35	13.39	5.34	3.57	14.08	20.03	38.94	26.33	19.28	30.33	65.51	67.01
p-value (Blue)	9.81E-14	8.43E-10	5.249E-05	1.993E-05	1.14E-08	1.127E-06	5.26E-08	3.75E-07	4.60E-08	0.0371	4.86E-07	2.29E-05
p-value (NIR)	6.36E-06	7.07E-05	0.000819	0.00254	0.000147	0.00454	8.34E-09	1.88E-07	0.115	1.06E-05	2.51E-07	1.43E-05

Supplementary Fig. 8: Iterative modular engineering of y-iLight with comparison metrics. a Average normalized mCitrine readings for the various iterations of the y-iLight system after culturing in darkness, 25% blue light, 25% blue plus 50% NIR light or 25% red light for 8 hours at 30°C in a shaking incubator. **b** Average normalized mCitrine readings for the various iterations of the y-iLight system after culturing in darkness, 25% blue light, 25% blue plus 50% NIR light or 25% red light for 24 hours at 30°C in a shaking incubator. Values are an average of 6 biological replicates. Error bars indicate standard deviation with a sample

size of n=6, results compared with 2-tailed independent t-test, NS = $p>0.1$, * = $p<0.1$, ** = $p<0.05$, *** = $p<0.01$. Brackets indicate t-tests conducted on expression by strains in either blue light or blue plus NIR light compared to baseline expression in dark conditions. For comparisons where expression in blue light is significantly less than darkness, results of the t-test are bolded. Table below each graph indicates the alterations to y-ILight and mCitrine in the strain, as well as the numerical values of the relative strength, induction fold and crosstalk scores. Columns indicating the metrics of the 3 finalized OY- β strains are highlighted. P-values of the statistical test of each strain in dark conditions compared to blue light or blue+NIR light are listed as rows in each table.

Supplementary Note 6: Effect of varying periods of duty cycles on dual channel strains

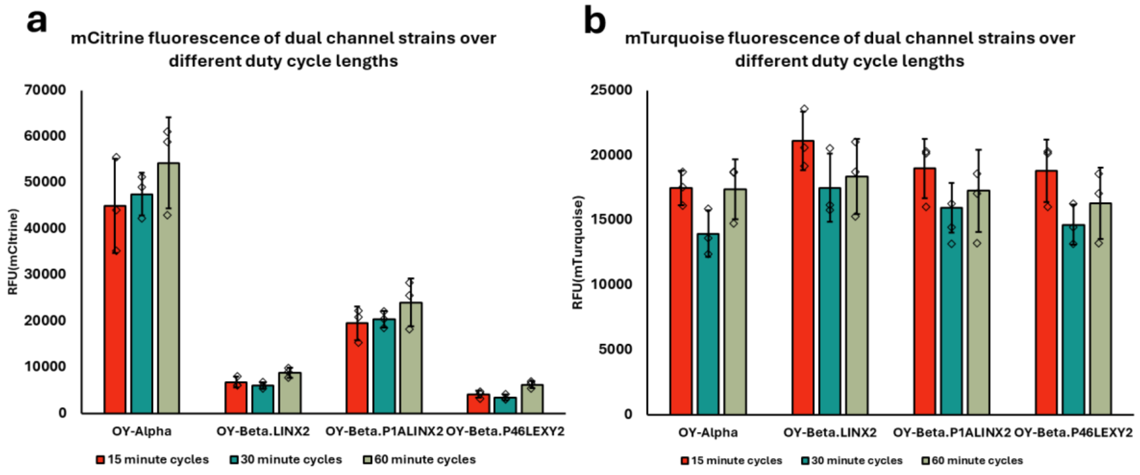

Supplementary Fig. 9: Effect of different duty cycle periods. OY-α, OY-β.LINX, OY-β.mA1LINX and OY- β.mA6LEXY were kept at 30ºC in a shaking incubator in cycles of 15 minutes/30 minutes/60 minutes of 25% blue plus 50% NIR light followed by an equal period of 25% red light. After 24 hours, mCitrine fluorescence (**a**) as well as mTurquoise (**b**) were measured and normalized to OD_{600}. Values are an average of 3 biological replicates. Error bars indicate standard deviation with a sample size of n=3. Values are an average of 3 biological replicates. Error bars indicate standard deviation with a sample size of n=3.

Different lengths of duty cycles were tested to derive a condition where both channels could be activated simultaneously. While blue light channel showed similar levels for all cycle lengths (Supplementary Fig. 9b), 60 minute cycles gave slightly higher levels of expression for all dual channel strains compared to 15 or 30 minute cycles (Supplementary Fig. 9a). Thus, 60 minute cycles were chosen for testing.

Supplementary Note 7: Luteolin production construction and optimization

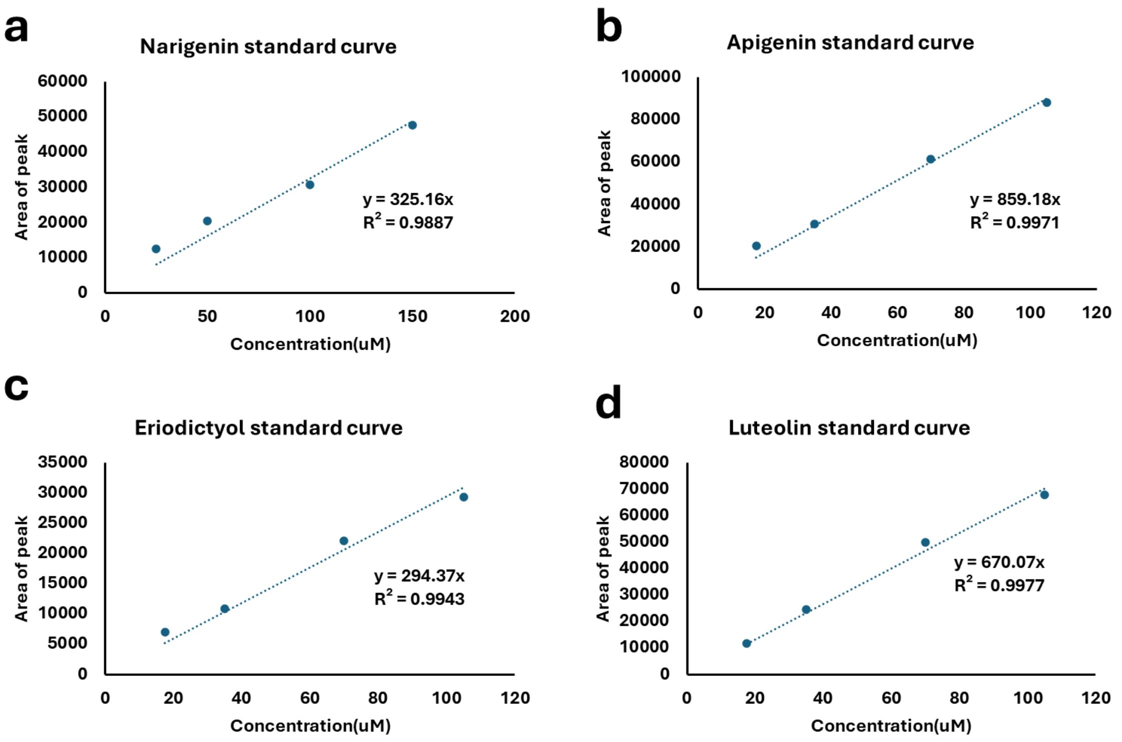

Supplementary Fig. 10: Standard curves for flavonoids. a Standard curve of area under the curve as detected by HPLC for naringenin. Naringenin standard curve was prepared using 25uM, 50uM, 100uM and 150uM of naringenin. **b** Standard curve of area under the curve as detected by HPLC for apigenin. **c** Standard curve of area under the curve as detected by HPLC for eriodictyol. **d** Standard curve of area under the curve as detected by HPLC for luteolin. Apigenin, eriodictyol and luteolin standard curve was prepared using 17.5uM, 35uM, 70uM and 105uM of their respective chemical.

While constructing OY- γ.LutB/R(D), the cytochrome P450 reductase (CPR) gene from the *S. cerevisiae* genome was also cloned under the constitutive promoter P_{PGK1}, as it was established that CPR activity was necessary for F3'H functionality[5,6], and overexpression of native *CPR* from an episomal plasmid has been shown to be sufficient[7].

Initial testing for luteolin production pathway was carried out using FNSI without degron attached, using 300uM of naringenin as substrate. Strains were grown in the dark, in blue light+NIR or 1 hour cycles of blue light plus NIR and red light (Supplementary Fig. 11). OY-γ.LutB/R showed that even in darkness and blue plus NIR light conditions, there was conversion of naringenin to apigenin. The strain did not produce luteolin, but produced both intermediates, indicating that FNSI and F3'H were both functional. Our initial hypothesis was that *FNSI* had a high affinity for naringenin, and thus the basal, uninduced level of enzyme was sufficient to convert a significant amount to apigenin. Its affinity of apigenin may also result in sequestration at induced levels, preventing F3'H from completing the reaction. Hence, to reduce the steady state levels of FNSI, the Ornithrine Decarboxylase Degron (ODC) was added to the C-terminal to destabilize the protein.

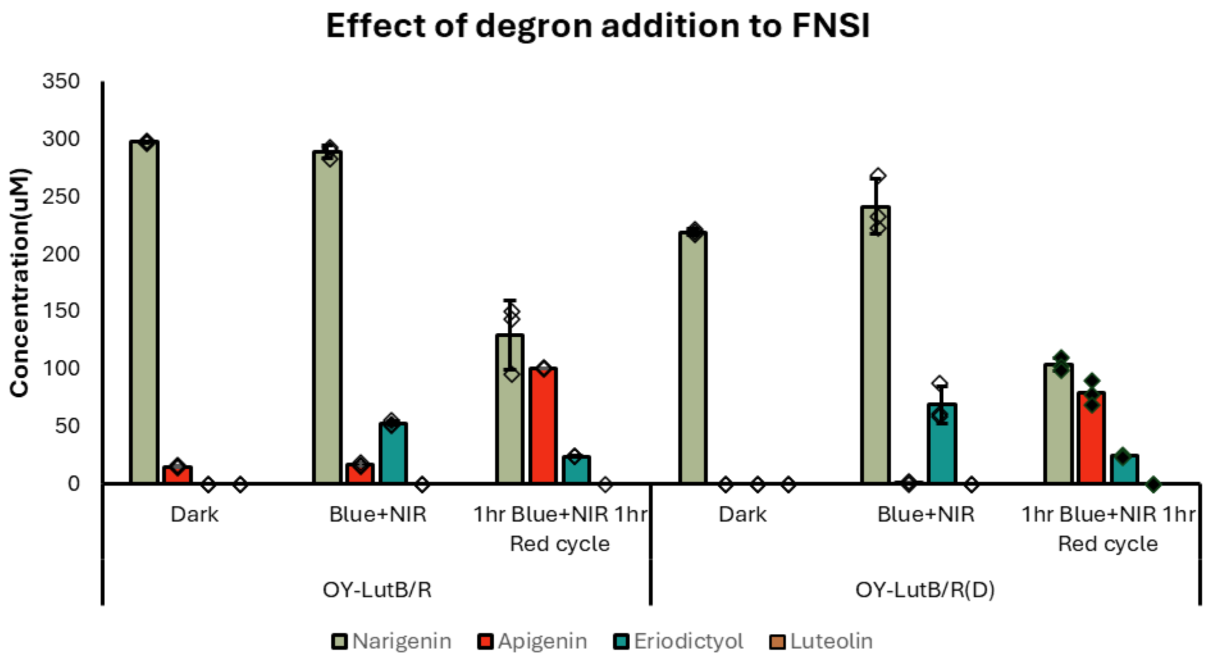

Supplementary Fig. 11: Initial testing of the luteolin production strains under different light conditions, with and without appending a constitutive degron to the C-terminal of the FNSI gene. OY- γ.LutB/R and OY- γ.LutB/R (D) were incubated for 24 hours with 300uM of naringenin substrate at 30°C in a shaking incubator. Strains were incubated wither in darkness, 25% blue plus 50% NIR light, or cycles of 1 hour of 25% blue plus 50% NIR and 1 hour of 25% red light. OY- γ.LutB/R expresses the untagged *FNSI* enzyme in red light, and OY- γ.LutB/R(D) was the strain with ODC degron appended to C-terminal of *FNSI*. Values are an average of 3 biological replicates. Error bars indicate standard deviation with a sample size of n=3. Values are an average of 3 biological replicates. Error bars indicate standard deviation with a sample size of n=3.

After ODC addition, OY- γ.LutB/R(D) showed no detectable conversion of naringenin to apigenin in dark conditions. However, in lit conditions luteolin was still not produced. Thus, we hypothesized that if naringenin was in excess, both enzymes would preferentially bind to naringenin instead of their cognate intermediates, delaying luteolin production until naringenin had been depleted. Thus, for further testing we reduced the concentration of naringenin added to 75 uM.

Supplementary Note 8: Additional testing of luteolin production

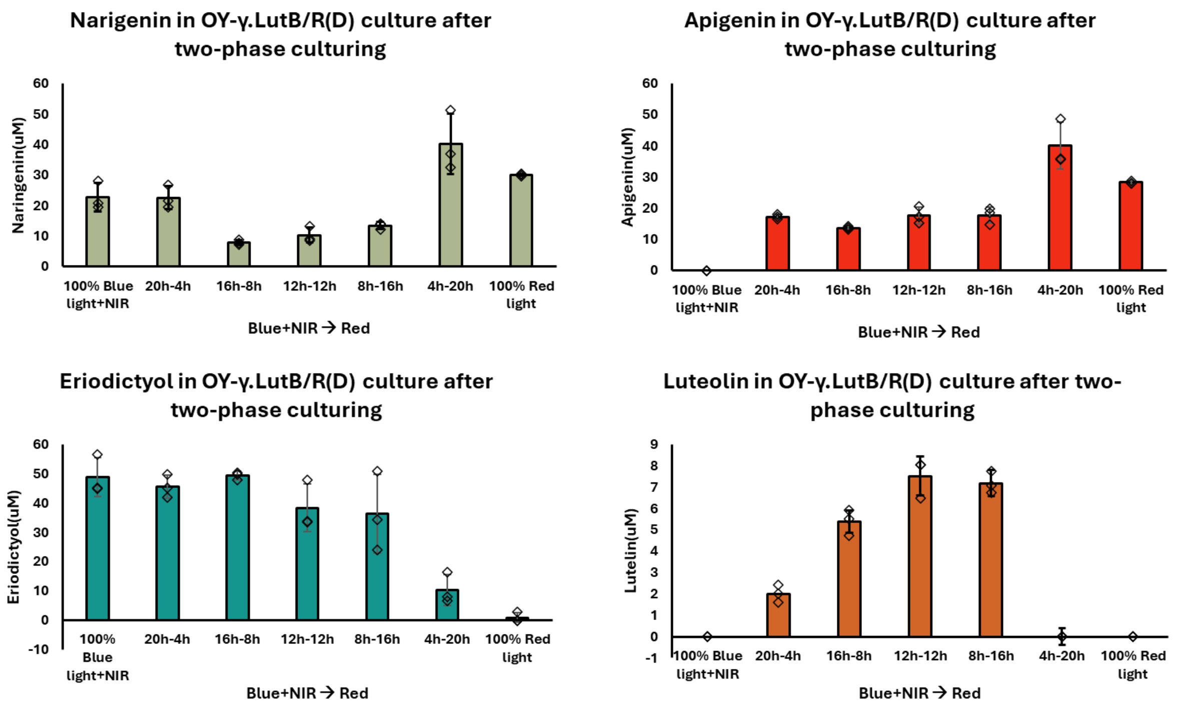

Supplementary Fig. 12: Testing of flavonoid production in 2 phases. OY- γ.LutB/R(D) was cultured at 30°C in a shaking incubator, and illuminated by 25% blue plus 50% NIR light first, followed by red light across a time frame of 24 hours, and the flavonoid profile of the cultures measured by HPLC. Each column is labelled by the ratio of blue plus NIR light to red light in terms of hours. Values are an average of 3 biological replicates. Error bars indicate standard deviation with a sample size of n=3. Values are an average of 3 biological replicates. Error bars indicate standard deviation with a sample size of n=3.

To further investigate the effect of long phases of light cycles, OY- γ.LutB/R(D) was additionally cultured in phases of: (i) 4 hours of blue plus NIR light followed by 20 hours of red light; (ii) 8 hours of blue plus NIR light followed by 16 hours of red light; (iii)16 hours of blue+NIR light followed by 8 hours of red light and (iv) 20 hours of blue plus NIR light followed by 4 hours of red light. We hypothesized that if F3'H activity is limited to an initial phase, redirected cellular resources to expressing FNSI during the period where expressed F3'H is inactive would result in more efficient expression of the pathway on a whole.

As expected, with increasing duration of blue plus NIR light phase, more eriodictyol was produced (10.4 uM-45.7 uM), and with increasing duration of red light phase, more apigenin was produced (17.3 uM-40.1 uM) (Supplementary Fig. 12). However, maximum yield of luteolin did not exceed what was observed in two 12 hour phases, and remained less than what was obtainable by duty cycles.

Supplementary Note 9: RT-qPCR primer optimization

Supplementary Table 2: RT-qPCR primers

Gene	Forward	Reverse	Efficiency
F3’H	TTACGGATCAGTTCGGCAATAG	CGCGGATAATGATGGAGGTAAG	88%
FNSI	CGCGATGCTCAAGCGGCTAGA	TATTACAAGACATGGTTGGGGGTTTGCAAG	116%

Supplementary Note 10: Model equations for modelling luteolin production pathway

Supplementary Table 3: Model formulations for enzymes and flavonoids

Model ODEs	No.
$\frac{d[mFNSI]}{dt} = K_{synMFNSI} * R - degM * [mFNSI]$	Eq. 1
$\frac{d[mF3H]}{dt} = K_{synMF3H} * B - degM * [mF3H]$	Eq. 2
$\frac{d[FNSI]}{dt} = K_{prot} * [mFNSI] - degP * [FNSI]$	Eq. 3
$\frac{d[F3H]}{dt} = K_{prot} * [mF3H] - degP * [F3H]$	Eq. 4
$\frac{d[Nar]}{dt} = -k_{catNA} * [FNSI] * \left(\frac{[Nar]}{[Nar] + K_{mNA}}\right) - k_{catNE} * [F3H] * \left(\frac{[Nar]}{[Nar] + K_{mNE}}\right)$	Eq. 5
$\frac{d[Api]}{dt} = k_{catNA} * [FNSI] * \left(\frac{[Nar]}{[Nar] + K_{mNA}}\right) - k_{catAL} * [F3H] * \left(\frac{[Api]}{[Api] + K_{mAL}}\right)$	Eq. 6
$\frac{d[Eri]}{dt} = k_{catNE} * [F3H] * \left(\frac{[Nar]}{[Nar] + K_{mNE}}\right) - k_{catEL} * [FNSI] * \left(\frac{[Eri]}{[Eri] + K_{mEL}}\right)$	Eq. 7
$\frac{d[Lut]}{dt} = k_{catAL} * [F3H] * \left(\frac{[Api]}{[Api] + K_{mAL}}\right) + k_{catEL} * [FNSI] * \left(\frac{[Eri]}{[Eri] + K_{mEL}}\right)$	Eq. 8
$[mFNSI]$ and $[mF3H]$ represent the mRNA expression levels for FNSI and F3'H enzymes; $[FNSI]$ and $[F3H]$ are the protein levels of the two enzymes; $[Nar]$, $[Api]$, $[Eri]$, and $[Lut]$ denote the concentration levels of naringenin, apigenin, eriodictyol, and luteolin.	

Supplementary Table 4: Details of estimated model kinetic parameters

Parameter	Description	Value	Unit
$K_{synMFNSI}$	Transcriptional rate for FNSI	7.79e-2	min^{-1}

$K_{synMF3H}$	Transcriptional rate for F3'H	1.87e-1	min^{-1}
$degM$	mRNA degradation rate	3.5e-2 (fixed)	min^{-1}
K_{prot}	Translational rate	1.22e-2	min^{-1}
$degP$	Protein degradation rate	1.36e-3	min^{-1}
k_{catNA}	Catalytic rate constant for FNSI to convert naringenin to apigenin	3.88e-3	min^{-1}
k_{catNE}	Catalytic rate constant for F3'H to convert naringenin to eriodictyol	2.78e-3	min^{-1}
k_{catAL}	Catalytic rate constant for F3'H to convert apigenin to luteolin	1.69e-5	min^{-1}
k_{catEL}	Catalytic rate constant for FNSI to convert eriodictyol to luteolin	1.23e-3	min^{-1}
K_{mNA}	Half-activation constant	2.07e1	µM
K_{mNE}	Half-activation constant	3.34e1	µM
K_{mAL}	Half-activation constant	2.39e1	µM
K_{mEL}	Half-activation constant	3.77e1	µM

Supplementary Note 11: Flocculation construction and testing

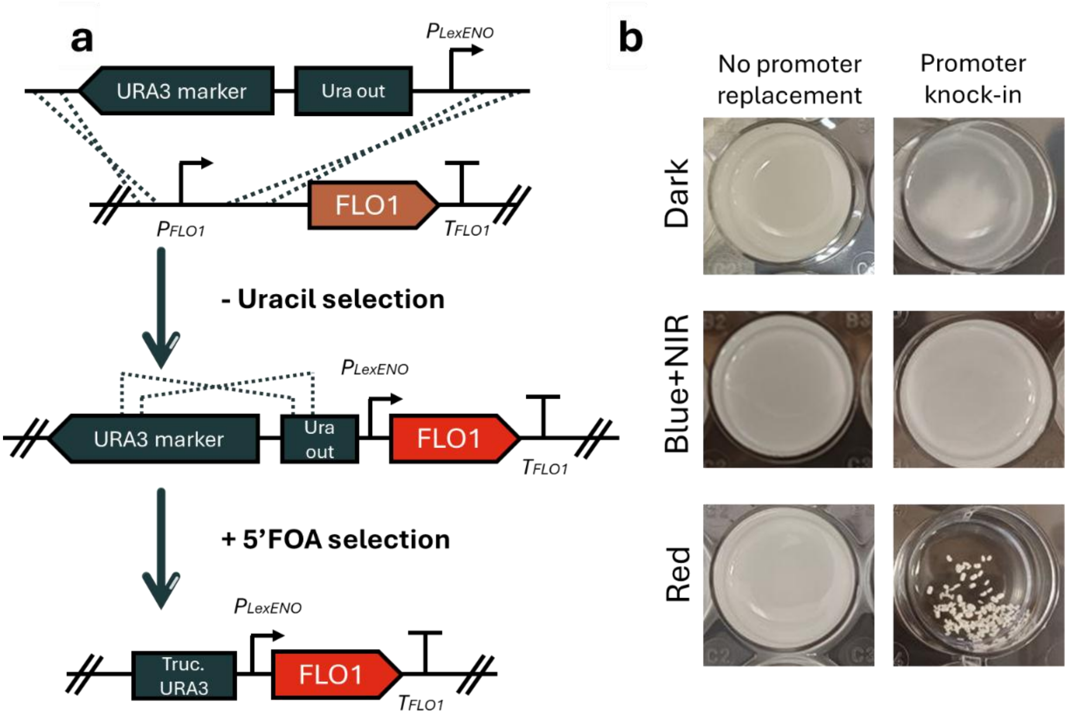

Supplementary Fig. 13: Construction of strain with red-light inducible flocculation. a Genetic diagram of knock in and counterselection of $P_{lexENO1}$ promoter. **b** Images of cells with or without promoter replacement after exposure to **24** hours of darkness, 25% blue plus 50% NIR light or 25 % red light at 30°C incubation with shaking.

To introduce optogenetic controlled flocculation, we chose to investigate the gene *FLO1*, whose overexpression can instigate flocculation in *S. cerevisiae*[8]. Lab strains are unable to flocculate naturally due to a nonsense mutation in regulatory protein FLO8[9]. However, by replacing the wildtype promoter of *FLO1* with a heterologous promoter, FLO1 expression can be re-established[10]. While optogenetic control of FLO1 has been previously demonstrated[10], it has yet to be paired with red light control, or used in conjunction with the biomanufacturing of any product.

Overlap PCR was used to construct a fragment containing $P_{lexENO1}$ promoter, a URA3 marker and a region homologous to the upstream region of URA3, with ends homologous to the promoter region of *FLO1*. Fragment was knocked into the genome using uracil auxotrophy selection, and 5'FOA counterselection was used to select for a colony where homologous recombination had deleted the URA3 gene so that the marker could be recycled (Supplementary Fig. 13a). To test the flocculation ability of this strain, yeasts with and without the promoter knock-in were cultured on Optoboxes in darkness, blue plus NIR light as well as red light for 24 hours (Supplementary Fig. 13b). Strains without knock-in demonstrated no observable flocculation, but strains with knock-in flocculated heavily in red light. No flocculation was observed in blue plus NIR light, although some minimal flocculation was observed for cultures kept in the dark, indicating that there may be some leaky expression of the *FLO1* gene. It was concluded that promoter replacement successfully conferred the ability to flocculate in red light, and was not inadvertently activated by a combination of blue plus NIR light.

Supplementary Note 12: Flocculation testing flavonoid profiles

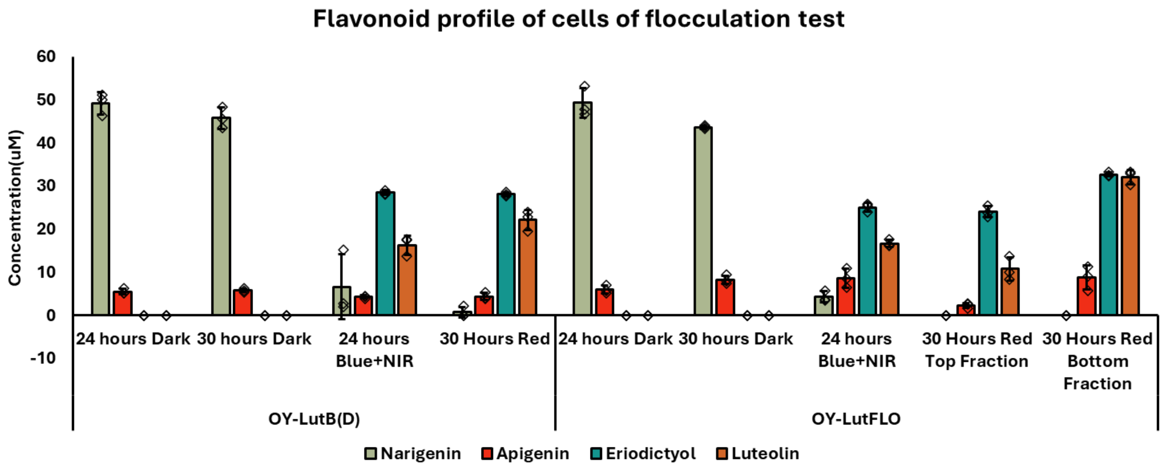

Supplementary Fig. 14: Flavonoid profiles of OY- γ.LutB(D) and OY- γ.LutFlo during flocculation testing. Both strains were kept in either darkness for 30 hours, or illuminated with 25% blue plus 50% NIR light for 24 hours followed by 6 hours of 25% red light in a 30°C shaking incubator. After flocculation, both the supernatant(Top fraction) and the remaining 1 mL of media with flocs present (Bottom fraction) were tested for their flavonoid profile. Values are an average of 3 biological replicates. Error bars indicate standard deviation with a sample size of n=3. Values are an average of 3 biological replicates. Error bars indicate standard deviation with a sample size of n=3.

Supplementary Note 13: Construction of dual coloured image printing

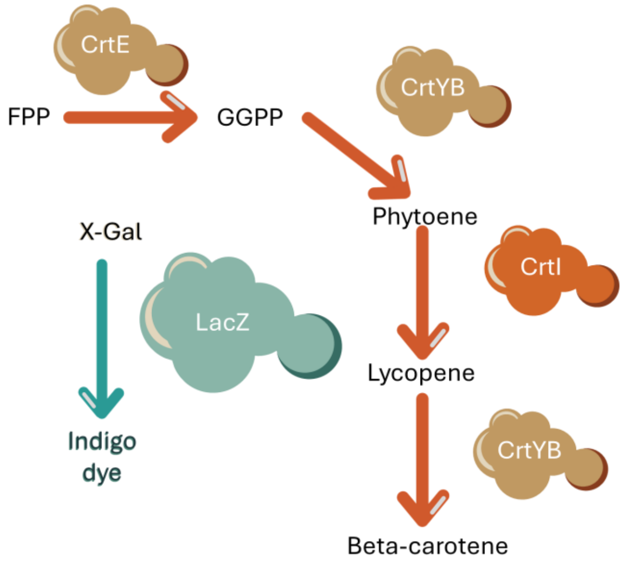

Supplementary Fig. 15: Diagram of the two biosynthesis pathways used to produce different coloured dyes in OY- γ.RB.

To achieve multi-coloured images, two coloured chemicals were produced under the two optogenetic channels. The *lacZ* gene was cloned downstream of P_{6CCL} such that in the presence of blue light, X-Gal would be catalyzed to a blue dye. *CrtE* and *CrtYB* were constitutively expressed from plasmid pLM494[4], with the promoter upstream of *CrtI* replaced with $P_{lexENO1}$ such that colourless phytoene would be constitutively produced from *S. cerevisiae* metabolites[11], and in the presence of red light *CrtI* would be expressed, completing the metabolic circuit to convert phtyoene to bright orange β-carotene. The cassette expressing *tHMG1* in pLM494 was also deleted, as it had been shown that this increases the intensity of orange colour observed in colonies[12]. OY- β.mA1LINX was cloned was reconstructed with these pathways and designated OY- γ.RB (Red-Blue).

Supplementary Note 14: Setup of Optobox for dual colour image printing

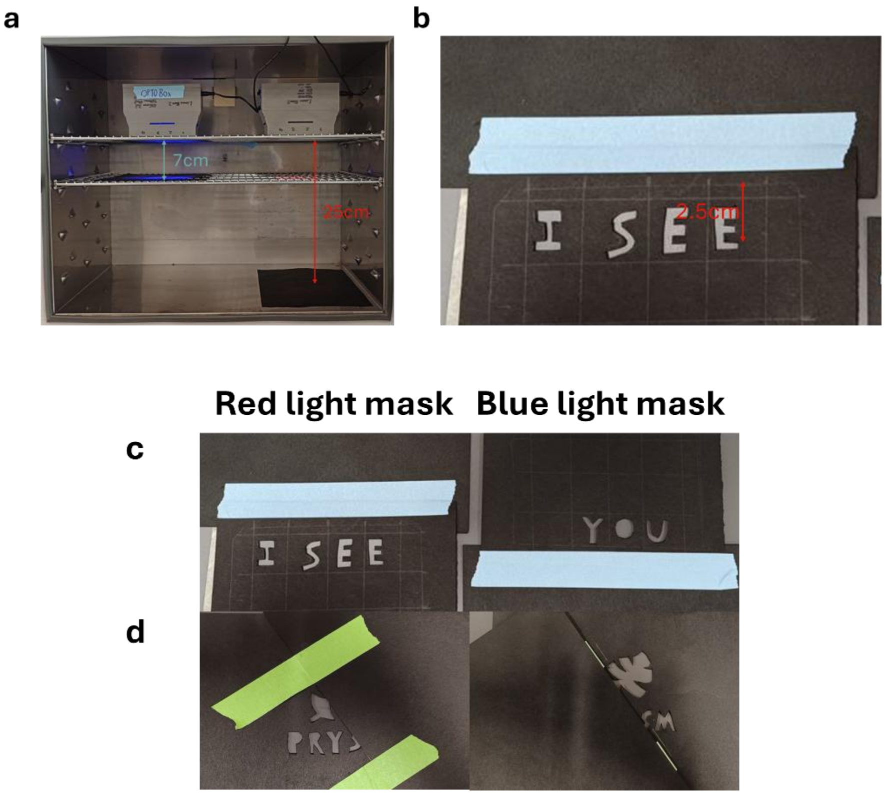

Supplementary Fig. 16: Setup for optogenetic image printing. a Setup for optoboxes relative to the biofilm of yeast used. **b** Rough size of the masks used during the printing process. **c, d** Masks used to imprint Fig. 7b and c respectively.

For optogenetic pattern printing on *S.cerevisiae*, Optoboxes were inverted and placed on grills inside a 30 ºC incubator (Supplementary Fig. 16a). To localize light exposure to desired areas, a pen knife was used to carve patterns in sheet of black paper. Patterns on the scale of about 2.5 cm in radius and 0.5 cm in aperture size are appropriate for this method (Supplementary Fig. 16b). After layer of cells is produced in accordance with the protocol stated in methods, the agar plate was first exposed to red light. Red light optobox was set to 5% intensity, and plate was placed agar side up at a distance of 25cm away from light source. Pattern was overlayed on the plate and left for 24 hours. The plate was then exposed to blue light, at 100% intensity and a distance of 7 cm for another 24 hours.

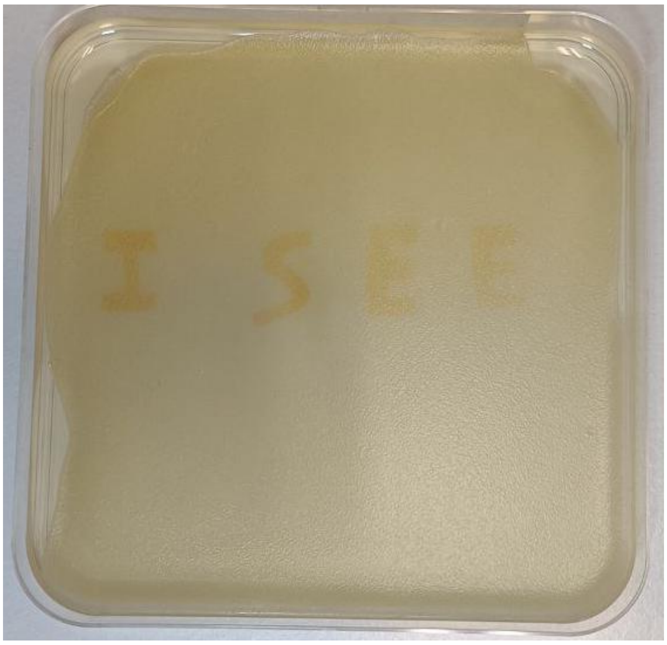

Supplementary Fig. 17: Negative control of optogenetic image printing to check for blue light crosstalk. Biofilm exposed to both blue and red light without the use of X-gal in the agar.

As a negative control, OY- γ.RB was layered on agar without X-gal added, and exposed to the same masking and conditions as seen in Fig. 7b (Supplementary Fig. 17). The blue light patterning was not observed in beta-carotene, indicating that for this set-up, blue light without NIR light did not induce any crosstalk.

Supplementary Note 15: Schematics for Optobox

To support optogenetic experiments in the lab, a reliable light illumination device is needed. As most of our optogenetic experiments in our lab are conducted using 12-well plates, we sought to develop a low cost and robust light illumination device (hereafter named Optobox) tailored for 12-well plates. Designed for ease of assembly and operation, it is meant to be accessible to researchers with minimal electrical expertise.

We included the 3 primary colors for each well – Red (655 nm), Green (520 nm) and Blue (465nm), along with a near-infrared (NIR) wavelength (770 nm). We opted for 5 mm LEDs for ease of procurement and assembly compared to surface-mounted (SMD) LEDs. The selected LEDs are detailed in Supplementary Table 5. It should also be noted that the LEDs can be easily changed for other wavelengths as required.

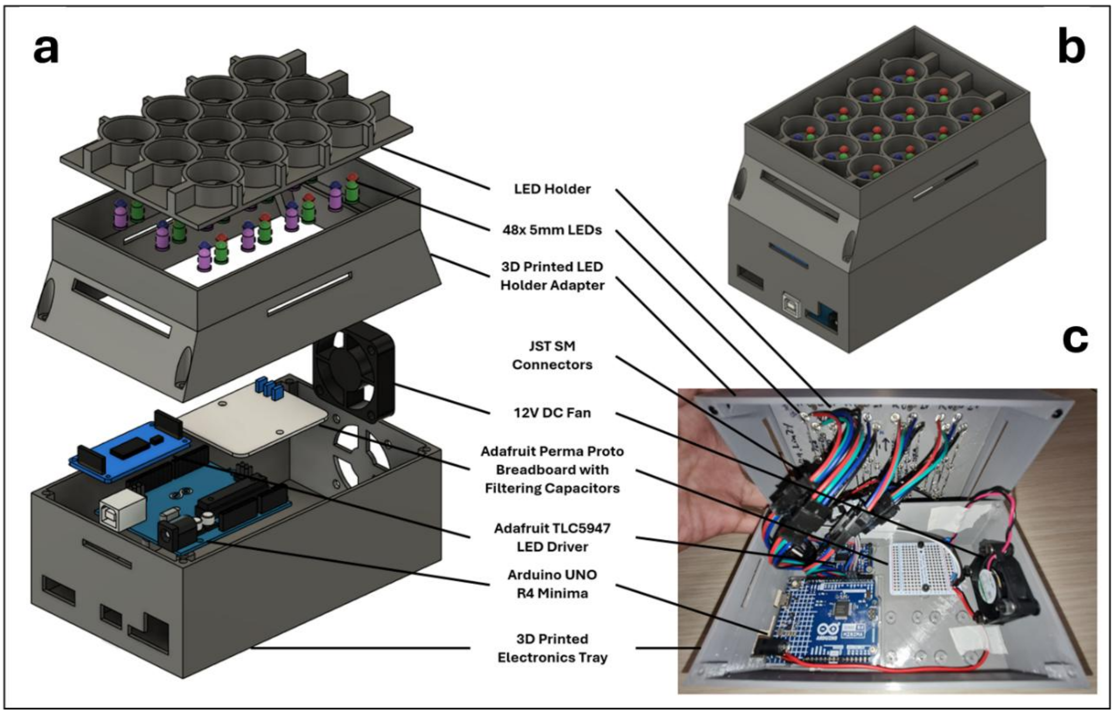

Supplementary Fig. 18. Illustration of the Optobox device. **a** Exploded view of the Optobox showing the components and their layout. The 3D model of the Arduino UNO was obtained from Autodesk TinkerCAD. **b** 3D render of the completed Optobox. **c** Actual photo showing the electronics within the Optobox.

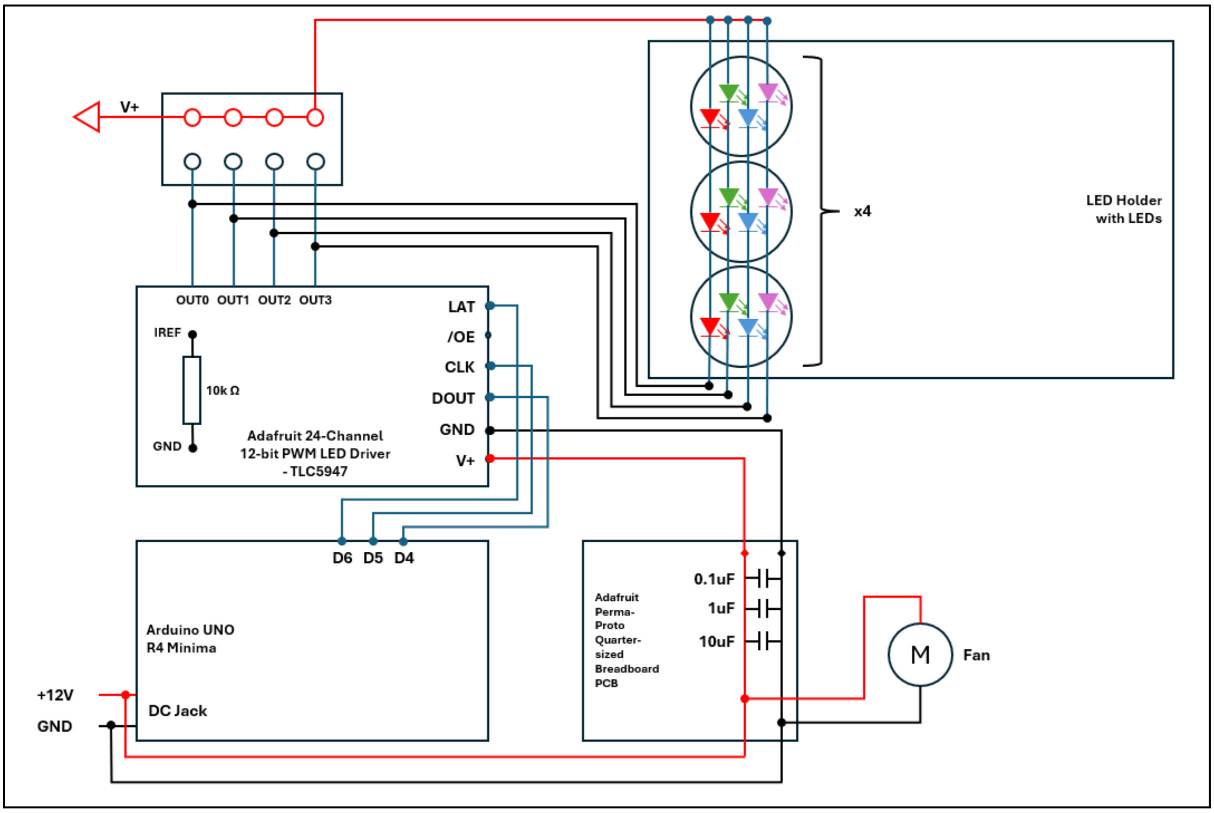

Supplementary Fig. 19. Circuit diagram of the electronic components of the Optobox.

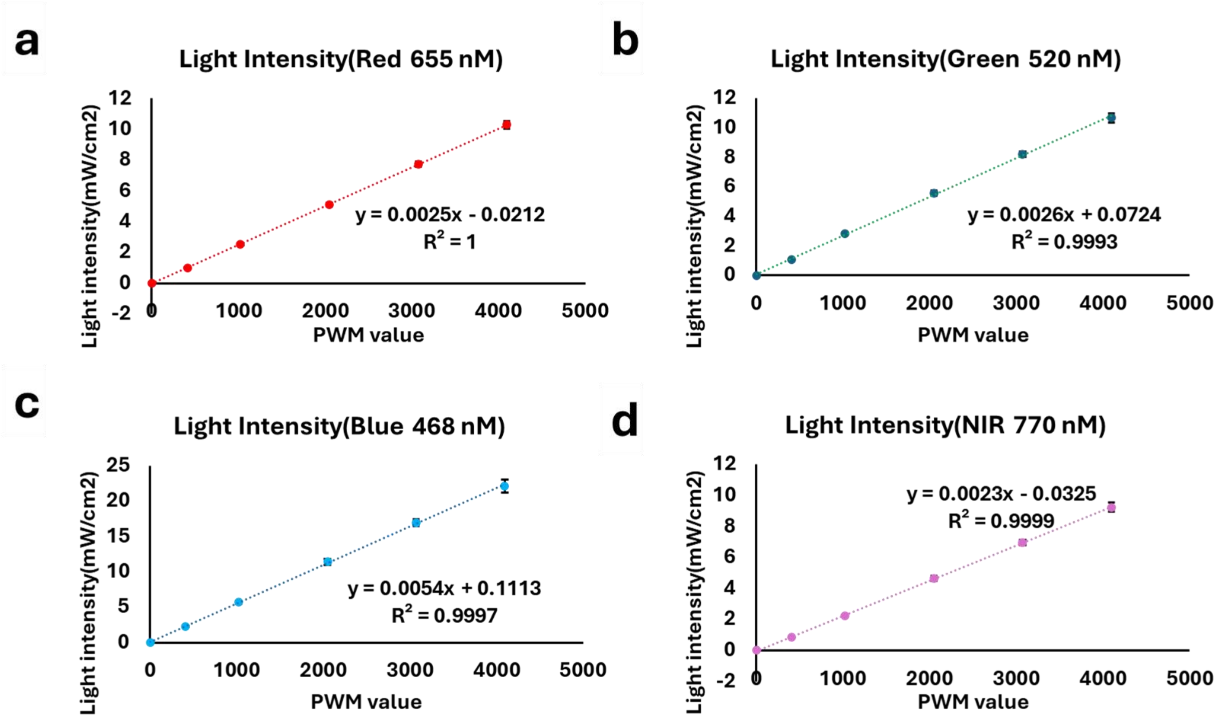

Supplementary Fig. 20. Light intensities at each PWM setting on the Optobox, from 0 to 4,095 for a total of 4,096 intensity levels. a For the Red 655nM LEDs. **b** For the Green 520nM LEDs. **c** For the Blue 468nM LEDs. D) For the Near Infrared-Red 770nM LEDs.

Supplementary Table 5. List of LEDs Used.

Color	Wavelength (nM)	Output	Model	Manufacturer
Blue (468nM)	468	4cd	MP008538	MULTICOMP PRO
Green (520nM)	520	13cd	OVL-5524	Multicomp Pro
Red (655nM)	655	7cd	MCL053RHC	Multicomp Pro
NIR (770nM)	770	5.5mW	MTE1077N1-R	Marktech Optoelectronics

Supplementary Note 16: Characterization of the effect of high intensity blue light on native *S. cerevisiae* promoters

Through the course of testing the EL222 blue light system and it's cognate P_{6CCL} promoter, a reference promoter was used to evaluate whether the performance was of comparable magnitude to other native, constitutive promoters that were well characterized and commonly used in other industrial and research applications. It was observed that the high intensities of blue light had a negative effect on the native promoters of *S. cerevisiae,* although no growth reduction was observed at the high blue light intensities studied (Supplementary Fig. 1).

Initially, P_{PGK1} and P_{ACT1} constitutive promoters from the *S. cerevisiae* genome were used to express fluorescent protein mTurquoise for comparison, but a sharp drop in fluorescence from P_{PGK1} was observed in 50% blue light, and a sharp drop in fluorescence from both P_{PGK1} and P_{ACT1} were observed in 100% blue light (Supplementary Fig. 21). P_{6CCL} did not experience this drop in fluorescence. Cycles of 30 minutes of 100% blue light and 30 minutes of darkness restored fluorescence expression by P_{PGK1} and P_{ACT1} to levels similar to those observed in dark conditions, while P_{6CCL} remained at similar levels (Supplementary Fig. 21).

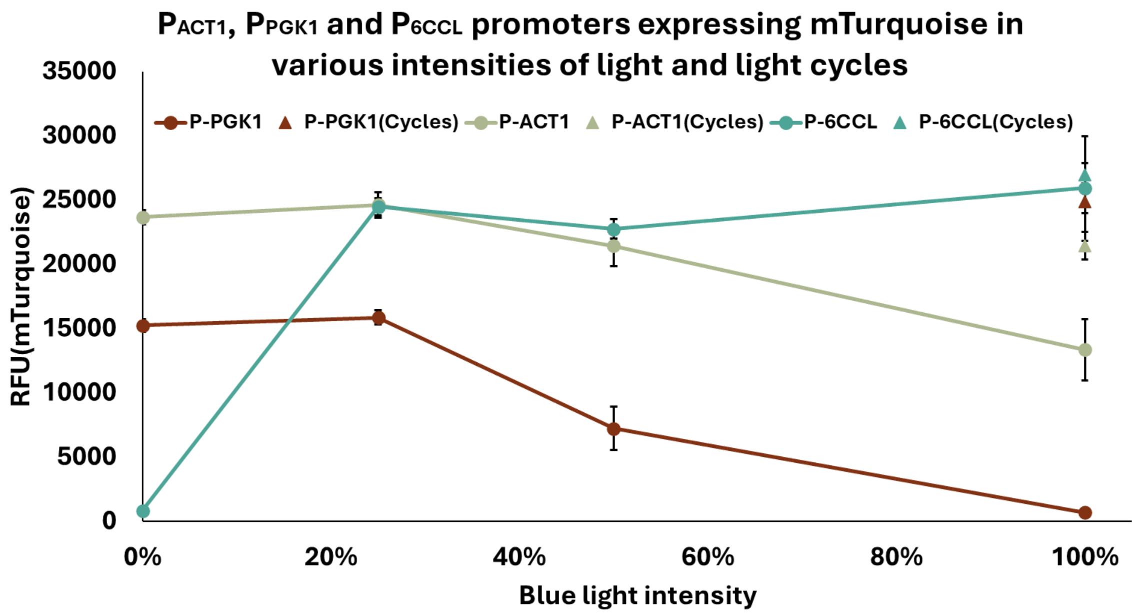

Supplementary Fig. 21: Expression of mTurquoise fluorescent reporter under P_{6CCL}, P_{ACT1} and P_{PGK1} control in 0-100% blue light. Cells were cultured at 30ºC in a shaking incubator while exposed to 0%-100% blue light (See Supplementary Fig. 19 for operational values of light intensity), as well as cycles of 30 minutes of 100% blue light followed by 30 minutes of darkness (Represented by triangle data points). Fluorescence was measured at 24 hours and normalized to OD_{600}. Values are an average of 3 biological replicates. Error bars indicate standard deviation with a sample size of n=3.

Examination of the OD_{600} values of BY474B as well as the strains harbouring plasmids expressing mTurquoise under P_{PGK1} and P_{ACT1} across the exponential phase revealed that high intensity of blue light did not significantly reduce the growth of cells, nor did the switch to 30-minute cycles increase the growth in a way that would recover the impaired expression (Supplementary Fig. 1). It has been shown that blue light may reduce oxygen uptake and respiration in yeasts without affecting viability[13], and may occur through damaging the

mitochondrial complex[14]. The P_{PGK1} promoter appears to be more heavily affected by blue light. As the PGK1 enzyme is heavily involved in glycolysis, the P_{PGK1} promoter may be downregulated due to a general impact of blue light on respiration. Alternatively, blue light has been shown to activate a stress response through the Msn2 protein[15], localizing the protein to the cytoplasm. As Msn2p is a key activator of glycolytic enzymes including PGK1[16], this may result in a downregulation specifically of the P_{PGK1} promoter without affecting P_{6CCL}. This contradicts the notion that light is orthogonal and non-toxic to light-insensitive microbes[17–20], and corroborates the need to caution against moderate phototoxic effects[21,22] that may interfere with other aspects of the cells.

To further probe this phenomenon and identify a possible alternative reference promoter that would be unaffected by high blue light intensities, an additional five promoters were chosen, with the hypothesis that since growth was unaffected, these key promoters responsible for various aspects of the *S. cerevisiae* metabolism should remain stable (Supplementary Table 6). The expression of these promoters were tested in dark, 50% and 100% blue light conditions over 24 hours (Supplementary Fig. 22). Unexpectedly, the expression of all promoters had decreased with increase intensity of blue light. This suggests that while this phenomenon was not severe enough to critically affect growth, it was generic enough to impact multiple aspects of *S. cerevisiae* metabolism.

Supplementary Table 6: Table of promoters tested for blue light sensitivity and their respective function in cell metabolism.

Promoter	Purpose
PGK1	Glycolysis and gluconeogenesis[23]
ACT1	Involved in cell polarization and cytoskeleton[24]
TDH3	Glycolysis and gluconeogenesis[25]
TEF1	Translation and tRNA binding[26]
GAP1	Amino acid uptake[27]
GAL1	Galactose metabolism[28]
ADH1	Ethanol metabolism[29]

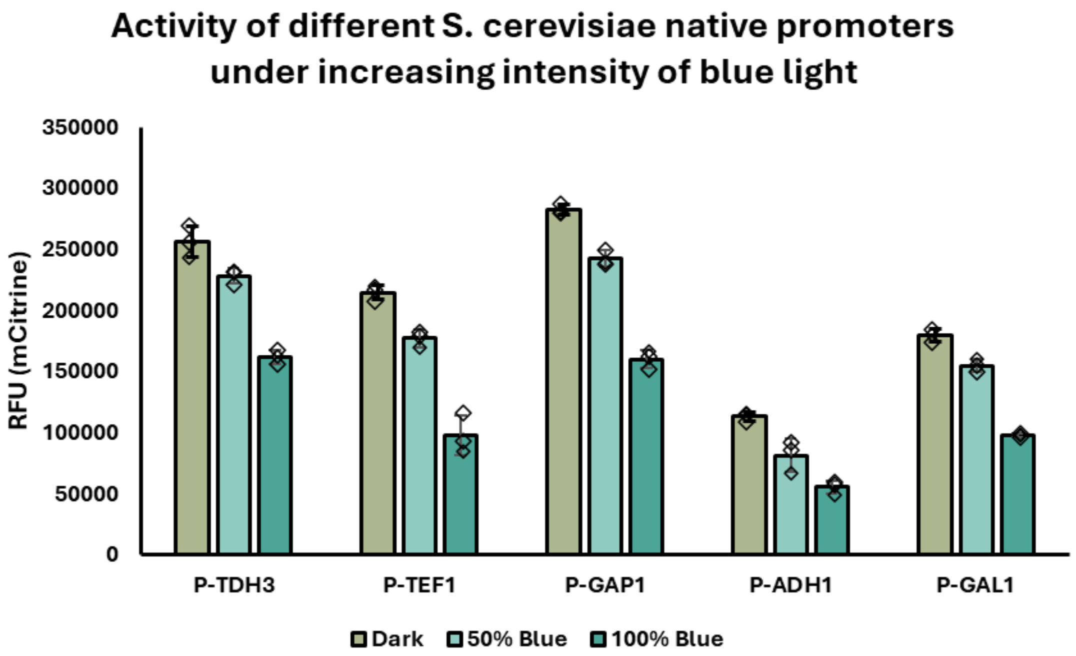

Supplementary Fig. 22: Activity of various constitutive native *S. cerevisiae* promoters in increasing intensities of blue light. Cells expressing mCitrine fluorescent protein from the listed constitutive promoters were cultured at 30°C in a shaking incubator while exposed to darkness, 50% or 100% blue light. Cells harbouring the GAL1 promoter were cultured in 2% galactose, 0.2% glucose YNB media, all other cells were cultured in 2% glucose YNB media. Fluorescence was measured at 24 hours and normalized to OD_{600}. Values are an average of 3 biological replicates. Error bars indicate standard deviation with a sample size of n=3.

To further characterize this phenomenon and attempt to elucidate its mechanism, RT-qPCR was carried out to measure the levels of mRNA transcript of the fluorescent protein produced by both P_{ACT1} and P_{6CCL} under dark, 25% and 100% blue light conditions. Cycling times for transcript amplification was compared against housekeeping gene UBC6, a gene commonly used for RT-qPCR analysis yeast[30].

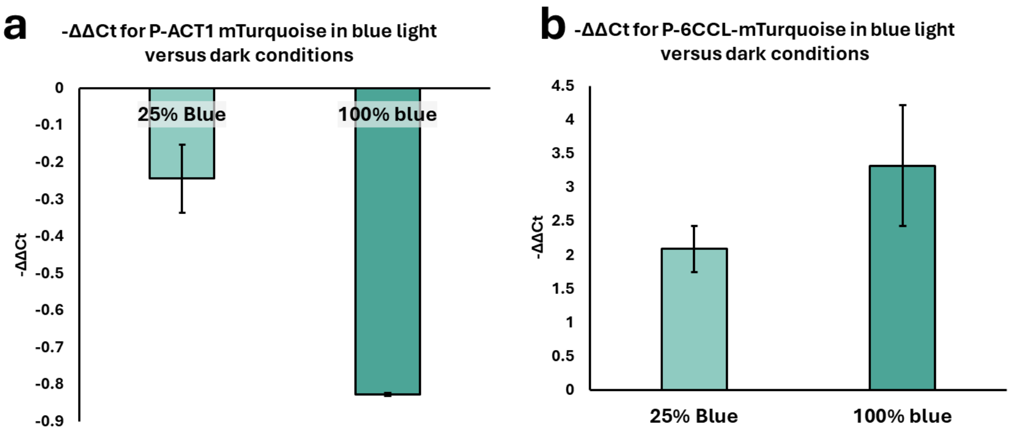

Supplementary Fig. 23: RT-qPCR results for BY474B and BY4741 expressing mTurquoise from P_{ACT1} in blue light compared to darkness. a -ΔΔCt for cells expressing

mTurquoise under the control of P_{ACT1} promoter. Cycling times for mTurquoise RNA was compared against native housekeeping gene UBC6, and conditions in 25% or 100% blue light were compared to dark conditions. Values are an average of 2 biological replicates. Error bars indicate standard deviation with a sample size of n=2. **b** -ΔΔCt for cells expressing mTurquoise under the control of P_{6CCL} promoter. Cycling times for mTurquoise RNA was compared against native housekeeping gene UBC6, and conditions in 25% or 100% blue light were compared to dark conditions. Values are an average of 2 biological replicates. Error bars indicate standard deviation with a sample size of n=2.

RT-qPCR data showed that in 25% and 100% blue light, P_{6CCL} produced 4-fold and 10-fold more mRNA respectively, demonstrating that P_{6CCL} increases transcription even in high intensities of blue light (Supplementary Fig. 23b). Conversely, P_{ACT1} had decreased by 15% and 45% respectively, suggesting that the decrease in fluorescence was likely tied to a decrease in transcription (Supplementary Fig. 23a).

It should be noted that a key assumption made in this experiment was that UBC6 remains relatively stable during blue light illumination. As the cycling times for the amplification of mRNA under P_{ACT1} increased, barring the unlikely case where blue light would increase the expression of UBC6, it can be concluded that mRNA produced by P_{ACT1} decreases in high intensities of blue light, and that the decrease in fluorescent protein would likely be attributed to factors upstream of the transcription.

To the best of our knowledge, this phenomenon has yet to be reported, thus existing literature was then reviewed for the commonly administered intensities of light for blue light optogenetics in *S. cerevisiae*. It was noted that many other publications had used light intensities that were either much lower[31–33], or for much shorter durations[31,34–38] than what was present in our experiments.

Ultimately, as our original purpose was to ensure that the performance of our promoters was comparable to those used in conventional applications and P_{6CCL} was able to maintain its expression even under 100% blue light, further probing into this phenomenon was out of the scope of this paper. Nevertheless, the P_{6CCL} promoter still has expression comparable to the native promoter under its optimal, commonly used dark condition. To ensure reported results were reliable, data in the main paper was compared with P_{ACT1} within 0-50% blue light as it was the promoter least affected by this phenomenon.

Supplementary References

1. Wong, G. *et al.* Reconstituting the complete biosynthesis of D-lysergic acid in yeast. *Nat Commun* **13**, 712 (2022).
2. Kaberniuk, A. A., Baloban, M., Monakhov, M. V., Shcherbakova, D. M. & Verkhusha, V. V. Single-component near-infrared optogenetic systems for gene transcription regulation. *Nature Communications* **12**, 3859 (2021).
3. Ottoz, D. S. M., Rudolf, F. & Stelling, J. Inducible, tightly regulated and growth condition-independent transcription factor in Saccharomyces cerevisiae. *Nucleic Acids Res* **42**, e130 (2014).
4. Hochrein, L., Mitchell, L. A., Schulz, K., Messerschmidt, K. & Mueller-Roeber, B. L-SCRaMbLE as a tool for light-controlled Cre-mediated recombination in yeast. *Nature Communications* **9**, 1931 (2018).
5. Li, G., Li, H., Lyu, Y., Zeng, W. & Zhou, J. Enhanced Biosynthesis of Dihydromyricetin in Saccharomyces cerevisiae by Coexpression of Multiple Hydroxylases. *J. Agric. Food Chem.* **68**, 14221–14229 (2020).
6. Gao, S. *et al.* Efficient Biosynthesis of (2S)-Eriodictyol from (2S)-Naringenin in Saccharomyces cerevisiae through a Combination of Promoter Adjustment and Directed Evolution. *ACS Synth. Biol.* **9**, 3288–3297 (2020).
7. Yu, S., Li, M., Gao, S. & Zhou, J. Engineering Saccharomyces cerevisiae for the production of dihydroquercetin from naringenin. *Microb Cell Fact* **21**, 213 (2022).
8. Stratford, M. Genetic aspects of yeast flocculation: in particular, the role of *FLO* genes in the flocculation of *Saccharomyces cerevisiae*. *Colloids and Surfaces B: Biointerfaces* **2**, 151–158 (1994).
9. Van Mulders, S. E. *et al.* Phenotypic diversity of Flo protein family-mediated adhesion in Saccharomyces cerevisiae. *FEMS Yeast Research* **9**, 178–190 (2009).

10. Salinas, F. *et al.* Fungal Light-Oxygen-Voltage Domains for Optogenetic Control of Gene Expression and Flocculation in Yeast. *mBio* **9**, e00626-18 (2018).
11. Verwaal, R. *et al.* High-Level Production of Beta-Carotene in Saccharomyces cerevisiae by Successive Transformation with Carotenogenic Genes from Xanthophyllomyces dendrorhous. *Applied and Environmental Microbiology* **73**, 4342 (2007).
12. Mitchell, L. A. *et al.* Versatile genetic assembly system (VEGAS) to assemble pathways for expression in S. cerevisiae. *Nucleic Acids Res* **43**, 6620–6630 (2015).
13. Ninnemann, H., Butler, W. L. & Epel, B. L. Inhibition of respiration in yeast by light. *Biochimica et Biophysica Acta (BBA) - Bioenergetics* **205**, 499–506 (1970).
14. Molin, M. *et al.* Protein kinase A controls yeast growth in visible light. *BMC Biology* **18**, 168 (2020).
15. Camponeschi, I., Montanari, A., Mazzoni, C. & Bianchi, M. M. Light Stress in Yeasts: Signaling and Responses in Creatures of the Night. *Int J Mol Sci* **24**, 6929 (2023).
16. Kuang, Z., Pinglay, S., Ji, H. & Boeke, J. D. Msn2/4 regulate expression of glycolytic enzymes and control transition from quiescence to growth. *eLife* **6**, e29938.
17. Benisch, M., Aoki, S. K. & Khammash, M. Unlocking the potential of optogenetics in microbial applications. *Current Opinion in Microbiology* **77**, 102404 (2024).
18. Shimizu-Sato, S., Huq, E., Tepperman, J. M. & Quail, P. H. A light-switchable gene promoter system. *Nature Biotechnology* **20**, 1041–1044 (2002).
19. Hoffman, S. M., Tang, A. Y. & Avalos, J. L. Optogenetics Illuminates Applications in Microbial Engineering. *Annual Review of Chemical and Biomolecular Engineering* **13**, 373–403 (2022).
20. Chia, N., Lee, S. Y. & Tong, Y. Optogenetic tools for microbial synthetic biology. *Biotechnology Advances* **59**, 107953 (2022).

21. Figueroa, D., Rojas, V., Romero, A., Larrondo, L. F. & Salinas, F. The rise and shine of yeast optogenetics. *Yeast* **38**, 131–146 (2021).

22. Lindner, F. & Diepold, A. Optogenetics in bacteria – applications and opportunities. *FEMS Microbiol Rev* **46**, fuab055 (2021).

23. Mazzoni, C., Torella, M., Petrera, A., Palermo, V. & Falcone, C. PGK1, the gene encoding the glycolitic enzyme phosphoglycerate kinase, acts as a multicopy suppressor of apoptotic phenotypes in S. cerevisiae. *Yeast* **26**, 31–37 (2009).

24. Karlsson, R., Aspenström, P. & Byström, A. S. A Chicken β-Actin Gene Can Complement a Disruption of the Saccharomyces cerevisiae ACT1 Gene. *Molecular and Cellular Biology* **11**, 213–217 (1991).

25. Delgado, M. L. *et al.* The glyceraldehyde-3-phosphate dehydrogenase polypeptides encoded by the Saccharomyces cerevisiae TDH1, TDH2 and TDH3 genes are also cell wall proteins. *Microbiology* **147**, 411–417 (2001).

26. Schirmaier, F. & Philippsen, P. Identification of two genes coding for the translation elongation factor EF-1 alpha of S. cerevisiae. *EMBO J* **3**, 3311–3315 (1984).

27. Jauniaux, J.-C. & Grenson, M. GAP1, the general amino acid permease gene of Saccharomyces cerevisiae. *European Journal of Biochemistry* **190**, 39–44 (1990).

28. Ah Kang, H. *et al.* Characteristics of Saccharomyces cerevisiae gal1Δ and gal1Δhxk2Δ mutants expressing recombinant proteins from the GAL promoter. *Biotechnology and Bioengineering* **89**, 619–629 (2005).

29. de Smidt, O., du Preez, J. C. & Albertyn, J. The alcohol dehydrogenases of Saccharomyces cerevisiae: a comprehensive review. *FEMS Yeast Res* **8**, 967–978 (2008).

30. Teste, M.-A., Duquenne, M., François, J. M. & Parrou, J.-L. Validation of reference genes for quantitative expression analysis by real-time RT-PCR in Saccharomyces cerevisiae. *BMC Mol Biol* **10**, 99 (2009).

31. Rojas, V. & Larrondo, L. F. Coupling Cell Communication and Optogenetics: Implementation of a Light-Inducible Intercellular System in Yeast. *ACS Synth. Biol.* **12**, 71–82 (2023).

32. Zhao, E. M. *et al.* Optogenetic Amplification Circuits for Light-Induced Metabolic Control. *ACS Synth. Biol.* **10**, 1143–1154 (2021).

33. Le Bec, M. *et al.* Optogenetic spatial patterning of cooperation in yeast populations. *Nat Commun* **15**, 75 (2024).

34. Cleere, M. M. & Gardner, K. H. Optogenetic Control of Phosphate-Responsive Genes Using Single-Component Fusion Proteins in Saccharomyces cerevisiae. *ACS Synth. Biol.* **13**, 4085–4098 (2024).

35. Benzinger, D. & Khammash, M. Pulsatile inputs achieve tunable attenuation of gene expression variability and graded multi-gene regulation. *Nat Commun* **9**, 3521 (2018).

36. Zhao, E. M. *et al.* Optogenetic regulation of engineered cellular metabolism for microbial chemical production. *Nature* **555**, 683–687 (2018).

37. Pouzet, S. *et al.* Optogenetic control of beta-carotene bioproduction in yeast across multiple lab-scales. *Front. Bioeng. Biotechnol.* **11**, (2023).

38. Guerra, P., Vuillemenot, L.-A., Rae, B., Ladyhina, V. & Milias-Argeitis, A. Systematic In Vivo Characterization of Fluorescent Protein Maturation in Budding Yeast. *ACS Synth. Biol.* **11**, 1129–1141 (2022).
