# Supplementary material for: Dual-channel optogenetics in yeast for multiplexed light-based control of cellular processes and pathways
Source: Nat Commun. 2026 May 22;17:6742. doi: 10.1038/s41467-026-73399-0 (PMC13385801; doi:10.1038/s41467-026-73399-0)
Supplement: Supplementary file 2 — Description of Additional Supplementary Files [file 41467_2026_73399_MOESM2_ESM.pdf]

Description of Additional Supplementary Files

Supplementary Data 1: Excel sheet with sequences of all ORFs
