# Supplementary material for: Dual-channel optogenetics in yeast for multiplexed light-based control of cellular processes and pathways
Source: Nat Commun. 2026 May 22;17:6742. doi: 10.1038/s41467-026-73399-0 (PMC13385801; doi:10.1038/s41467-026-73399-0)
Supplement: Supplementary file 5 — Transparent Peer Review file [file 41467_2026_73399_MOESM5_ESM.pdf]

nature portfolio

Peer Review File

Dual-channel optogenetics in yeast for multiplexed light-based control of cellular processes and pathways

Corresponding Author: Dr Chueh Loo Poh

This file contains all reviewer reports in order by version, followed by all author rebuttals in order by version.

Version 0:

Reviewer comments:

Reviewer #1

(Remarks to the Author)
In this paper, the authors developed a red light responsive optogenetic system for S. cerevisiae. The system was refactored to work with the blue light responsive EL222 without crosstalk. Optogenetics is an attractive approach in synthetic biology and metabolic engineering, and the dual optogenetic system will provide useful insights that expand methods of cell engineering. The refactoring with a modular protein engineering approach for to improve the crosstalk with blue light is elegant. The advantage of using the orthologous optogenetic tools is clear for inducing the flocculation. But the advantage is not clear for the regulation of FNSI and F3'H expressions for flavonoid production in Fig. 5, so please add the explanation. My comments are listed below.

1. In Fig. 3b, it is clearly shown that the crosstalk has been eliminated in the y-iLightmA6LEXY. However, the mCitrine fluorescence was much lower than in the y-iLight under the 25% red light conditions. What is the reason for this?

2. It is preferable to vary the expression level stepwise with light intensity. To characterize this system, the response of the y-iLightmA6LEXY to red light intensity can be examined as shown in Fig. 2b.

3. In Fig. 4b, the mCitrine fluorescence of the OY-b.mA6LEXY was low under the red/blue conditions. If so, why did the luteolin production in Fig. 5 show effects even under the same duty cycles. Is this caused by the timing of the fluorescence observation? Time course monitoring of the fluorescence would provide useful insights.

4. The benefits of using dual optogenetic systems to control the luteolin synthesis pathway in Fig. 5 are not clearly described. Please explain clearly to help the reader understand. For example, is there a rational reason that the production of luteolin changes depending on whether it is via apigenin or eriodictyol? Do the enzymes have different Km values for each substrate?

5. In Fig. 5d, the time course data would help explain the effect of the light duty cycle.

6. The advantage of using the orthologous optogenetic tools is clear for inducing the flocculation in Fig. 6. In this strain, unlike Fig. 5, both FNSI and F3’H expressions are induced by blue light. The story is not connected to Fig. 5 and should be explained clearly to avoid misunderstanding.

7. L429-433: Luteolin does not seem to export form the cells well. If so, the same problem would have occurred in Fig. 5. The luteolin production should be evaluated by extracting the cells?

Minor comments
L71: Fig. 1a should be referred.

In Fig. 2b, the y-iLight shows a decrease in fluorescence intensity when the light is too strong. What is the reason for this?

In Fig. 5a, FNS should be shown FNSI.

Reviewer #2

(Remarks to the Author)
In this study, the authors combined red and blue light sensitive optogenetic elements to design a new red light optogenetic system. By modifying modular protein domains, the authors reduced blue light cross activation. Also, they integrated this system with the blue light one to create a dual channel strain and showed key applications in biomanufacturing and biomaterials. To some extent, this study is innovative, but there are still several issues that need to be addressed.
Major concern：
1. P6,Fig.2b,d：Did the author use SDS-PAGE or other methods to detect the expression of mCitrine fluorescent reporter under different red/blue light intensities?
2. Please check the uppercase or lowercase form of the constitutive PGK promoter in Figure 2b and Figure 2d. Keep labeling consistent throughout the manuscript.
3. Line 288：please confirm whether the labels are correct. According to the content, replace “Fig. 4b、c” with “Fig. 4a、b”.
4. In the section about the implementation of dual-channel optogenetics in a single strain, what to set the light treatment time at 60 minutes?
5. In lines 68-71, the authors detailed the information presented in Figure 1a. Consequently, the reference to “Fig. 1b” within parentheses should be amended to “Fig. 1a”.
6. In lines 278-280, the authors provided information about Figure 4a and 4b. Therefore, they should replace “Fig. 4b and c” within parentheses with “Fig. 4a and b”.
7. In lines 329-332, it appears that there is an inconsistency in the naming of the host strain used for luteolin bioproduction. Based on the context, it seems that the intended strain is OY-β. mA1LINX. Please check it.
8. In lines 337-339, the author should make corrections to "catalyzed by F3'H" to ensure grammatical accuracy and clarity.
9. To facilitate readers' understanding, the author should standardize the writing in the original text. For instance, the “600” in “OD600” should be subscripted, and strain names and gene names should be italicized.

Reviewer #3

(Remarks to the Author)
In the study ‘Dual-channel optogenetics in yeast for multiplexed light-based control of cellular processes and pathways’, Linus Yu Han Tan et al. present a multiplexing approach in Saccharomyces cerevisiae, combining a red and a blue light-responsive transcription factor. The red light system is based on the bacterial photoreceptor IsPadC BphP, which functions independently of exogenous cofactors. As a result, the system depends on only a single heterologous gene. It was originally developed in E. coli and mammalian cells and has now been successfully adapted and established in S. cerevisiae. The blue light-responsive transcription system used in this study was previously established in an earlier work and is based on EL222.
The authors demonstrate the potential of combining both systems in a dual-channel strain, although the red light system was unintentionally activated by blue light. While the authors successfully minimized blue-light cross-activation through protein engineering, this also led to a significant reduction in expression levels upon red light activation.
The functionality of this dual-channel strain is demonstrated in three applications:
1. Regulation of luteolin biosynthesis by the independent control of two enzymes using red and blue light.
2. Decoupling of the growth, production, and purification phase by blue light-induced bioproduction and red light-induced yeast flocculation for product isolation.
3. Spatial enzyme regulation using plated yeast that exhibits different color outputs depending on light induction.
This study presents a novel multiplexed light regulation system in yeast with promising applications. However, key limitations must be addressed to improve the scientific quality and impact of the manuscript.

Major Comments
• Figure Captions: All figure captions should be revised to include all relevant details on induction and cultivation conditions, proper strain names, measurement time points, and proper abbreviation explanations.
• Figure 2b: Reporter expression decreases at red light intensities above 25%. The authors should comment on possible reasons, as these effects are generally not known for red light induction. Why does expression regulated by CYC1 and PGK1 core promoters drop already beyond 10% activity, while the full lengths PGK1 promoter used for comparison was not effected? The authors should give reasons on why the core promoter has an effect on the light sensitivity. The authors should test if light pulses of the same intensity show the same effect. Further, OD values at different light intensities should be provided to clarify whether higher red light intensities impacted yeast growth.
• Figure 2d: Investigate why PGK1p-controlled expression decreases at blue light intensities above 25%. OD measurements should be provided to assess whether blue light exposure affects yeast cell growth. Additionally, why is this effect only observed for PGK1p and not for the light-regulated system?
• Characterization of y-iLight and EL222-based system: To fully characterize the newly developed light systems, the authors should properly analyze both strains under the optimal light conditions (25% red light, 25% blue light, or in darkness) over 48–72 hours, with measurements taken during the logarithmic, sationary and late stationary phases. Data should be shown in comparison to the native PGK1 promoter.
• Crosstalk analysis: iLight in mammalian systems did not exhibit blue light crosstalk. The authors should discuss potential differences between their yeast system and the mammalian system, considering light intensities used, architecture of constructs, and experimental setup. Did the authors attempt to minimize blue light intensity by applying light pulses to find the lowest level necessary for full activation of the blue light responsive system while reducing crosstalk with the red light system?

• Figure 3: The authors could eliminate the activation of the red light system upon blue light illumination through protein engineering. However, these modifications result in significantly lower expression levels of the target gene after red light induction. The authors should attempt to increase overall expression levels of the red light system by, for example, testing alternative activation domains, varying the number of lexO binding sites upstream of the core promoter, and exploring different promoters for driving the transcription factor.
• Figure 4 - Final Dual-Channel Strain Characterization: Strains were characterized based on reporter expression after illumination with diverse light settings at one timepoint, which is not specified in the caption or the main text. However, for the optimal light conditions defined from this experiment as well as in darkness, growth experiments and expression levels should be provided, covering the logarithmic, stationary, and late stationary phases. To evaluate the expression strengths, data should be shown in comparison to mTurquoise and mCitrine regulated by the native PGK1 promoter (as in Fig. 2).
• Figure 5: The conversion from either apigenin or eriodictyol to luteolin appears inefficient in all tested light conditions. The yields should be compared to those achieved with two constitutive promoters to determine whether the dual channel strain does not cause a limitation and prevent efficient product formation. It would be interesting to see if light induction is truly beneficial for bioproduction.
• Pathway control in luteolin production: The authors claim that the dual-channel system helps to identify the prefered enzymatic route for luteolin production.The study suggests that shifting the metabolic flux towards F3'H enhances luteolin production. To make this claim, protein or transcript levels need to be be investigated. The authors should confirm that higher F3'H levels resulting from the stronger blue light system did not cause higher intermediate and subsequent product levels. Protein levels and enzyme activity should be considered as possible impact on the production rate.
• Line 382: The authors state that they deactivated expression, but supporting data is not provided. This should be clarified. Deactivation performances of the y-iLight system via NIR would be an interesting aspect for the reader.
• Figure 6: The induction conditions are unclear. How long were cells grown in darkness to support growth without production (until what OD)? Did blue light induction stop after 24 hours, followed by red light illumantion only? Or was red light applied simultaneously with blue light? Figure 6a should specify the duration of cultivation in darkness, blue+NIR, and red light used in the experiment.
• Lines 436-438: The authors state that the flocculation in non-induced conditions might result from leaky FLO1 expression. However, leakiness of the system was not discussed before. What might be the cause of leakiness in FLO1 expression compared to the fluorescence reporter? Or was leakiness also observed for the fluorescence reporter?
• Citation of literature: The authors claim that the PhiReX system based on the photoreceptor from Arabidopsis thaliana requires four genes and a heterologous cofactor. However, as I understand, PhiReX can function with either two genes plus cofactor addition or four genes where the cofactor is endogenously expressed in yeast. Additionally, PhiReX has two versions: a tightly regulated system comparable to ADH1p levels and a more leaky version reaching around 2.5x ADH1p strength, which is comparable to y-iLight before engineering to prevent crosstalk. The authors should ensure correct citation and reference comparisons throughout the whole study.
• DNA sequences: The DNA sequences of the constructs used in this study should be made available.

Minor Comments
• Graphical Abstract: Lacks sufficient information to convey the study's content. I would suggest to include the figures from 1a and 1b and to indicate that the work was performed in yeast.
• Line 116-122: The episomal nature of the DBS-core-reporter cassette should be stated here. Further, the origin of replication and selection marker used in the plasmids should be indicated in Fig. 2a and b.
• The authors should carefully review the main text, as some sections are unclear or key information is missing. The following are some examples, but a thorough review of the entire manuscript is recommended:
o Line 163-164: Clarify why a lacO sequence was added downstream of the TATA box.
o Line 205: Fig. 3b is referenced as showing two time points, but only one is visible. Supplementary data include 8 h and 24 h measurements, but the rationale for these choices is unclear.
o What NLS sequences are used for mA1 and mA6?
o Line 248: Specify the 11 tested combinations.
o Line 280: References Fig. 4b and 4c, but Fig. 4c is missing—likely meant Fig. 4a and 4b.
o Lines 307-310, 376-378: Unclear phrasing.
o Lines 391-393: The authors claimed that products can be extracted cell-free from the culture media. The authors should clarify what they mean by cell-free in this context.
o Incomplete sentence at Line 494.
• Table 1: Absolute gene expression values should be provided.
• Experimental Replicates: The authors only mention biological replicates and no technical replicates. Please describe how the biological replicates were performed.
• Supplementary Information: Overall the text in Annex B should be condensed, with essential information incorporated into the main text. L. 12-22 and 67-76 are redundant with the main text.
• Figure 6: Explain why the blue-light-sensitive ODC degron remains fused to the FNS gene, as this construct should not produce luteolin upon blue light induction.
• Methods: Provide precise yeast transformation protocol.
• Line 636: The authors indicate that the starting OD was set to OD 1.5. If that is correct, the authors should state why they have chosen such a high starting OD.

Version 1:

Reviewer comments:

Reviewer #1

(Remarks to the Author)
I confirmed that the authors responded correctly to my comments.

Reviewer #2

(Remarks to the Author)
The current manuscript reported a light-controlled method to regulate gene expression in budding yeast. The research is very interesting and novel,providing a new method to investigate the function of genes in yeast.

Reviewer #3

(Remarks to the Author)
The authors have not adequately addressed the concerns raised in the first round of review. In their response letter, they fail to specify where revisions were made in the manuscript and, in several cases, refer to incorrect figures when answering specific questions.

A central unresolved issue concerns growth characterization. I repeatedly requested optical density (OD) measurements to evaluate whether the strains grow normally. Instead, the authors provide only fluorescence values normalized to OD, which do not allow conclusions about growth behavior. References to Figures 2c and 3c are misleading, as these panels do not contain OD data. The only OD measurements shown are in Supplementary Figure S1, which provides single end-point values after 24 h of cultivation. These values ($OD_{600} \approx 1.5$) are identical to the reported inoculation OD_{600}, making it unclear whether any growth occurred. Without growth curves, it is not possible to assess system performance or substantiate the claim that “there is no growth defect.” This lack of clarity remains a critical flaw.

The benchmarking strategy also raises concerns. In Figure 2d, the blue light system is compared against a PGK1 promoter construct. However, this reference promoter itself shows a pronounced drop in activity under constant light intensities above ~25%. By contrast, when light is applied in pulses (50% or 100% duty cycle), this reduction is not observed. These results suggest that constant blue light negatively impacts PGK1 activity, making it an unreliable comparator. Consequently, the authors cannot claim that their system surpasses the native PGK1 promoter under blue light conditions. To provide a valid benchmark and allow readers to assess the system’s performance, a promoter unaffected by blue light should be used.

Similarly, the reduced y-iLight activity at red-light intensities above 10% is attributed to steric hindrance within the promoter cassettes. This explanation highlights a design flaw that could, in principle, be improved through cassette optimization, yet no such optimization was attempted.

While the multiplexing concept is of interest, the overall performance of the system remains weak. The application examples reveal clear inefficiencies (e.g., poor conversion rates, leaky expression in the flocculation experiment), and no attempt was made to improve these shortcomings.

In its current form, the manuscript does not meet the quality standards required for a high-impact journal such as Nature Communications. Therefore, I cannot recommend acceptance.

Reviewer #4

(Remarks to the Author)
An ODE model was added to this manuscript in the review stage. The model captured light-induced gene expression and metabolic dynamics, using simple approximations: linear dependence of expression on light intensity, and Michaelis-Menten kinetics for the metabolic reactions.

The model is straightforward and generally sound. By using simple approximations, the number of fitted parameters is limited, leading to a decreased likelihood of overfitting. There are some potentially suboptimal aspects of the implementation, including the assumption that translation processes, RNA degradation processes, and DNA degradation processes all have the same first-order kinetic constants. Regardless of whether they should be first order, the fact that these processes are assumed to have the same constants even for the degradation/production of different proteins is unlikely to be precise. However, this is not a fatal flaw, and instead it only adds extra restrictions on the model that make it harder for the model to be accurate. This makes any accuracy in predictions actually more impressive. Of those three assumptions, the protein production rate is the least likely to be true and most likely to have an impact based on ribosome preferences for different transcripts.

The only significant concern is that the authors fixed the mRNA degradation rate. While they clearly indicated this in the supplement, there is no explanation of why (nor is there an obvious reason why), nor was this decision stated or described in the methods. This should be fixed.

Also of note, the submitted code has the actual parameter fitting function commented out and the "best fit" parameters hard-coded in. While straightforward for an interested user to adjust, this is not what one would consider to be a "best practice".

The fact that the model has reasonably good predictivity for test data on which it was not trained suggests that the model

reasonably accurately captures the biology, supporting the validity of analysis and interpretation of the kinetic parameters as the authors have done.

Version 2:

Reviewer comments:

Reviewer #3

(Remarks to the Author)
The authors have addressed all of my previous comments to a satisfactory extent.

Reviewer #4

(Remarks to the Author)
The authors have adequately addressed the critiques raised in the narrow scope of this reviewer's requested purview.

Open Access This Peer Review File is licensed under a Creative Commons Attribution 4.0 International License, which permits use, sharing, adaptation, distribution and reproduction in any medium or format, as long as you give appropriate credit to the original author(s) and the source, provide a link to the Creative Commons license, and indicate if changes were made.
In cases where reviewers are anonymous, credit should be given to 'Anonymous Referee' and the source.
The images or other third party material in this Peer Review File are included in the article's Creative Commons license, unless indicated otherwise in a credit line to the material. If material is not included in the article's Creative Commons license and your intended use is not permitted by statutory regulation or exceeds the permitted use, you will need to obtain permission directly from the copyright holder.
To view a copy of this license, visit https://creativecommons.org/licenses/by/4.0/

We greatly appreciate the comments and suggestions by the reviewers. Please find below our responses to the comments. The manuscript has been revised accordingly.

Reviewer #1 (Remarks to the Author):

In this paper, the authors developed a red light responsive optogenetic system for S. cerevisiae. The system was refactored to work with the blue light responsive EL222 without crosstalk. Optogenetics is an attractive approach in synthetic biology and metabolic engineering, and the dual optogenetic system will provide useful insights that expand methods of cell engineering. The refactoring with a modular protein engineering approach for to improve the crosstalk with blue light is elegant. The advantage of using the orthologous optogenetic tools is clear for inducing the flocculation. But the advantage is not clear for the regulation of FNSI and F3'H expressions for flavonoid production in Fig. 5, so please add the explanation. My comments are listed below.

1. In Fig. 3b, it is clearly shown that the crosstalk has been eliminated in the y-iLightmA6LEXY. However, the mCitrine fluorescence was much lower than in the y-iLight under the 25% red light conditions. What is the reason for this?

Primarily, the nuclear localization sequence of y-iLightmA6LEXY is mA6-NLS, which is significantly weaker than the SV40-NLS used in the original y-iLight. This reduces the amount of transcription factor that can directly access the DNA in the nucleus to initiate transcription, and this switch was necessary to match the activity of the LEXY blue light inducible nuclear export module used to eliminate crosstalk.

2. It is preferable to vary the expression level stepwise with light intensity. To characterize this system, the response of the y-iLightmA6LEXY to red light intensity can be examined as shown in Fig. 2b.

Additional experiments were conducted and included as Fig. 3f to characterize all 3 iLight variants with stepwise red light intensity.

3. In Fig. 4b, the mCitrine fluorescence of the OY-b.mA6LEXY was low under the red/blue conditions. If so, why did the luteolin production in Fig. 5 show effects even under the same duty cycles. Is this caused by the timing of the fluorescence observation? Time course monitoring of the fluorescence would provide useful insights.

The system used to produce luteolin was OY-b.mA1LINX, which had higher expression than OY-b.mA6LEXY. Even though expression was lower than the original iLight variant, this only reflects relative expression of the mCitrine marker. When applied to luteolin production, the level of enzyme expression was sufficient to catalyze the reaction.

Time course monitoring of the fluorescence of all the dual channel strains in both red and blue+NIR light across 56 hours was carried out and included as Annex C, Figures S4-6.

4. The benefits of using dual optogenetic systems to control the luteolin synthesis pathway in Fig. 5 are not clearly described. Please explain clearly to help the reader understand. For example, is there a rational reason that the production of luteolin changes depending on whether it is via apigenin or eriodictyol? Do the enzymes have different Km values for each substrate?

To clarify, the use of the luteolin pathway was only a case study to demonstrate the ability of the dual channel optogenetics to control metabolic pathways in a dose-dependent and time-resolved manner, as we had only shown our system to directly control protein expression in the earlier section of the paper. Optogenetics provides the opportunity to express genes in both a step-wise and time-resolved manner, and the dual channel optogenetics confers the added benefit of multiplexed control in the model industrial chassis *S. cerevisiae*, which allows for more complex pathways with multiple nodes to be controlled independently. Thus, it can be used as a tool to generate a variety of induction conditions to gain deeper insights into the behaviour of biosynthesis pathways that would have otherwise been difficult to ascertain using native constitutive or chemically inducible promoters. The motivation for this experiment was more clearly explained in the revised manuscript. In this case, the experiments in Figure 5 indicate that the F3'H enzyme is only functional in the early phase of cell growth, a bottleneck that would be difficult to discern with constitutive or chemically induced promoters that could not be dynamically switched between active or inactive states.

Concluding remarks of this section were rewritten to clearly express this point.

5. In Fig. 5d, the time course data would help explain the effect of the light duty cycle.

Time course data was obtained by repeating experiment and taking measurements at 6-, 12-, 18- and 24-hour time points and data is included in Fig. 5f and 5g.

6. The advantage of using the orthologous optogenetic tools is clear for inducing the flocculation in Fig. 6. In this strain, unlike Fig. 5, both FNSI and F3'H expressions are induced by blue light. The story is not connected to Fig. 5 and should be explained clearly to avoid misunderstanding.

Additional context was added to the paragraph to better link it to the previous result.

7. L429-433: Luteolin does not seem to export form the cells well. If so, the same problem would have occurred in Fig. 5. The luteolin production should be evaluated by extracting the cells?

In Figure 5, the luteolin is extracted from homogeneous cell culture, which contains both the media and the cells which are lysed by the ethanol extraction method. This only becomes an issue when considering flocculation as the cells are disproportionately present in the bottom portion of the volume.

Minor comments
L71: Fig. 1a should be referred.

One of the references to Fig. 1b was a typo, it was amended to refer to Fig. 1a

In Fig. 2b, the y-iLight shows a decrease in fluorescence intensity when the light is too strong. What is the reason for this?
One possible explanation for the non-linear behaviour of the red-light promoter could be the steric hinderance of active y-iLight preventing other transcription factors and transcription elements from binding[1], as binding of transcription activators near to the core promoter has been shown to decrease expression due to interfering with the RNA polymerase binding[2]. Another possible mechanism would be 'facilitated diffusion', which occurs when due to crowding of the transcription factor on the DNA strand, and while rate of initial binding increases, residence time for transcription factors decreases, resulting in poor expression from promoter[3,4]. Both of these proposed mechanisms would manifest as lower expression from a promoter in the presence of high concentrations of active transcription factors.

In Fig. 5a, FNS should be shown FNSI.

Figure amended.

Reviewer #2 (Remarks to the Author):

In this study, the authors combined red and blue light sensitive optogenetic elements to design a new red light optogenetic system. By modifying modular protein domains, the authors reduced blue light cross activation. Also, they integrated this system with the blue light one to create a dual channel strain and showed key applications in biomanufacturing and biomaterials. To some extent, this study is innovative, but there are still several issues that need to be addressed.
Major concern：
1. P6,Fig.2b,d：Did the author use SDS-PAGE or other methods to detect the expression of mCitrine fluorescent reporter under different red/blue light intensities?

Thank you for the suggestion, SDS-PAGE was not used but we have included additional RT-qPCR data to demonstrate additional evidence of the effect of red and blue light cycles on expression of genes in Fig. 5d and e.

2. Please check the uppercase or lowercase form of the constitutive PGK promoter in Figure 2b and Figure 2d. Keep labeling consistent throughout the manuscript.

Promoter labels were reworked to abide by standard yeast conventions, *PGK* promoter will be changed to P_{PGK1}

3. Line 288：please confirm whether the labels are correct. According to the content, replace “Fig. 4b、c” with “Fig. 4a、b”.

The labels have been amended to the correct labelling.

4. In the section about the implementation of dual-channel optogenetics in a single strain, what to set the light treatment time at 60 minutes?

60 minutes was chosen as a timeframe that could reasonably allow for transcription and translation to take place for a particular given channel, but not too long that protein products would degrade. This is to have a condition where both channels could be active.

15 minute and 30 minute cycles were tested as well, the 60 minute cycle provided better results for the red light channel so this was chosen. This data has been added to the supplementary materials in Annex F.

5. In lines 68-71, the authors detailed the information presented in Figure 1a. Consequently, the reference to “Fig. 1b” within parentheses should be amended to “Fig. 1a”.

Parentheses have been amended.

6. In lines 278-280, the authors provided information about Figure 4a and 4b. Therefore, they should replace “Fig. 4b and c” within parentheses with “Fig. 4a and b”.
7. In lines 329-332, it appears that there is an inconsistency in the naming of the host strain used for luteolin bioproduction. Based on the context, it seems that the intended strain is OY-β. mA1LINX. Please check it.

Amended naming error.

8. In lines 337-339, the author should make corrections to "catalyzed by F3'H" to ensure grammatical accuracy and clarity.

Edited grammar in section.

9. To facilitate readers' understanding, the author should standardize the writing in the original text. For instance, the “600” in “OD600” should be subscripted, and strain names and gene names should be italicized.

Gene and promoter nomenclature was standardized to yeast naming conventions, OD_{600} was subscripted. Changes in the manuscript have been made accordingly.

Reviewer #3 (Remarks to the Author):

In the study ‘Dual-channel optogenetics in yeast for multiplexed light-based control of cellular processes and pathways’, Linus Yu Han Tan et al. present a multiplexing approach in Saccharomyces cerevisiae, combining a red and a blue light-responsive transcription factor. The red light system is based on the bacterial photoreceptor IsPadC BphP, which functions independently of exogenous cofactors. As a result, the system depends on only a single heterologous gene. It was originally developed in E. coli and mammalian cells and has now been successfully adapted and established in S. cerevisiae. The blue light-responsive transcription system used in this study was previously established in an earlier work and is based on EL222.
The authors demonstrate the potential of combining both systems in a dual-channel strain, although the red light system was unintentionally activated by blue light. While the authors successfully minimized blue-light cross-activation through protein engineering, this also led to a significant reduction in expression levels upon red light activation.
The functionality of this dual-channel strain is demonstrated in three applications:
1. Regulation of luteolin biosynthesis by the independent control of two enzymes using red and blue light.
2. Decoupling of the growth, production, and purification phase by blue light-induced bioproduction and red light-induced yeast flocculation for product isolation.
3. Spatial enzyme regulation using plated yeast that exhibits different color outputs depending on light induction.
This study presents a novel multiplexed light regulation system in yeast with promising applications. However, key limitations must be addressed to improve the scientific quality and impact of the manuscript.

Major Comments
• Figure Captions: All figure captions should be revised to include all relevant details on induction and cultivation conditions, proper strain names, measurement time points, and proper abbreviation explanations.

Thank you for the suggestions. Figure captions have been revised to reflect all the above considerations.

• Figure 2b: Reporter expression decreases at red light intensities above 25%. The authors should comment on possible reasons, as these effects are generally not known for red light induction. Why does expression regulated by CYC1 and PGK1 core promoters drop already beyond 10% activity, while the full lengths PGK1 promoter used for comparison was not effected? The authors should give reasons on why the core promoter has an effect on the light sensitivity. The authors should test if light pulses of the same intensity show the same effect. Further, OD values at different light intensities should be provided to clarify whether higher red light intensities impacted yeast growth.

One possible explanation for the non-linear behaviour of the red-light promoter could be the steric hinderance of active y-iLight preventing other transcription factors and transcription elements from binding[1], as binding of transcription activators near to the core promoter has been shown to decrease expression due to interfering with the RNA polymerase binding[2]. Another possible mechanism would be 'facilitated diffusion', which occurs when due to crowding of the transcription factor on the DNA strand, and while rate of initial binding increases, residence time for transcription factors decreases, resulting in poor expression from promoter[3,4]. Both of these proposed mechanisms would manifest as lower expression from a promoter in the presence of high concentrations of active transcription factors.

The different core promoters resulted in different levels of reporter expression, and it was noted that the exposure to red light intensity beyond 10% for $P_{lexCYC1}$ and $P_{lexPGK1}$ resulted in a drop in y-iLight activity, while for $P_{lexENO1}$ red light intensity greater than 50% resulted in activity. Because this non-linear behaviour is present for all three core promoters, it is likely an artefact of the y-iLight transcription factor itself. The three promoters may have differing sensitivities to light as core promoters contain several elements including the binding site for RNA polymerase, the transcription start site as well as the region of DNA that the post-initiation complex binds to[5], and the affinity and activity of these elements are dependent on core promoter sequences. Different core promoters have been shown to vary the overall activity of the promoter[6], as well as impact the sensitivity of promoters to trans-acting elements[7]. Thus, while not directly affected by light levels, the transcriptional elements that are recruited to a given core promoter may react differently to any given level of y-iLight activation, resulting in differing strengths and behaviour. This explanation has been included in the revised manuscript.

Light pulses of 30 minutes of 50%/100% intensity of red light followed by 30 minutes of darkness were carried out and included in the characterization in Figure 2b. Light cycles of red light were able to restore expression in all systems. However, expression did not exceed that of 25% with the P-lexENO promoter, so this condition was maintained as the standard going forward.

OD_{600} measurements taken to normalize the fluorescence were included in Figure 2c, with no observable increases or decreases in growth for any of the conditions.

• Figure 2d: Investigate why PGK1p-controlled expression decreases at blue light intensities above 25%. OD measurements should be provided to assess whether blue light exposure affects yeast cell growth. Additionally, why is this effect only observed for PGK1p and not for the light-regulated system?

OD_{600} measurements taken to normalize the fluorescence were included in Figure 3c. There were no observable increases or decreases in growth for any of the conditions. Explanation for loss of activity of P_{PGK1} was rewritten to explain how it was possible that only the *PGK* promoter was affected and not the *6CCL* promoter.

It has been shown that blue light may reduce oxygen uptake and respiration in yeasts without affecting viability[8], and may occur through damaging the mitochondrial complex[9]. As the PGK1 enzyme is heavily involved in glycolysis, the *PGK1* promoter may be downregulated due to a general impact of blue light on respiration. Alternatively, blue light has been shown to activate a stress response through the Msn2 protein[10], localizing the protein to the cytoplasm. As Msn2p is a key activator of glycolytic enzymes including PGK1[11], this may result in a downregulation specifically of the *PGK1* promoter without affecting P_{6CCL}.

• Characterization of y-iLight and EL222-based system: To fully characterize the newly developed light systems, the authors should properly analyze both strains under the optimal light conditions (25% red light, 25% blue light, or in darkness) over 48–72 hours, with measurements taken during the logarithmic, stationary and late stationary phases. Data should be shown in comparison to the native PGK1 promoter.

Thank you for the suggestion. Additional experiments have been performed. y-iLight and EL222 were tested in their optimal light conditions over a period of 56 hours, with measurements of fluorescent reporter expression taken either in the exponential phase(0-8 hours), stationary phase(24-32 hours) or late stationary phase(48-56 hours) at 2-hour intervals. P_{PGK1} expressing the corresponding fluorescent reporter was cultured and measured alongside optogenetic cells. The data is shown in Annex C in Supplementary figure S2 and S3. The results demonstrate an increase in RFU across exponential phase, a steady level of expression in the stationary phase and decreased expression in the late stationary phase, likely due to exhaustion of carbon source by this stage.

• Crosstalk analysis: iLight in mammalian systems did not exhibit blue light crosstalk. The authors should discuss potential differences between their yeast system and the mammalian system, considering light intensities used, architecture of constructs, and experimental setup. Did the authors attempt to minimize blue light intensity by applying light pulses to find the lowest level necessary for full activation of the blue light responsive system while reducing crosstalk with the red light system?

The iLight system that was developed in mammalian cells did not exhibit any crosstalk under the conditions tested in that paper, but this may stem from their use of shorter, second-long light pulses as well as testing with a blue-green light of 505 nm wavelength instead of a blue 465 nm wavelength[1]. In addition, the amount of biliverdin present differs greatly from mammalian to *S.cerevisiae* cells, as mammalian cells break down biliverdin through biliverdin reductase[1] while *S.cerevisiae* lacks this enzyme[1]. This may cause an increased build-up in biliverdin in *S.cerevisiae*, increasing its sensitivity to light.

Decreasing intensities of light were examined to see if they would result in less crosstalk. While lower intensities of blue light reduced cross-activation of iLight, they would similarly reduce expression from EL222, and thus it was an unviable strategy. Data for this has been included in Annex D in the supplementary material.

• Figure 3: The authors could eliminate the activation of the red light system upon blue light illumination through protein engineering. However, these modifications result in significantly lower expression levels of the target gene after red light induction. The authors should attempt to increase overall expression levels of the red light system by, for example, testing alternative activation domains, varying the number of lexO binding sites upstream of the core promoter, and exploring different promoters for driving the transcription factor.

Thank you for the comment. The main focus of our study was achieving multiplexed optogenetics and showing how the increased dynamic control can be applied in synthetic biology, as well as engineering of the novel red light system and reducing crosstalk levels. We acknowledge that the further tuning of the system to increase its expression would improve it but it is beyond the scope of this study as the levels of expression obtained were sufficient to execute the proofs of concept that we implemented.

• Figure 4 - Final Dual-Channel Strain Characterization: Strains were characterized based on reporter expression after illumination with diverse light settings at one timepoint, which is not specified in the caption or the main text. However, for the optimal light conditions defined from this experiment as well as in darkness, growth experiments and expression levels should be provided, covering the logarithmic, stationary, and late stationary phases. To evaluate the expression strengths, data should be shown in comparison to mTurquoise and mCitrine regulated by the native PGK1 promoter (as in Fig. 2).

Timepoint for measurement was included for the diverse light settings, and measurements across 56 hours similar to that for single channel strains was carried out and included in Annex C Figures S4-6.

• Figure 5: The conversion from either apigenin or eriodictyol to luteolin appears inefficient in all tested light conditions. The yields should be compared to those achieved with two constitutive promoters to determine whether the dual channel strain does not cause a limitation and prevent efficient product formation. It would be interesting to see if light induction is truly beneficial for bioproduction.

Thank you for your suggestion. To clarify, the main purpose of this part of the study was to study the ability of optogenetics to direct metabolic flux between two different pathways, and thus we did not put emphasis on engineering maximal levels of luteolin for bioproduction. We were envisioning optogenetics as a tool to be able to better control complex metabolic pathways to gain understanding. Optogenetics provides the opportunity to express genes in both a step-wise and time-resolved manner, and the dual channel optogenetics confers the added benefit of multiplexed control in the model industrial chassis *S. cerevisiae*, which allows for more complex pathways with multiple nodes to be controlled independently. Thus, it can be used as a tool to generate a variety of induction conditions to gain deeper insights into the behaviour of biosynthesis pathways that would have otherwise been difficult to ascertain using native constitutive or chemically inducible promoters. The motivation for this

experiment was more clearly explained in the revised manuscript. To expand on the potential for optogenetics as a tool to probe the behaviour of metabolic pathways and further capitalize on the data generated, we demonstrated that the data generated could be used to fit a kinetic model of the pathways to gain insights. This enabled differential reaction kinetics between the two pathways to be analyzed based on the estimated model parameters. A section on the modelling insights have been added in the revised manuscript.

• Pathway control in luteolin production: The authors claim that the dual-channel system helps to identify the preferred enzymatic route for luteolin production. The study suggests that shifting the metabolic flux towards F3’H enhances luteolin production. To make this claim, protein or transcript levels need to be investigated. The authors should confirm that higher F3’H levels resulting from the stronger blue light system did not cause higher intermediate and subsequent product levels. Protein levels and enzyme activity should be considered as possible impact on the production rate.

Thank you for the comment. We apologise for the confusion. To clarify, we were not claiming that substrate would preferentially be funnelled through one pathway over the other due to substrate affinity, but rather better yield is obtained if F3’H enzyme is actively expressed more to increase the amount of substrate processed by the F3’H, and thus is preferential for the end user. We carried out the experiments with the hypothesis that protein levels would be the driving factor in the changes in production rate, to reflect the variety of expression patterns we can achieve with optogenetics. We agree that transcript data would be useful, and RT-qPCR data was generated and included in Figure 5e and f to show the shift in enzyme expression with different light cycles.

• Line 382: The authors state that they deactivated expression, but supporting data is not provided. This should be clarified. Deactivation performances of the y-iLight system via NIR would be an interesting aspect for the reader.

Thank you for the suggestion. Experiments were carried out to demonstrate the effect of NIR light. BY474R was exposed to red light for 2 hours, and exposed to either NIR light, further red light or darkness for another 3 hours, and time point measurements of the RFU of mCitrine reporter were taken. These showed that NIR could lower expression of mCitrine faster than keeping the cells in darkness, and results were included in Figure 3.

• Figure 6: The induction conditions are unclear. How long were cells grown in darkness to support growth without production (until what OD)? Did blue light induction stop after 24 hours, followed by red light illumantion only? Or was red light applied simultaneously with blue light? Figure 6a should specify the duration of cultivation in darkness, blue+NIR, and red light used in the experiment.

Cells were grown overnight to reach an OD_{600} of 15-20 before dilution to OD_{600}=1.5 for induction. Blue light was stopped when red light illumination began. Clarification was added

to the results section, and the diagram changed to reflect the duration of the different phases in the revised manuscript.

• Lines 436-438: The authors state that the flocculation in non-induced conditions might result from leaky FLO1 expression. However, leakiness of the system was not discussed before. What might be the cause of leakiness in FLO1 expression compared to the fluorescence reporter? Or was leakiness also observed for the fluorescence reporter?

To clarify, leakiness observed from the fluorescent reporter would be the RFU observed for the cells kept in the dark state. All inducible promoters have some relative levels of leakiness, although it is difficult to infer how it will translate to protein activity. In this case, the levels of leakiness observed through the fluorescent reporter are significantly less than the expression when exposed to light, but when translated to FLO1 proteins they are sufficient to induce some level of flocculation.

• Citation of literature: The authors claim that the PhiReX system based on the photoreceptor from Arabidopsis thaliana requires four genes and a heterologous cofactor. However, as I understand, PhiReX can function with either two genes plus cofactor addition or four genes where the cofactor is endogenously expressed in yeast. Additionally, PhiReX has two versions: a tightly regulated system comparable to ADH1p levels and a more leaky version reaching around 2.5x ADH1p strength, which is comparable to y-iLight before engineering to prevent crosstalk. The authors should ensure correct citation and reference comparisons throughout the whole study.

Thank you for pointing this out. References and comparisons to both PhiReXs were added to the discussion, and phrasing was edited to more accurately reflect the number of genes needed for PhiReX.

• DNA sequences: The DNA sequences of the constructs used in this study should be made available.

DNA sequences of all relevant constructs that were used in this study were added to Annex A

Minor Comments

• Graphical Abstract: Lacks sufficient information to convey the study's content. I would suggest to include the figures from 1a and 1b and to indicate that the work was performed in yeast.

Graphical abstract has been revised to reflect the subject of multiplexed optogenetics in yeast.

• Line 116-122: The episomal nature of the DBS-core-reporter cassette should be stated here. Further, the origin of replication and selection marker used in the plasmids should be indicated in Fig. 2a and b.

Sentence amended for clarity. The origin of replication and selection markers were added to all plasmid diagrams

• The authors should carefully review the main text, as some sections are unclear or key information is missing. The following are some examples, but a thorough review of the entire manuscript is recommended:
o Line 163-164: Clarify why a lacO sequence was added downstream of the TATA box.

This sequence was inherited from another body of work which was not used in this particular study, but we think it was important to highlight this feature for completeness as this would preclude the use of any other lacI based proteins in conjunction with the system.

o Line 205: Fig. 3b is referenced as showing two time points, but only one is visible. Supplementary data include 8 h and 24 h measurements, but the rationale for these choices is unclear.

Fig. 3b reference was amended, originally both 8 h and 24 h measurements were to be shown in the main paper but the 8 h measurements were moved to the supplementary materials to maintain brevity. The purpose of measuring both 8 h and 24 h was to ensure that the crosstalk eliminating measures maintained effectiveness during both the exponential and stationary phase.

o What NLS sequences are used for mA1 and mA6?

NLS sequences were included in full gene sequences added to the supplementary materials in Annex A

o Line 248: Specify the 11 tested combinations.

Added table to specify the combinations tested

o Line 280: References Fig. 4b and 4c, but Fig. 4c is missing—likely meant Fig. 4a and 4b.

The reference has been fixed.

o Lines 307-310, 376-378: Unclear phrasing.

These lines have been rewritten along with the section for clearer phrasing and more focused discussion.

o Lines 391-393: The authors claimed that products can be extracted cell-free from the culture media. The authors should clarify what they mean by cell-free in this context.

Phrasing was changed to culture media separate from the cells
o Incomplete sentence at Line 494.

Sentence rewritten
• Table 1: Absolute gene expression values should be provided.

Absolute gene expression for red light and crosstalk eliminating condition have been added to table 2

• Experimental Replicates: The authors only mention biological replicates and no technical replicates. Please describe how the biological replicates were performed.

Biological replicates were separate instances of strains inoculated from glycerol stocks, information on technical replicates was added to the methods section.

• Supplementary Information: Overall the text in Annex B should be condensed, with essential information incorporated into the main text. L. 12-22 and 67-76 are redundant with the main text.

Explanation on the swapping of NLS was incorporated into the revised main text, explanation of the iterative process was removed and replaced with just the metrics for crosstalk measurement and the results of different variants.

• Figure 6: Explain why the blue-light-sensitive ODC degron remains fused to the FNS gene, as this construct should not produce luteolin upon blue light induction.

This degron is not blue light sensitive, it is only the destabilizing C-terminal tag, used to decrease the default leaky activity of the FNSI gene so that activity would accurately reflect the activity of the channel.

• Methods: Provide precise yeast transformation protocol.

Yeast transformation protocol added to methods

• Line 636: The authors indicate that the starting OD was set to OD 1.5. If that is correct, the authors should state why they have chosen such a high starting OD.

For yeast, OD_{600} 1.5 is considered a standard OD_{600} for starting cultures, after 2 days of inoculation from glycerol stocks or colonies they will reach OD_{600} =~15-20, and are diluted by 10x to refresh the culture such that exponential phase will occur over the next 8 hours, and stationary phase is reached around 20-24 hours.

References

1. van Dijk, D. *et al.* Large-scale mapping of gene regulatory logic reveals context-dependent repression by transcriptional activators. *Genome Res.* **27**, 87–94 (2017).
2. Yasmeen, E., Wang, J., Riaz, M., Zhang, L. & Zuo, K. Designing artificial synthetic promoters for accurate, smart, and versatile gene expression in plants. *Plant Commun.* **4**, 100558 (2023).
3. Koşar, Z. & Erbaş, A. Can the Concentration of a Transcription Factor Affect Gene Expression? *Front. Soft Matter* **2**, (2022).
4. Zabet, N. R. & Adryan, B. The effects of transcription factor competition on gene regulation. *Front. Genet.* **4**, 197 (2013).
5. Hubmann, G., Thevelein, J. M. & Nevoigt, E. Natural and Modified Promoters for Tailored Metabolic Engineering of the Yeast Saccharomyces cerevisiae. in *Yeast Metabolic Engineering* 17–42 (Humana Press, New York, NY, 2014). doi:10.1007/978-1-4939-0563-8_2.
6. Lubliner, S. *et al.* Core promoter sequence in yeast is a major determinant of expression level. *Genome Res.* **25**, 1008–1017 (2015).
7. Rantasalo, A., Kuivanen, J., Penttilä, M., Jäntti, J. & Mojzita, D. Synthetic Toolkit for Complex Genetic Circuit Engineering in Saccharomyces cerevisiae. *ACS Synth. Biol.* **7**, 1573–1587 (2018).
8. Ninnemann, H., Butler, W. L. & Epel, B. L. Inhibition of respiration in yeast by light. *Biochim. Biophys. Acta BBA - Bioenerg.* **205**, 499–506 (1970).
9. Molin, M. *et al.* Protein kinase A controls yeast growth in visible light. *BMC Biol.* **18**, 168 (2020).
10. Camponeschi, I., Montanari, A., Mazzoni, C. & Bianchi, M. M. Light Stress in Yeasts: Signaling and Responses in Creatures of the Night. *Int. J. Mol. Sci.* **24**, 6929 (2023).

11. Kuang, Z., Pinglay, S., Ji, H. & Boeke, J. D. Msn2/4 regulate expression of glycolytic enzymes and control transition from quiescence to growth. *eLife* **6**, e29938.

We appreciate the comments by the reviewers and our apologies for not being clear in the earlier revised manuscript. We have addressed the comments raised and additional experiments have been performed. Please find below our responses to the comments. The manuscript has been revised accordingly. A version of the revised manuscript with changes highlighted in yellow has also been submitted for reference.

Reviewer 3:

Comments: The authors have not adequately addressed the concerns raised in the first round of review. In their response letter, they fail to specify where revisions were made in the manuscript and, in several cases, refer to incorrect figures when answering specific questions.

A central unresolved issue concerns growth characterization. I repeatedly requested optical density (OD) measurements to evaluate whether the strains grow normally. Instead, the authors provide only fluorescence values normalized to OD, which do not allow conclusions about growth behavior. References to Figures 2c and 3c are misleading, as these panels do not contain OD data. The only OD measurements shown are in Supplementary Figure S1, which provides single end-point values after 24 h of cultivation. These values (OD600 ≈ 1.5) are identical to the reported inoculation OD600, making it unclear whether any growth occurred. Without growth curves, it is not possible to assess system performance or substantiate the claim that “there is no growth defect.” This lack of clarity remains a critical flaw.

Reply: We acknowledge the OD600 results found in the supplementary figures were misleading. They appeared to be similar to the starting OD600 of experiments because they were measured using a microplate reader, whereas the OD600 mentioned in the methodology in the main manuscript was measured using a standard cuvette. To rectify this, the supplementary figures were edited to reflect OD600 measured using a cuvette, and OD600 values were measured over 10 hours to construct a growth curve. Supplementary Figure S1 has been revised using the new results (as shown below). The results indicated that the presence of blue or red light in low or high intensities did not cause any observable growth defect. Similarly, cycles of 30 minutes high intensity light and 30 minutes darkness did not cause any change in OD600.

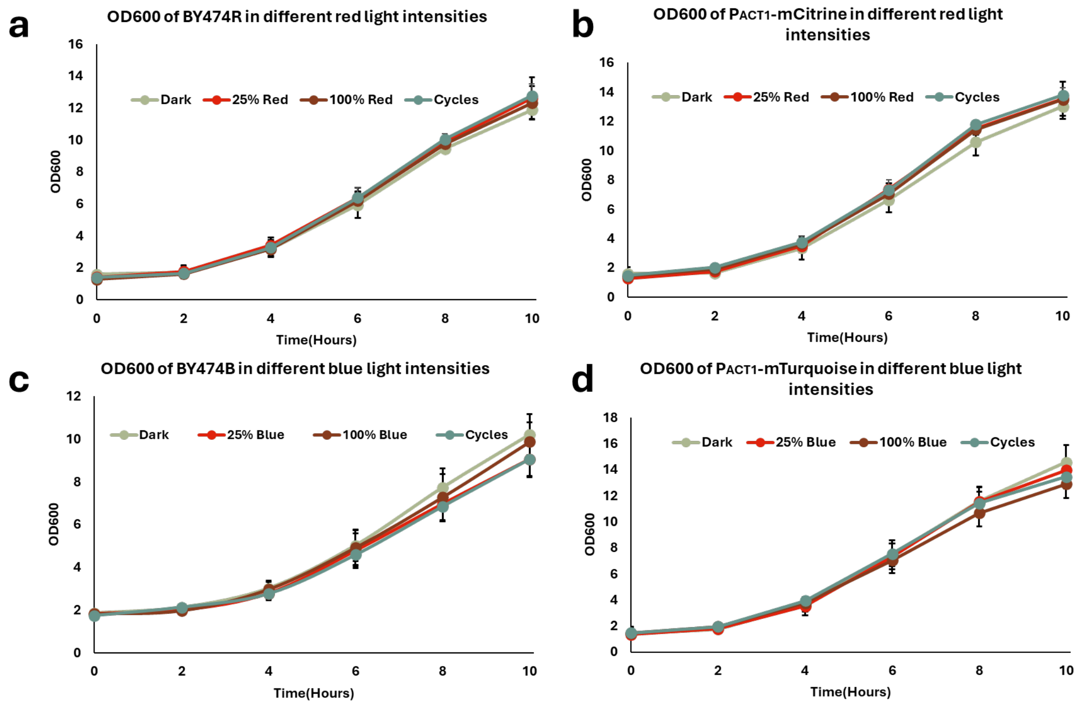

Supplementary Fig. S1: **a** OD_{600} values of strain BY474R across 10 hours after culturing in darkness, 25% red light, 100% red light and cycles of 30 minutes of 100% red light followed by 30 minutes of darkness. Values are an average of 3 biological replicates. Error bars indicate standard deviation with a sample size of n=3. **b** OD_{600} values of BY4741 expressing mCitrine reporter from native promoter P_{ACT1} across 10 hours after culturing in darkness, 25% red light, 100% red light and cycles of 30 minutes of 100% red light followed by 30 minutes of darkness. Values are an average of 3 biological replicates. Error bars indicate standard deviation with a sample size of n=3. **c** OD_{600} values of strain BY474B across 10 hours after culturing in darkness, 25% blue light, 100% blue light and cycles of 30 minutes of 100% blue light followed by 30 minutes of darkness. Values are an average of 3 biological replicates. Error bars indicate standard deviation with a sample size of n=3. **d** OD_{600} values of BY4741 expressing mTurquoise reporter from native promoter P_{ACT1} across 10 hours after culturing in darkness, 25% blue light, 100% blue light and cycles of 30 minutes of 100% blue light followed by 30 minutes of darkness. Values are an average of 3 biological replicates. Error bars indicate standard deviation with a sample size of n=3.

The benchmarking strategy also raises concerns. In Figure 2d, the blue light system is compared against a PGK1 promoter construct. However, this reference promoter itself shows a pronounced drop in activity under constant light intensities above ~25%. By contrast, when light is applied in pulses (50% or 100% duty cycle), this reduction is not observed. These results suggest that constant blue light negatively impacts PGK1 activity, making it an unreliable comparator. Consequently, the authors cannot claim that their system surpasses the native PGK1 promoter under blue light conditions. To provide a valid benchmark and allow

readers to assess the system's performance, a promoter unaffected by blue light should be used.

Reply: We acknowledge that the constitutive promoter P-PGK1 was affected by the high blue light intensities. To clarify, our intent is to assess whether our system has strength comparable to other commonly used promoters (i.e., strong enough to carry out similar functions), and not to claim that our system is superior to native promoters such as PGK1 in all conditions.

Nonetheless, we noted that a native constitutive promoter that will not be affected by high blue light will be required to compare the promoter strength across all the light conditions tested.

We first tested another native constitutive promoter P-ACT1 under 25%, 50% and 100%. Similarly, it showed that an increase in blue light intensity resulted in a decrease in expression at 100%, which was alleviated through the use of duty cycles (Supplementary Figure S21).

Hence, we further identified additional 5 constitutive native promoters from various aspects of the *S.cerevisiae* metabolism and experiments were performed to study their expression under various high blue light intensities. We chose the promoters P-TDH3, P-TEF1, P-ADH1, P-GAP1 and P-GAL1 (Table S6), with the hypothesis that since growth was unaffected (Supplementary Fig. S1), we hypothesize that these key promoters responsible for various aspects of the *S. cerevisiae* metabolism should remain stable. The expression of these promoters were tested in dark, 50% and 100% blue light conditions over 24 hours (Supplementary Fig. S22). Unexpectedly, the expression of all promoters had decreased with increase intensity of blue light. This suggests that while this phenomenon was not severe enough to critically affect growth, it was generic enough to impact multiple aspects of *S. cerevisiae* metabolism. We decided to focus on P-ACT1 as it showed the least drop off under high intensity blue light.

Table S6: Table of promoters tested for blue light sensitivity and their respective function in cell metabolism.

Promoter	Purpose
PGK1	Glycolysis and gluconeogenesis[1]
ACT1	Involved in cell polarization and cytoskeleton[2]
TDH3	Glycolysis and gluconeogenesis[3]
TEF1	Translation and tRNA binding[4]
GAP1	Amino acid uptake[5]
GAL1	Galactose metabolism[6]
ADH1	Ethanol metabolism[7]

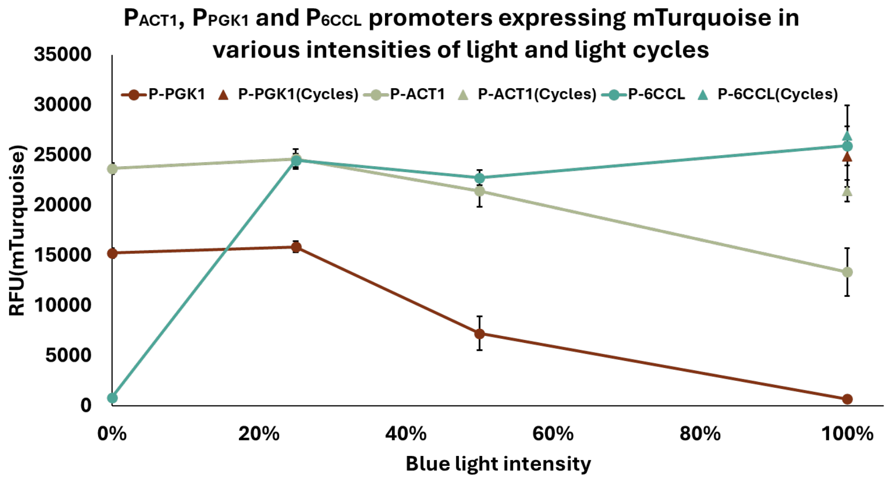

Supplementary Fig. S21: Expression of mTurquoise fluorescent reporter under P_{6CCL}, P_{ACT1} and P_{PGK1} in 0-100% blue light. Cells were cultured at 30°C in a shaking incubator while exposed to 0%-100% blue light (See Supplementary Fig. S19 for operational values of light intensity), as well as cycles of 30 minutes of 100% blue light followed by 30 minutes of darkness (Represented by triangle data points). Fluorescence was measured at 24 hours and normalized to OD_{600}. Values are an average of 3 biological replicates. Error bars indicate standard deviation with a sample size of n=3.

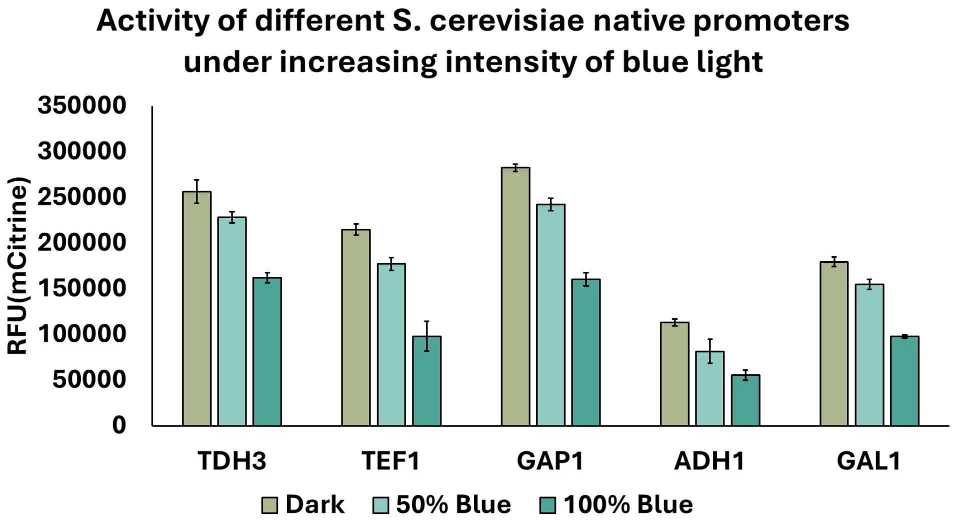

Supplementary Fig. S22: Activity of various constitutive native *S. cerevisiae* promoters in increasing intensities of blue light. Cells expressing mCitrine fluorescent protein from the listed constitutive promoters were cultured at 30°C in a shaking incubator while exposed to darkness, 50% or 100% blue light. Cells harbouring the GAL1 promoter were cultured in 2% galactose, 0.2% glucose YNB media, all other cells were cultured in 2% glucose YNB media. Fluorescence was measured at 24 hours and normalized to OD_{600}. Values are an average of 3 biological replicates. Error bars indicate standard deviation with a sample size of n=3.

To probe this further, we wanted to find out if this general effect was due to the blue light affecting the transcription process itself. Thus, we conducted RT-qPCR experiments to measure the levels of the mRNA transcripts from our blue light promoter as well as the native

promoter P-ACT1. We found that blue light resulted in a decrease in transcript levels by the native constitutive promoter (P-ACT1), but not from our blue light promoter (Supplementary Figure S23). This suggests that the high intensities of blue light affected some aspects of the transcription. In addition, this effect was not felt by the EL222 driven P_{6CCL} promoter. However, further examining the effect is out of scope the study.

As such, we rephrased our claim in the revised manuscript to only compare the expression level at blue light levels lower than 50%, and changed the reference promoter to P-ACT1 which showed the least decrease under high blue light. To be consistent, additional experiments have been performed to include the 56-hour characterization results of P-ACT1 across exponential, stationary and late stationary phase in revised supplementary figures S2-S6. Similar trends as P-PGK1 were observed and did not affect our conclusion.

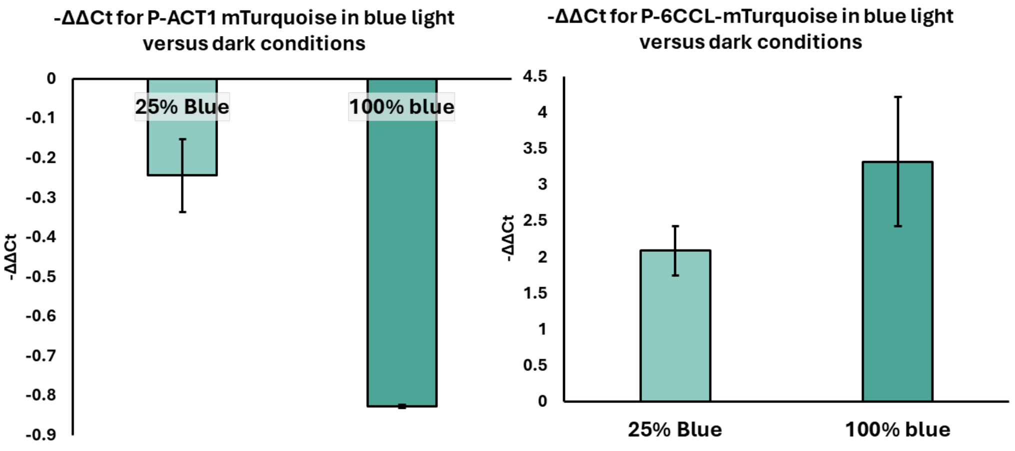

Supplementary Fig. S23: RT-qPCR results for BY474B and BY4741 expressing mTurquoise from P_{ACT1} in blue light compared to darkness. a -ΔΔCt for cells expressing mTurquoise under the control of P_{ACT1} promoter. Cycling times for mTurquoise RNA was compared against native housekeeping gene UBC6, and conditions in 25% or 100% blue light were compared to dark conditions. Values are an average of 2 biological replicates. Error bars indicate standard deviation with a sample size of n=2. **b** -ΔΔCt for cells expressing mTurquoise under the control of P_{6CCL} promoter. Cycling times for mTurquoise RNA was compared against native housekeeping gene UBC6, and conditions in 25% or 100% blue light were compared to dark conditions. Values are an average of 2 biological replicates. Error bars indicate standard deviation with a sample size of n=2.

The following edited paragraph has been made in the revised manuscript:

[Line 218] "BY474B showed increased expression of mTurquoise in blue light, saturating at 25% intensity with an induction fold of 30x (Fig. 2d). Similar to the red light system, we sought to compare its expression level to a commonly used native yeast promoter, to evaluate whether it is strong enough to carry out similar functions. Thus, P_{ACT1} was also used to express the mTurquoise reporter. Results showed that 100% intensity of blue light had a detrimental but unclear effect on the expression of P_{ACT1}, and further testing suggested this effect extended to many other native promoters. (Data and characterization of the effect of the

intense blue light on native promoters can be found in supplementary materials Annex P.) To verify that the blue light did not negatively impact the growth of cells, the OD_{600} of cultures was measured in various light conditions used in the study and the results showed that the growth of the cells was unaffected even under 100% blue light. It was also observed that similar to the y-iLight system's drop in expression, activity could be restored by cycling 30 minutes of 100% light and 30 minutes of darkness. As 100% blue light affected P_{ACT1}, we decided to limit our comparison of P_{6CCL} and P_{ACT1} to the range 0% to 50% blue light intensity including the dark condition which is the normal operating condition for P_{ACT1}. Within this range, P_{6CCL} at its maximum expression was comparable to that of P_{ACT1}, indicating that our blue light system could produce sufficient expression for similar applications."

All additional experiments done to study the phenomenon are included in the Annex P in supplementary material. Experiments that utilized P-PGK1 as a reference promoter have been re-done with P-ACT1, such as the characterizations of our light sensitive systems under different light intensities (revised Figure 2d) as well as the 56-hour experiments characterizing the systems across exponential, stationary and late stationary phase under darkness, 25% blue light, and 25% red light respectively (Supplementary Figures S2-6).

The following supplementary figures (Fig. S2-S6) are the figures that have been revised to include the results from the P-ACT1 native promoter which has replaced P-PGK1 used earlier:

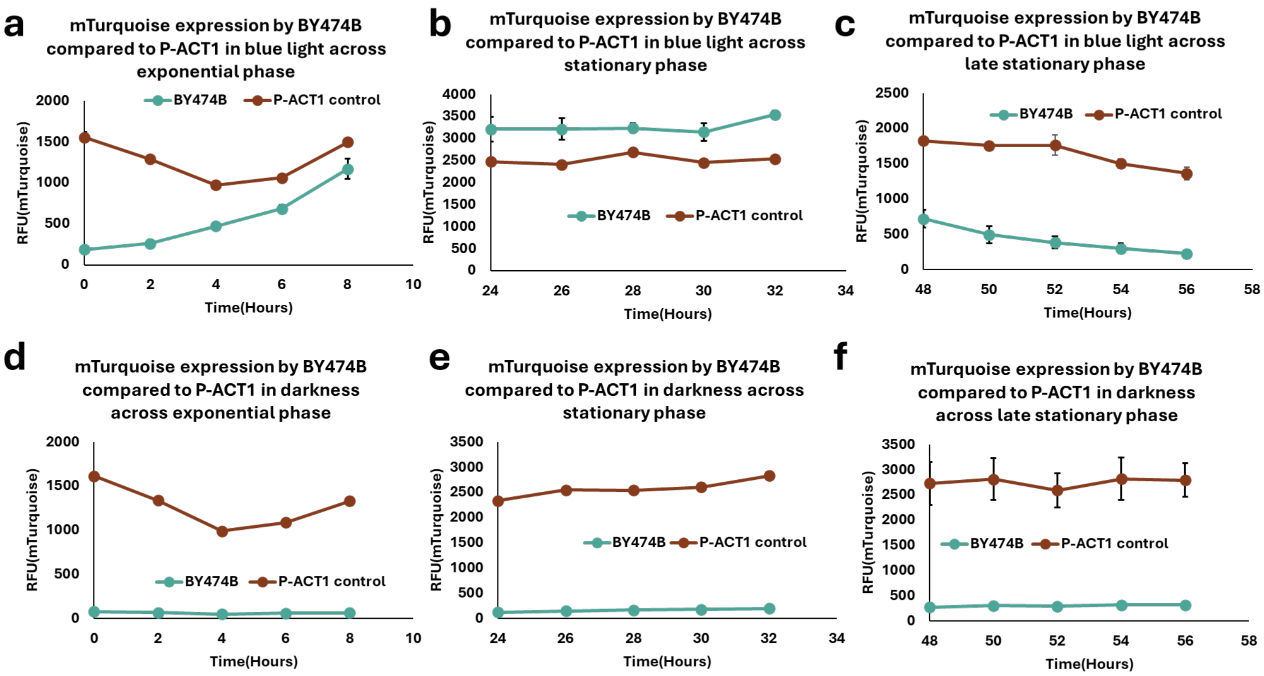

Supplementary Fig. S2: Activity of blue channel strain over exponential, stationary and late stationary phase

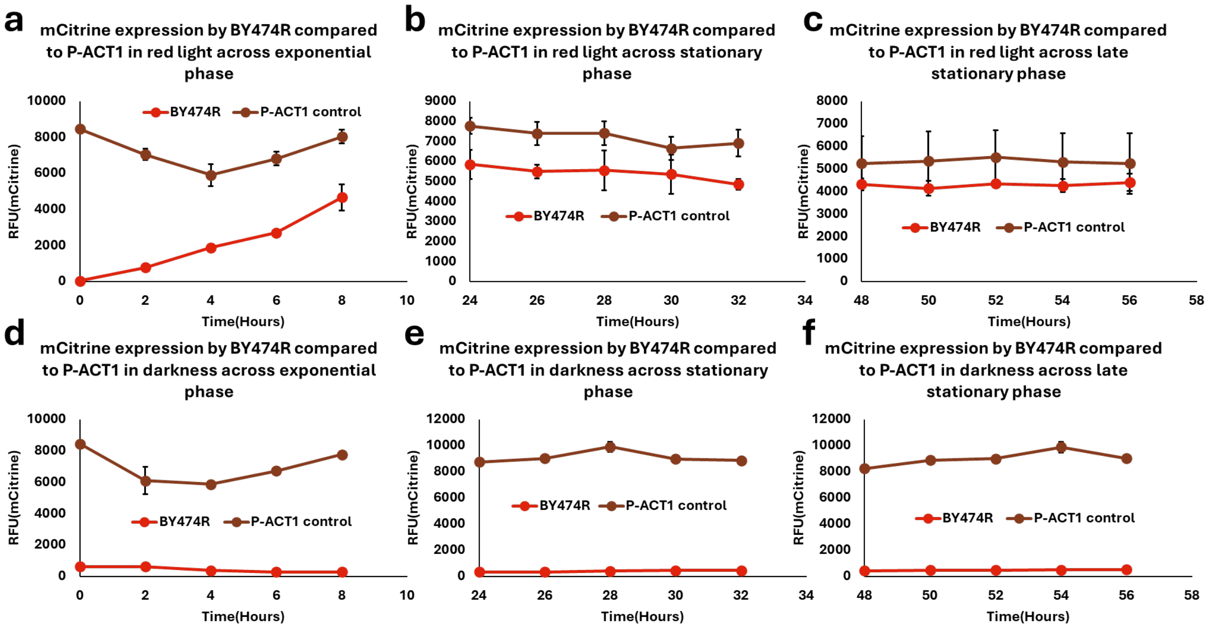

Supplementary Fig. S3: Activity of red channel strain over exponential, stationary and late stationary phase

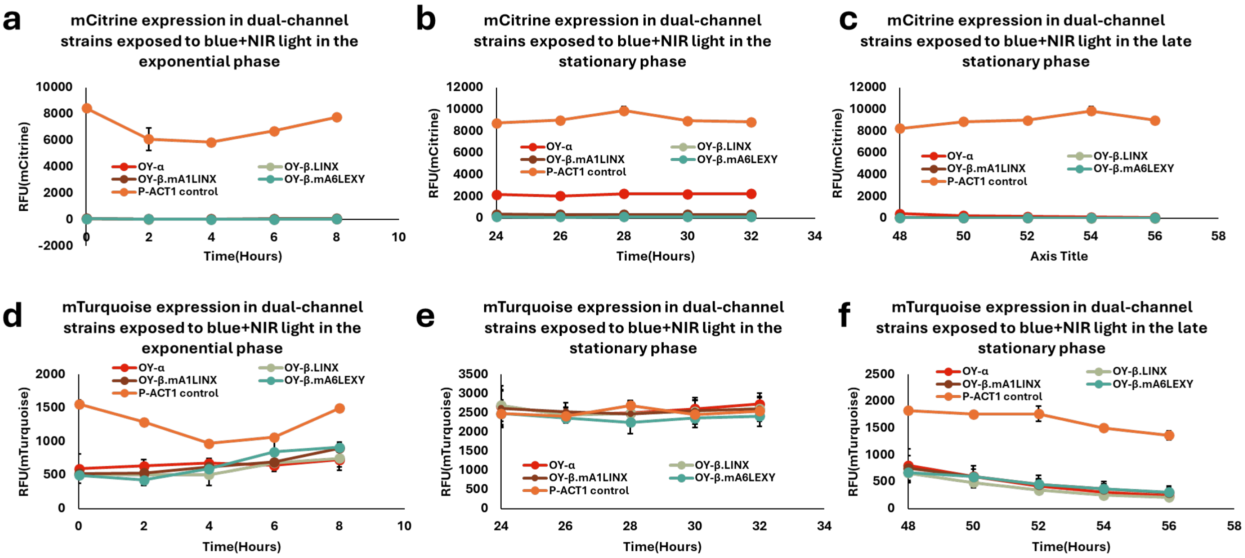

Supplementary Fig. S4: Activity of dual channel strains in blue plus NIR light over exponential, stationary and late stationary phase.

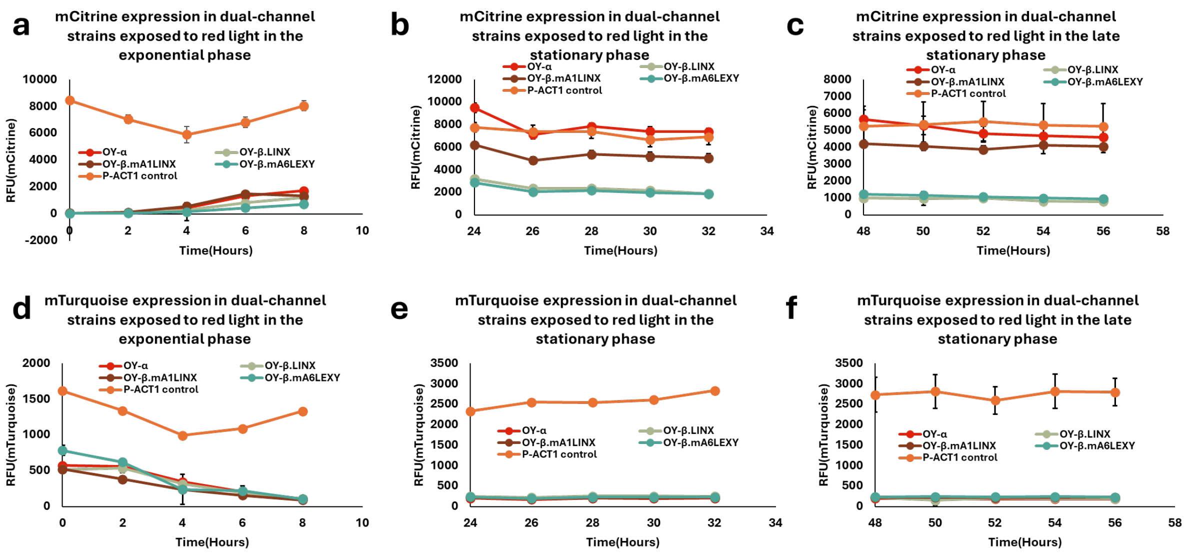

Supplementary Fig. S5: Activity of dual channel strains in red light over exponential, stationary and late stationary phase.

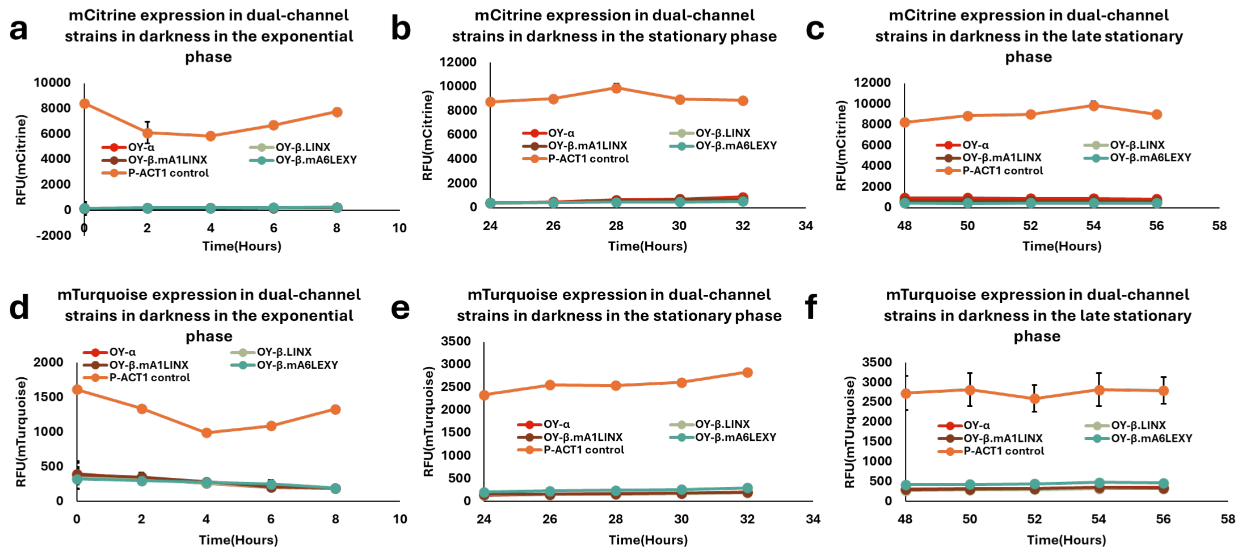

Supplementary Fig. S6: Activity of dual channel strains in darkness over exponential, stationary and late stationary phase.

Similarly, the reduced y-iLight activity at red-light intensities above 10% is attributed to steric hindrance within the promoter cassettes. This explanation highlights a design flaw that could, in principle, be improved through cassette optimization, yet no such optimization was attempted.

Reply: We acknowledge that the y-iLight system has room for improvement and that our hypothesis about the mechanism could be probed further to optimize it. However, the extensive promoter engineering that this would require is out of scope of our study. It has been shown that the promoter in its current form was able to function in the various applications that we tested, demonstrating multiplexed optogenetics in yeast which is the key objective of the paper.

Reviewer #4 (Remarks to the Author):

An ODE model was added to this manuscript in the review stage. The model captured light-induced gene expression and metabolic dynamics, using simple approximations: linear dependence of expression on light intensity, and Michaelis-Menten kinetics for the metabolic reactions.

The model is straightforward and generally sound. By using simple approximations, the number of fitted parameters is limited, leading to a decreased likelihood of overfitting. There are some potentially suboptimal aspects of the implementation, including the assumption that translation processes, RNA degradation processes, and DNA degradation processes all have the same first-order kinetic constants. Regardless of whether they should be first order, the fact that these processes are assumed to have the same constants even for the degradation/production of different proteins is unlikely to be precise. However, this is not a fatal flaw, and instead it only adds extra restrictions on the model that make it harder for the model to be accurate. This makes any accuracy in predictions actually more impressive. Of those three assumptions, the protein production rate is the least likely to be true and most likely to have an impact based on ribosome preferences for different transcripts.

The only significant concern is that the authors fixed the mRNA degradation rate. While they clearly indicated this in the supplement, there is no explanation of why (nor is there an obvious reason why), nor was this decision stated or described in the methods. This should be fixed.

We thank the reviewer for the insightful comments. We have fixed the mRNA degradation rate in our model because mRNA typically degrades much faster than protein and with only protein measurements available, fixing this parameter can reduce non-identifiability and enable more reliable estimation of the protein degradation rate, improving model capability to better capture and predict protein dynamics under different light patterns. Hence, we fixed the mRNA degradation rate using typical mRNA half-lives earlier reported (median ~20 min).

The following sentence has been added to the Methods section under Computational Kinetics Profiling in the revised manuscript:

[Line 969] “Since mRNA typically degrades much faster than protein and with only protein measurements available, we fixed the mRNA degradation rate (half-life is about 20 min[8]) to reduce non-identifiability and enable more reliable estimation of protein degradation rate while capturing or predicting protein dynamics under different light patterns.”

8. Wang, Y. et al. Precision and functional specificity in mRNA decay. *Proc Natl Acad Sci U S A* **99**, 5860–5865 (2002).

Also of note, the submitted code has the actual parameter fitting function commented out and the "best fit" parameters hard-coded in. While straightforward for an interested user to adjust, this is not what one would consider to be a "best practice".

We have modified the code file to allow the user to run the model fitting and commented out the best-fitted parameters.

The fact that the model has reasonably good predictivity for test data on which it was not trained suggests that the model reasonably accurately captures the biology, supporting the validity of analysis and interpretation of the kinetic parameters as the authors have done.

We thank the reviewer for the comment.

References:

1. Mazzoni, C., Torella, M., Petrera, A., Palermo, V. & Falcone, C. PGK1, the gene encoding the glycolitic enzyme phosphoglycerate kinase, acts as a multicopy suppressor of apoptotic phenotypes in S. cerevisiae. *Yeast* **26**, 31–37 (2009).
2. Karlsson, R., Aspenström, P. & Byström, A. S. A Chicken β-Actin Gene Can Complement a Disruption of the Saccharomyces cerevisiae ACT1 Gene. *Molecular and Cellular Biology* **11**, 213–217 (1991).
3. Delgado, M. L. *et al.* The glyceraldehyde-3-phosphate dehydrogenase polypeptides encoded by the Saccharomyces cerevisiae TDH1, TDH2 and TDH3 genes are also cell wall proteins. *Microbiology* **147**, 411–417 (2001).

4. Schirmaier, F. & Philippsen, P. Identification of two genes coding for the translation elongation factor EF-1 alpha of S. cerevisiae. *EMBO J* **3**, 3311–3315 (1984).
5. Jauniaux, J.-C. & Grenson, M. GAP1, the general amino acid permease gene of Saccharomyces cerevisiae. *European Journal of Biochemistry* **190**, 39–44 (1990).
6. Ah Kang, H. *et al.* Characteristics of Saccharomyces cerevisiae gal1Δ and gal1Δhxk2Δ mutants expressing recombinant proteins from the GAL promoter. *Biotechnology and Bioengineering* **89**, 619–629 (2005).
7. de Smidt, O., du Preez, J. C. & Albertyn, J. The alcohol dehydrogenases of Saccharomyces cerevisiae: a comprehensive review. *FEMS Yeast Res* **8**, 967–978 (2008).
8. Wang, Y. et al. Precision and functional specificity in mRNA decay. *Proc Natl Acad Sci U S* A **99**, 5860–5865 (2002).
